# Autogenous Production and Stabilization of Highly Loaded Sub‐Nanometric Particles within Multishell Hollow Metal–Organic Frameworks and Their Utilization for High Performance in Li–O_2_ Batteries

**DOI:** 10.1002/advs.202000283

**Published:** 2020-03-16

**Authors:** Won Ho Choi, Byeong Cheul Moon, Dong Gyu Park, Jae Won Choi, Keon‐Han Kim, Jae‐Sun Shin, Min Gyu Kim, Kyung Min Choi, Jeung Ku Kang

**Affiliations:** ^1^ Department of Materials Science and Engineering and NanoCentury KAIST Institute Korea Advanced Institute of Science and Technology (KAIST) 291 Daehak‐ro, Yuseong‐gu Daejeon 34141 Republic of Korea; ^2^ Department of Chemistry Korea Advanced Institute of Science and Technology (KAIST) 291 Daehak‐ro, Yuseong‐gu Daejeon 34141 Republic of Korea; ^3^ Pohang Accelerator Laboratory (PAL) Korea Pohang University of Science and Technology 77 Cheongam‐ro, Namg‐gu Pohang 37673 Republic of Korea; ^4^ Department of Chemical and Biological Engineering Sookmyung Women's University Cheongpa‐ro 47‐gil 100, Yongsan‐gu Seoul 04310 Republic of Korea; ^5^ Graduate School of Energy Environment Water and Sustainability (EEWS) Korea Advanced Institute of Science and Technology (KAIST) 291 Daehak‐ro, Yuseong‐gu Daejeon 34141 Republic of Korea

**Keywords:** Li–O_2_ batteries, metal–organic frameworks, sub‐nanometric particles, water molecule transfer

## Abstract

Sub‐nanometric particles (SNPs) of atomic cluster sizes have shown great promise in many fields such as full atom‐to‐atom utilization, but their precise production and stabilization at high mass loadings remain a great challenge. As a solution to overcome this challenge, a strategy allowing synthesis and preservation of SNPs at high mass loadings within multishell hollow metal–organic frameworks (MOFs) is demonstrated. First, alternating water‐decomposable and water‐stable MOFs are stacked in succession to build multilayer MOFs. Next, using controlled hydrogen bonding affinity, isolated water molecules are selectively sieved through the hydrophobic nanocages of water‐stable MOFs and transferred one by one to water‐decomposable MOFs. The transmission of water molecules via controlled hydrogen bonding affinity through the water‐stable MOF layers is a key step to realize SNPs from various types of alternating water‐decomposable and water‐stable layers. This process transforms multilayer MOFs into SNP‐embedded multishell hollow MOFs. Additionally, the multishell stabilizes SNPs by π‐backbonding allowing high conductivity to be achieved via the hopping mechanism, and hollow interspaces minimize transport resistance. These features, as demonstrated using SNP‐embedded multishell hollow MOFs with up to five shells, lead to high electrochemical performances including high volumetric capacities and low overpotentials in Li–O_2_ batteries.

## Graphical abstract

Hydrogen affinity‐controlled water molecule transfers throughout multilayer metal–organic frameworks result in sub‐nanometric particle (SNP)‐embedded multishell hollow metal–organic frameworks (MOFs), where the multishell stabilizes SNPs by π‐backbonding allowing high conductivity and hollow interspaces that minimize transport resistance. Moreover, increasing the mass loading of SNPs within multishell hollow MOFs is demonstrated to enable higher capacities and smaller overpotentials in Li–O_2_ batteries.

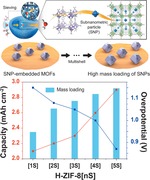

Despite sub‐nanometric particles (SNPs) having unique abilities such as maximized atom‐utilization efficiency and governable catalytic selectivity,^[^ ^1^, ^2^ ^]^ a breakthrough solution that allows not only to originate SNPs and also to preserve single or a few atoms is still required. SNPs are typically stabilized through agglomeration due to their unsaturated surface bonds,^[^ ^3^ ^]^ even after being synthesized by complex processes, so that the strong interactions with solid supports have been quite successful in stabilizing them.^[^ ^4^, ^5^, ^6^ ^]^ Meanwhile, this method was possible only at low mass loadings where the collision frequency of SNPs can be inhibited.^[^ ^7^ ^]^ In another way, the pyrolysis of organometallic compounds induces the formation of heterogeneous SNPs at nonuniform particle sizes.^[^ ^8^ ^]^ However, as agglomeration is driven by surface energy differences attributed to different particle sizes,^[^ ^9^ ^]^ these heterogeneous SNPs also grow to become larger particles.

We hypothesize that the key requisite to make homogeneous and robust SNPs is a chemically designable system providing manipulative formation and simultaneous stabilization of SNPs. In order that the system is effectively implemented, it should have a high porosity and a large surface area for high SNP loading. Metal–organic frameworks (MOFs) provide chemical flexibility and high porosity,^[^ ^10^ ^]^ such that stable MOFs allow the controlled mass transfer and quantitative stabilization of atomic clusters, while the constituents of decomposable MOFs can work as precursors for SNPs. Accordingly, there were many great efforts to utilize MOFs as materials for preparing various shaped structures with advanced functionalities.^[^ ^11^, ^12^, ^13^, ^14^, ^15^, ^16^ ^]^ In this work, we develop multilayer MOFs with alternating stable and decomposable layers for the autogenous production and stabilization of SNPs in scalable mass loadings. Interestingly, we also show that the single water molecule transfer throughout multilayer MOFs plays a very important role to activate the autogenous synthesis of SNPs. The step‐by‐step deterioration of a decomposable MOF layer by the single water molecule leads to the construction of uniform atomic clusters, thus promoting the synthesis of SNPs in the stable MOF layer. It is also investigated how to introduce the single water molecules in multilayer MOFs and study the interaction between the single water molecule and its environment as a key factor. Finally, the higher mass loading of SNPs in multishell MOFs is realized by the autogenous production and stabilization of dinuclear SNPs with multishell hollow MOFs, and they are applied as electrocatalysts to realize high performance in Li–O_2_ batteries.

The strategy for autogenously producing and stabilizing SNPs inside MOFs is shown in **Figure** **1**a. First, alternating water‐stable and water‐decomposable MOFs were stacked in multilayers using the same organic linkers. Next, water molecules need to be transferred to the decomposable MOF layer throughout the stable MOF layer to activate the autogenous production of SNPs. The high hydrogen bonding affinity between ethylene glycol (EG) and water molecules was used to isolate each water molecule,^[^ ^17^, ^18^, ^19^ ^]^ thus providing the manipulative formation of SNP precursors while the water clusters formation is blocked. Owing to their hydrophobicity, the stable MOF layers effectively transferred water molecules to the decomposable MOF layers without adsorption of water molecules. Subsequently, SNPs derived from the decomposable MOFs were stabilized inside the pores of the water‐stable MOFs. We chose zeolitic imidazolate framework‐8 (ZIF‐8) and ZIF‐67 as water‐stable and water‐decomposable MOFs, respectively. ZIF‐8 enabled water molecules to be fed one by one, because the penetration of the isolated water molecule could be manipulated by sieving EG–water complexes through hydrophobic micropores. The X‐ray diffraction (XRD) patterns of ZIF‐67 after hydrolysis (Figure S1, Supporting Information) and the unchanged XRD patterns of ZIF‐8 (Figure S2, Supporting Information) demonstrate that all the metal complexes in ZIF‐67 are disconnected from the organic linkers when exposed to water. ZIF‐67, which is isostructural with ZIF‐8 because both MOFs are composed of 2‐methylimidazole (2‐mim),^[^ ^20^ ^]^ was seeded for the epitaxial growth of ZIF‐8 to construct multilayer ZIFs (ML‐ZIFs[*n*L], where *n* is the number of layers) (Figure 1bI). The boundary between ZIF‐67 and ZIF‐8 is indicated by the dotted line in the high‐angle annular dark‐field scanning transmission electron microscopy (HAADF‐STEM) image (Figure 1bII) and by the distinct colors corresponding to Co in ZIF‐67 and Zn in ZIF‐8 revealed by the energy‐dispersive X‐ray spectroscopy (EDX) (Figure 1bIII; Figure S3, Supporting Information). Figure 1cI shows that ML‐ZIFs[*n*L] was transformed into SNP‐embedded multishell hollow ZIF‐8 (H‐ZIF‐8[*n*S], where *n* is the number of shells). The disassembly of the bonds between the Co ions and 2‐mim in ZIF‐67 generates SNP precursors and interspaces, as identified by the elemental mapping and HAADF‐STEM images (Figure 1cII,III; Figure S4, Supporting Information). The line profiling analysis (Figure S5, Supporting Information) clarifies that the Co species migrated into the ZIF‐8 shell. The inductively coupled plasma optical emission spectroscopy (ICP‐OES) and EDX analyses (Tables S1–S3, Supporting Information) show that H‐ZIF‐8[1S] has a Co content of 10.4 wt%, indicating that the water‐decomposable MOFs allow the high mass loading of Co particles in the water‐stable MOFs. Moreover, the transmission electron microscopy (TEM) images (Figure 1d; Figures S6–S10, Supporting Information) demonstrate that H‐ZIF‐8[2S, 3S, 4S, 5S] are created after sieving EG–water complexes through ML‐ZIFs[4L, 6L, 8L, 10L]. These results reveal that multishell hollow MOFs could be produced via the controlled transfer of isolated water molecules from EG–water complexes through multilayer MOFs.

**Figure 1:**
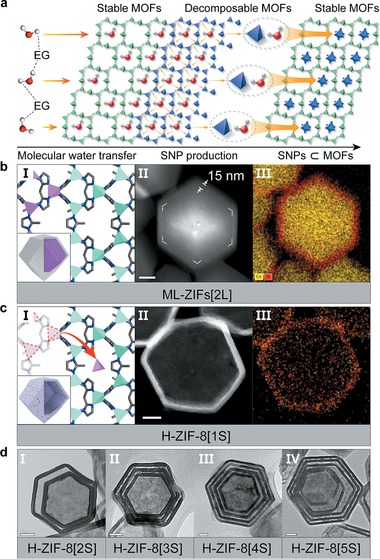
Strategy for the synthesis of SNPs and the structural characterization of H‐ZIF‐8[*n*S]. a) Schematic of the formation process of SNP‐embedded MOFs, where different colors signify different metal nodes. bI,cI) Illustrations of multilayer MOFs and multishell with SNPs (green: Zn, violet: Co). bII,cII) HAADF‐STEM images of ML‐ZIFs[2L] and H‐ZIF‐8[1S]. bIII,cIII) Elemental mapping images of ML‐ZIFs[2L] and H‐ZIF‐8[1S]. dI, dII, dIII, dIV) TEM images of H‐ZIF‐8[2S, 3S, 4S, 5S]. All scale bars are 50 nm.

To elucidate the chemical state of Co SNPs in the ZIF‐8 shell, the X‐ray photoelectron spectroscopy (XPS) measurements were conducted. No Co 2p characteristics are observed in ML‐ZIFs[2L] (Figure S11, Supporting Information), but the XPS peaks of H‐ZIF‐8[1S] confirm the existence of divalent Co atoms comparable to those in a bulk Co(OH)_2_. To further clarify the different structures of ML‐ZIFs[2L] and H‐ZIF‐8[1S], the Co K‐edge X‐ray absorption near‐edge structure (XANES) spectra were collected (**Figure** **2**a), which revealed three noticeable changes. First, the pre‐edge feature of H‐ZIF‐8[1S], marked by * around 7709 eV, is significantly reduced. Second, the shoulder absorption edge caused by the partially localized excitation of Co—N bonds^[^ ^21^ ^]^ disappears, as indicated by an arrow around 7720 eV. Finally, the main absorption peak shifts to higher photon energy. The pre‐edge peak of ML‐ZIFs[2L] corresponds to the 1s–3d electric dipole forbidden transition originating from tetrahedrally coordinated Co in ZIF‐67, whereas that of bulk Co(OH)_2_ becomes almost flat with the formation of centrosymmetric edge‐shared CoO_6_ octahedra. Thus, the geometric transformation from ML‐ZIFs[2L] to H‐ZIF‐8[1S] results in a transition of Co coordination from tetrahedral to distorted octahedral, attributable to 3d–4p orbital mixing initiated by the slightly tilted centrosymmetric coordination.^[^ ^22^ ^]^ Moreover, Co atoms are more oxidized through the formation of Co—O bonds, thereby enhancing the 1s–4p transition on back scattering^[^ ^23^ ^]^ induced by the structural transformation, resulting in the disappeared shoulder edge and higher energy shift. The red area in the 2D contour map (Figure S12, Supporting Information) indicates the formation of a higher oxidation state after the structural transformation of ML‐ZIFs[2L] to H‐ZIF‐8[1S]. The coordination environment of Co was explored using the K‐edge extended X‐ray absorption fine structure (EXAFS) spectroscopy (Figure 2b). The main peak of ML‐ZIFs[2L] at 1.65 Å is attributable to Co—N bonds and the peaks at 2.23, 2.67, 2.94, and 3.71 Å correspond to 2‐mim. However, H‐ZIF‐8[1S] has two peaks corresponding to Co—OH bonds at 1.70 Å, as supported by the IR and XPS spectra^[^ ^24^, ^25^ ^]^ (Figures S13 and S14, Supporting Information), and to oxygen edge‐sharing Co—Co bonds at 2.90 Å. The changes in the coordination environment during the transformation of ML‐ZIFs[2L] to H‐ZIF‐8[1S] can be confirmed by the disappearance of the 2‐mim signals and the appearance of only two peaks (Figure S15, Supporting Information). The Co–Co peak is significantly weaker than the Co–OH peak, which indicates the existence of atomic particles.^[^ ^26^ ^]^ In the Co K‐edge simulation curves (Figure 2c; Figure S16, Supporting Information), the ratio between Co–OH peak and Co–Co peak demonstrates that Co(OH)_2_ is dinuclear in H‐ZIF‐8[1S] (Table S4, Supporting Information). The XANES and EXAFS spectra (Figures S17 and S18, Supporting Information) verify that Zn is not affected in the process. These results are consistent with the effective disconnection of all the coordinate bonds of ZIF‐67 and the generation of dinuclear Co(OH)_2_ SNPs inside the micropores of H‐ZIF‐8[*n*S]. In addition, the “disordered‐to‐crystalline” phenomenon^[^ ^27^ ^]^ involves agglomeration of SNPs upon exposure to a highly enhanced electron beam, thereby corroborating the existence of SNPs inside the damaged multishell hollow MOFs (Figure S19 and Video S1, Supporting Information).

**Figure 2:**
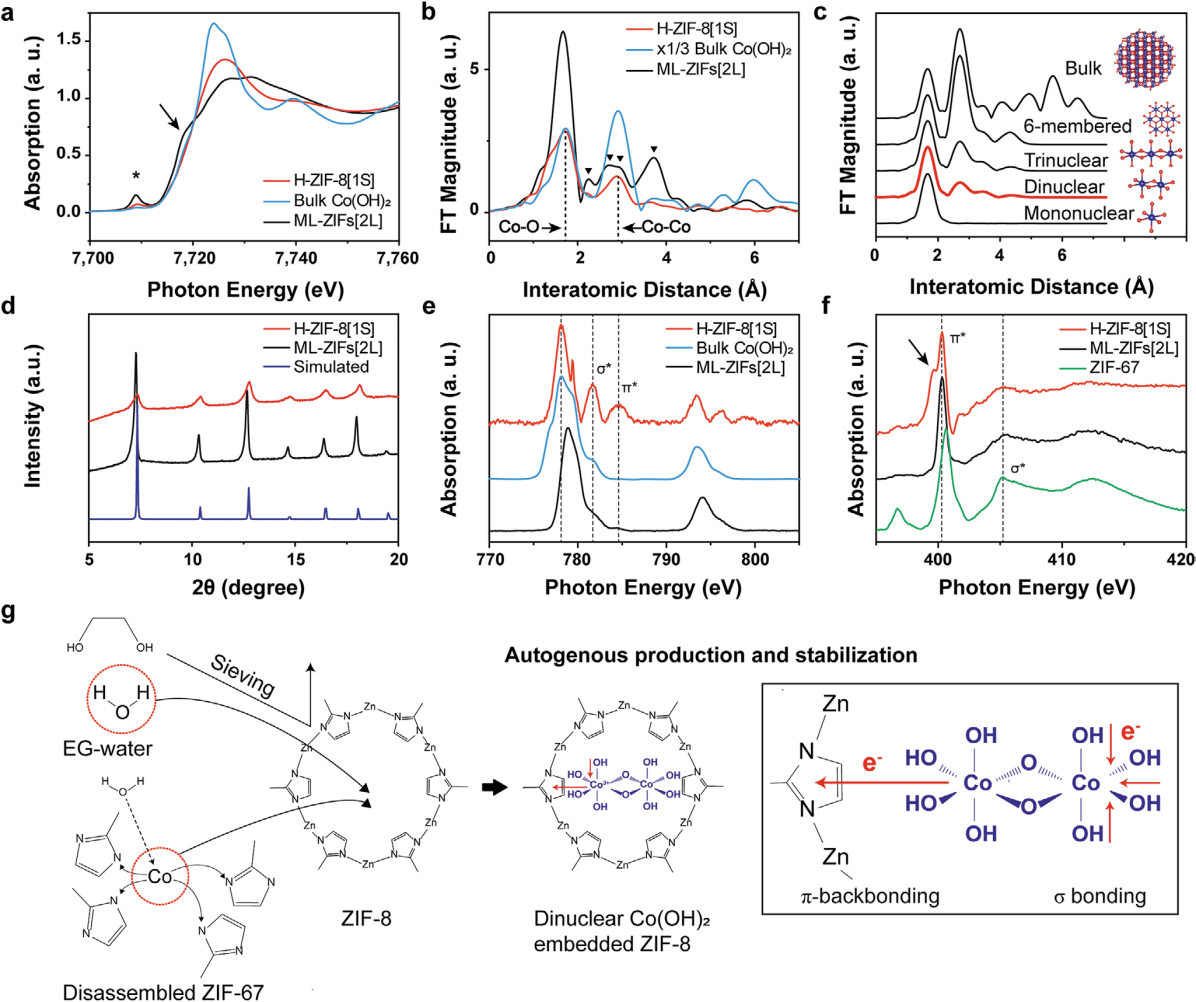
Characterization of Co(OH)_2_ SNPs. a) Normalized Co K‐edge XANES spectra. b) Radial distribution function obtained by the Fourier transformation of k^3^‐weighted Co EXAFS spectra. c) Co K‐edge EXAFS simulation curves with Co(OH)_2_ size obtained by back Fourier transformation of the radial structural function. d) XRD patterns with simulated patterns of pristine ZIF‐8. e) Normalized Co L‐edge NEXAFS spectra. f) Normalized N K‐edge NEXAFS spectra. g) Illustration of the autogenous production and stabilization of Co(OH)_2_ SNPs in a micropore.

The role of each micropore was also confirmed by the changes in the physical and chemical properties of H‐ZIF‐8[1S]. The XRD patterns of ZIF‐8 agree with the simulated patterns of H‐ZIF‐8[*n*S] at diffraction angles lower than 20° (Figure 2d; Figure S20, Supporting Information). However, the peaks associated with the {011} facets shift toward higher angles and exhibit reduced intensities. The scanning electron microscopy (SEM) images (Figures S21 and S22, Supporting Information) reveal that ZIF‐8 has a rhombic dodecahedron morphology with exposed {011} facets that form 1.1 nm micropores connected to 0.34 nm apertures.^[^ ^28^ ^]^ The micropore is induced by the {011} facets allowing the passage of only a single water molecule.^[^ ^29^ ^]^ Thus, the formation of Co(OH)_2_ SNPs in micropores results in a shift to higher angles owing to the slightly reduced pore sizes, with the reduced intensities for the {011} facets. The decreased pore volumes and pore sizes in the Brunauer–Emmett–Teller (BET) isotherms as well as the pore size distribution curves also indicate the micropore filling by the Co(OH)_2_ SNPs (Figures S23 and S24, Supporting Information). The near‐edge X‐ray absorption fine structure (NEXAFS) spectra showed a changed dipole transition from core electrons to unoccupied molecular orbitals caused by the micropore filling. The trigonal or tetragonal distortions of octahedral bulk Co(OH)_2_ lead to t_2g_ and e_g_ orbital splitting, as represented by Co L_2,3_‐edge multiplets^[^ ^30^ ^]^ (Figure 2e). This splitting completely disappears at 776.9 and 779.7 eV in the H‐ZIF‐8[1S] spectrum, but a dominant dipole transition at 778.1 eV, which is related to the polarized orbital occupation resulting from the spatial strain in confined spaces,^[^ ^31^, ^32^ ^]^ is observed. Interestingly, we observe a narrow, strong peak at 779.4 eV owing to the transition of more oxidized Co^3+^ (t_2g_^6^ e_g_^0^, low‐spin) to the higher energy e_g_ orbital, which is distinguishable from Co^2+^ (t_2g_^5^ e_g_^2^, high‐spin). The blue‐shifted IR spectrum (Figure S25, Supporting Information) supports the presence of Co^3+^ in H‐ZIF‐8[1S].^[^ ^33^ ^]^ The electron‐deficient Co^3+^ strongly attracts hydroxyl group lone pair electrons to fill the completely empty e_g_ orbitals, as confirmed by the strong σ* transition at 781.7 eV. The enriched σ bonds also result in shorter bond lengths (Figure 2b), as indicated by the shoulder below 1.70 Å.^[^ ^34^ ^]^ Generally, the donor atoms involved in σ bonds become more electropositive, thereby allowing π‐backbonding from the completely filled metallic t_2g_ orbital to the empty π* orbital of the donor atom.^[^ ^35^ ^]^ Commonly, π‐backbonding occurs between transition metals and organic ligands, but the predominant π* transition at 784.8 eV for H‐ZIF‐8[1S] indicates an increase in π‐backbonding^[^ ^36^ ^]^ not observed in bulk Co(OH)_2_ and ML‐ZIFs[2L]. The N K‐edge NEXAFS spectrum of H‐ZIF‐8[1S] (Figure 2f) shows a split π* transition, which is typically observed in extended π–π* systems, with π orbitals accessible to the K‐edge excited electrons^[^ ^37^ ^]^ at 400.3 and 399.7 eV (marked by arrows). Imidazole rings are stronger π‐acceptors than hydroxyl groups, so that the −0.7 eV shift of the π* transition is derived from the increased electron density resulting from π‐backbonding by the surrounding Co(OH)_2_ SNPs. Furthermore, preferential π‐backbonding between Co(OH)_2_ SNPs and 2‐mim lowers the energy level of the t_2g_ orbital. The NEXAFS spectra show 22.5% Co^3+^ and 44.6% π‐backbonding, suggesting that Co(OH)_2_ SNPs are stabilized in H‐ZIF‐8[1S] (Figures S26 and S27, Supporting Information). The splitting of the π* transition indicates that Co is not substituted at the Zn tetrahedral site in ZIF‐8, as supported by the fingerprint region of the IR spectrum (Figure S28, Supporting Information), which shows the N–Zn–N stretching mode at 420 cm^−1^ without the N–Co–N stretching mode at 424 cm^−1^. It has been reported that the lower electron density of Co results in a shift of 0.3 eV toward higher energies relative to Zn, which is more electron dense,^[^ ^38^ ^]^ as observed in the case of ZIF‐67 and ML‐ZIFs[2L]. These observations are also supported by the absence of changes in the C 1s, N 1s, and Zn 2p XPS spectra and the Zn L‐edge NEXAFS spectra (Figures S29–S32, Supporting Information).

The transmission of isolated water molecules via controlled hydrogen bonding affinity through the micropores is the key factor in enabling the autogenous production and stabilization of SNPs within multishell MOFs (**Figure** **3**a). When water clusters react without EG, H‐ZIF‐8[1S] is not generated (Figure 3b) because the water clusters are too large to pass through the ZIF‐8 aperture.^[^ ^39^ ^]^ Solvated Co ions also do not penetrate the aperture because fully solvated Co ions are larger than the aperture (Figure S33, Supporting Information). For this reason, the strong hydrogen bonding affinity between EG and water molecules was exploited to isolate water molecules and allow them to pass through the 0.34 nm aperture of ZIF‐8. Using the isolation approach, exposure to relatively large or small amounts of water produces sheet‐like materials similar in structure to bulk Co(OH)_2_ or voids owing to the lack of water in ML‐ZIFs[2L] (Figure 3b). With the optimal amount of water, the disassembled Co ions aggregate into Co ion clusters via ion–dipole interactions,^[^ ^40^ ^]^ as verified by the bright spots in the HAADF‐STEM image during the intermediate stage (Figure S34, Supporting Information). Then, Co atoms in the clusters are laminated on the inner shell surface and pass through the micropores, suppressing the growth of sheet‐like structures (Figure S35, Supporting Information). The limited amount of water prevents complete hydration and regulates Co ion nucleation. Co ions are fed into the micropores prior to bulk Co(OH)_2_ formation and then form dinuclear Co(OH)_2_ SNPs as water molecules are supplied one by one into the micropores. To confirm the isolation of water molecules by EG, we used the nuclear magnetic resonance (NMR) spectroscopy. EG has exchangeable hydroxyl groups that are indistinguishable on the ^1^H‐NMR time scale (Figure S36, Supporting Information). However, a strong peak is observed at 5–6 ppm when water molecules are added to EG (Figure S37, Supporting Information). The ^1^H–^1^H correlation spectroscopy (COSY), which can explain a spin–spin coupling between EG and water molecule, was conducted to find the reason for newly appeared peak by interpretation of the nature of the EG–water complex (Figures S38 and S39, Supporting Information). The cross‐peaks, denoted as “OH‐H_2_O” (Figure 3c), demonstrate that the formation of bonds between water and EG occurs via the donation of water hydrogen atoms to the oxygen of EG.^[^ ^41^ ^]^ The ^13^C‐NMR spectra (Figure 3d) show a broad peak at 63.3 ppm for the EG–water complex (intermolecular form), which is distinct from the sharp peak at 63.7 ppm for a pure EG (intramolecular form). We found that the intramolecular form transforms into the intermolecular form in the presence of water molecules and water isolation proceeds effectively, as evidenced by the fixed hydroxyl group and the interaction between EG and water molecule (Figures S40 and S41, Supporting Information). The two forms behave differently owing to their distinct structures. The broadness of the ^13^C‐NMR peak originates from a slower tumbling rate owing to a large molecular size. The ^13^C diffusion‐ordered spectroscopy (DOSY) was also utilized to determine the cluster size. The diffusion coefficient calculated for the intramolecular form indicates minor interactivity, as the value is not affected significantly by the presence of water. In contrast, the value determined for the intermolecular form is approximately 20‐fold higher than that for the intramolecular form, indicating that the intermolecular form behaves like a huge cluster similar to a structure of consecutively bridged water molecules (Table S5, Supporting Information). As a result of this huge EG–water cluster, the isolated water molecules come into contact with the decomposable MOFs one by one, which prevents supersaturation of the reactants and thus avoids rapid nucleation. This approach not only makes hydrolysis controllable by regulating molecular water transfer, but also effectively suppresses nucleation, resulting in the autogenous production of uniform SNPs at high mass loadings. This method can be used to synthesize multishell MOFs via selective detachability, even in the presence of SNPs, which has previously been difficult to implement. After water molecule transfer, the ^1^H‐NMR and ^13^C‐NMR spectra show weakening of the hydroxyl group peak and disappearance of the water and intermolecular peaks (Figure 3e; Figure S42, Supporting Information), indicating that the intermolecular form returns to the original intramolecular form through reversion of the conformational changes caused by the presence of a water molecule (Figure S43, Supporting Information).

**Figure 3:**
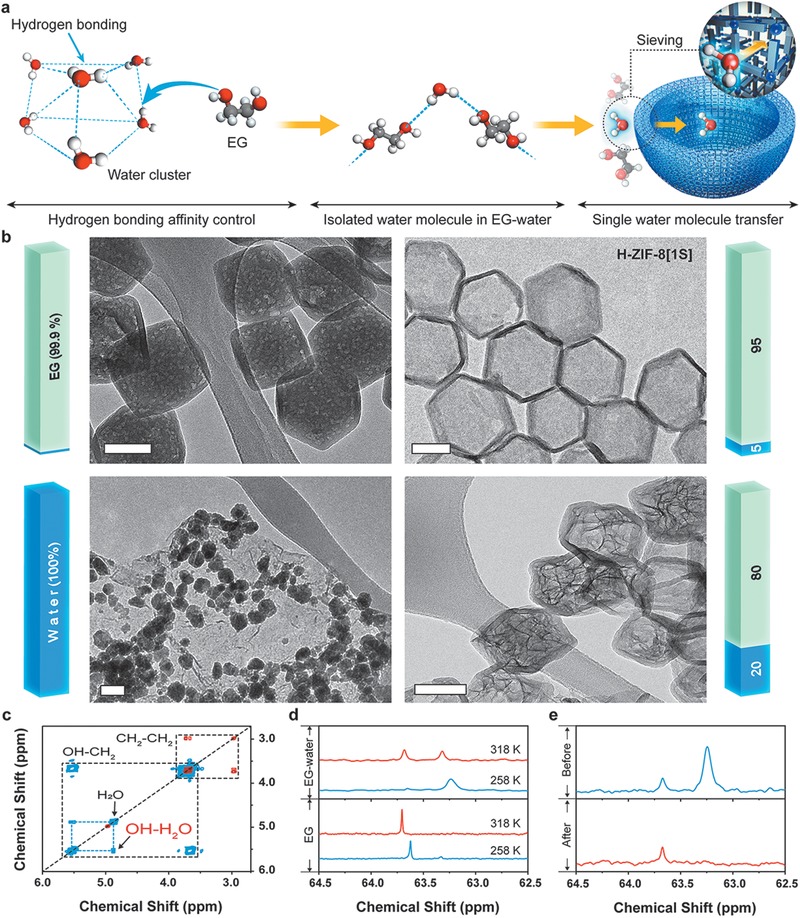
Formation and penetration mechanisms of an isolated water molecule. a) Schematic of the formation process of EG–water complexes and illustration of the penetration process of an isolated water molecule. b) TEM images showing structural changes of ML‐ZIFs[2L] depending on transferred water volume ratio in EG–water complexes. All scale bars are 100 nm. c) COSY spectra showing spin–spin coupling between the protons of EG–water complexes at 258 and 318 K. d) ^13^C‐NMR spectra of pure EG and EG–water complexes at 258 and 318 K. e) ^13^C‐NMR spectra after water molecules transfer at 273 K.

There are two strategies to increase the active sites for electrocatalysis, which include i) exposing more active sites or/and ii) loading more active materials.^[^ ^42^ ^]^ We increase the number of active sites by increasing the exposed active sites through the synthesis of dinuclear Co(OH)_2_ SNPs, while simultaneously loading more active materials into the multishell structures (**Figure** **4**a). On the basis of the strategically‐designed system, we evaluated the dependence of the electrocatalytic performance of a Li–O_2_ battery on Co(OH)_2_ SNPs and multishell structures. For pristine ZIF‐8, the charging curve shows a high overpotential with a potential gap of 1.494 V at half‐capacity (Figure 4b). The Co(OH)_2_ SNPs in H‐ZIF‐8[1S] remarkably improve the overpotential for the oxygen evolution reaction (OER) upon charging. The potential gap of 0.540 V at half‐capacity is approximately 63.9% lower than that of pristine ZIF‐8. Notably, the oxo groups between the Co ions promote the rapid oxidization of Co^2+^ to Co^4+^, facilitating the OER.^[^ ^43^, ^44^ ^]^ The Co^3+^ content of 22.5% enhances the OER kinetics by providing a shortcut to Co^4+^ and more active sites.^[^ ^45^ ^]^ Although bulk Co(OH)_2_ exhibits an improved overpotential, the discharge potential gradually decreases, whereas H‐ZIF‐8[1S] sustains a higher voltage (Figure S44, Supporting Information). This result indicates that bulk Co(OH)_2_ is a good electrocatalyst for the OER upon charging, but unsuitable for the oxygen reduction reaction (ORR) upon discharging. Meanwhile, H‐ZIF‐8[1S] not only provides a large surface area for the accumulation of the discharge product Li_2_O_2_ during the ORR, but also helps to decompose Li_2_O_2_ efficiently during the OER. The specific gravimetric capacity clarifies the importance of Co(OH)_2_ SNPs, but a smaller amount of H‐ZIF‐8[*n*S] is loaded as the number of shells increases at the same weight. In this reason, the specific geometric performance was utilized to determine the trend as the number of shell increases. The geometric performance (Figure 4c; Figure S45, Supporting Information) shows improved overpotentials under a constant areal current density, and the capacity also increases proportionally (Figure 4d; Figure S46 and Table S6, Supporting Information). To elucidate the origins for these enhancements, electrochemical impedance spectroscopy (EIS) measurements were performed. The Nyquist plot (Figure 4e) reveals two distinct changes that indicate improved performance. The charge‐transfer resistance (*R*_ct_) of H‐ZIF‐8[5s] is significantly smaller than that of pristine ZIF‐8, as is the solution resistance (*R*_s_) (Figure S47 and Table S7, Supporting Information). The reduced *R*_ct_ suggests that Co(OH)_2_ SNPs enhance the poor electrical conductivity of ZIF‐8 through the hopping transport mechanism.^[^ ^46^ ^]^ Furthermore, the reduced *R*_s_ indicates that the hollow structure minimizes transport resistance, as the diffusion time is inversely proportional to the diffusion length.^[^ ^47^ ^]^ The π‐backbonding between Co(OH)_2_ SNPs and micropores enhances stability owing to strong adhesion.^[^ ^48^ ^]^ Thus, a larger amount of Co(OH)_2_ SNPs within multishell ZIF‐8 should give excellent cycling stability, as demonstrated by the superiority of H‐ZIF‐8[5S] in Li–O_2_ batteries (Figure 4f). Notably, excellent cycling in Li–O_2_ batteries is also attributable to enhanced ORR and OER efficiencies. To investigate the structural stability of H‐ZIF‐8[1S] after electrochemical reactions, the XRD patterns were compared before and after 20 cycles (Figure S48, Supporting Information). The patterns were well maintained after 20 cycles, while the intensity associated with 011 facets was decreased due to the Li_2_O_2_ products of electrochemical reactions. This indicates that the structure of H‐ZIF‐8[1S] is stable during electrochemical reactions. Figure 4g shows that the capacity increases and the overpotential decreases at a higher mass loading with the same electrode volume, demonstrating that the SNP‐embedded multishell ZIF‐8 allows the scalable synthesis of electrocatalysts into electrodes.

**Figure 4:**
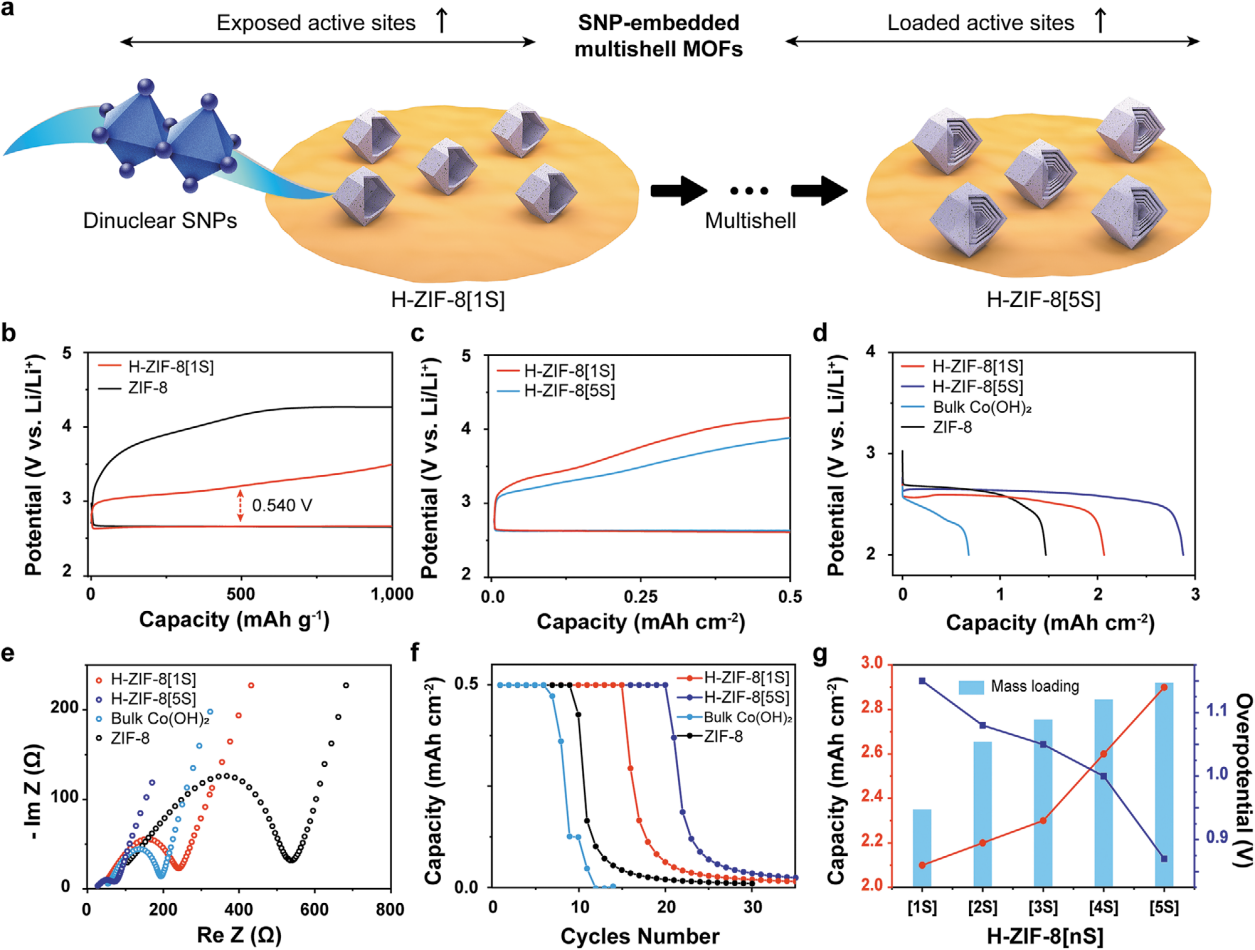
Performance improvement in the presence of Co(OH)_2_ and dependence on the number of multishell. a) Schematic of strategy to increase the active sites using dinulear SNPs and H‐ZIF‐8[*n*S]. b) Gravimetric discharge and charge curves of H‐ZIF‐8[1S] and ZIF‐8 at a current density of 50 mA g^−1^ with a cutoff capacity of 1000 mAh g^−1^. c) Geometric discharge and charge curves of H‐ZIF‐8[1S] and H‐ZIF‐8[5S] at a constant current of 0.1 mA cm^−2^ with a cutoff capacity of 0.5 mAh cm^−2^. d) Full geometric discharge curves at a constant current density of 0.1 mA cm^−2^. e) Nyquist plot corresponding to the EIS measurements conducted at 0.1–10^5^ Hz with an amplitude of 10 mV. f) Cyclability performance comparison plot at a constant current of 0.1 mA cm^−2^ with the cutoff capacity of 0.5 mAh cm^−2^. g) Comparison plot of capacity and overpotential according to mass loading.

Dinuclear Co(OH)_2_ SNPs within multishell MOFs were autogenously synthesized and stabilized in chemically designable system using multilayer MOFs. The strong hydrogen bonding affinity between EG and water effectively prevented the formation of water clusters and acted as a mediator to balance the nucleation and the growth of SNPs within multilayer MOFs. It was confirmed that the transfer of isolated water molecules was a decisive factor in achieving SNP‐embedded multishell hollow MOFs. In addition, alternating MOF layers with adjustable water stability enabled the step‐by‐step deterioration, resulting in the production of SNPs and the formation of multishell MOFs. In this process, we found that the π‐backbonding between organic ligands and dinuclear SNPs could stabilize SNPs in the MOFs. Also, the π‐backbonding and hollow structure led to enhanced electrical conductivity and minimized transport resistance. Moreover, the increased multishell number of H‐ZIF‐8[*n*S] allowed the scalable mass loadings of SNPs. Hence, these features of SNP‐embedded multishell hollow MOFs resulted in high capacities and low overpotentials in Li–O_2_ batteries. Consequently, this work provides a new method to produce and stabilize SNPs from alternating MOF layers so that it could be expanded to fabricate SNPs within various other types of frameworks. Additionally, the hopping charge transfer between SNPs stabilized by π‐backbondings presents new possibilities to introduce electrical conduction in MOFs, thereby enabling to exploit the numerous advantages of MOFs likely to be applicable in a broad range of future technologies such as solar cells and sensors systems in addition to energy storage and conversion systems.

## Experimental Section

##### Materials

Cobalt(II) nitrate hexahydrate (Co(NO_3_)_2_·6H_2_O, 98+%), zinc(II) nitrate hexahydrate (Zn(NO_3_)_2_·6H_2_O, 98+%), 2‐methyleimidazole (2‐mim, 99%), polyvinylpyrrolidone (PVP, mol. wt 10 000), EG (99.8%, anhydrous), cobalt(II) hydroxide (95%), methanol (99.9+%), ethanol (99.5+%), poly(tetrafluoroethylene) (PTFE, 1 µm), lithium perchlorate (LiClO4, 99.99+%), and tetraethylene glycol dimethyl ether (TEGDME, 99+%) were purchased from Sigma‐Aldrich. Deionized (d.i.) water was obtained from a purifying system using UV treatment. A carbon fiber paper (CFP, T090‐20%) was purchased from Toray Co., Ltd. Ketjen Black (KB, EC 600JD) was purchased from Lion Specialty Chemicals Co., Ltd.

##### Preparation of ML‐ZIFs[*n*L] with up to Ten Layers

First, precursor solutions of 95 × 10^−3^ m Co(NO_3_)_2_ in d.i. water and 800 × 10^−3^ m 2‐mim, 30 × 10^−3^ m Zn(NO_3_)_2_, and 30 × 10^−3^ m 2‐mim in methanol were prepared. PVP (5 g) was mixed with 200 mL of methanol. All solutions were sonicated for 30 min and cooled to room temperature. Then, to prepare the ZIF‐67 seeds, 3.3 mL of the 800 × 10^−3^ m 2‐mim solution and 3.9 mL of the PVP solution were transferred into a 50 mL conical tube and then mixed with 1.5 mL of the 95 × 10^−3^ m Co(NO_3_)_2_ solution. The mixture immediately turned purple and was kept for 1 h at room temperature. Subsequently, the purple solution was centrifuged at 8500 rpm for 10 min. To prepare ML‐ZIFs[2L], 7.5 mL of the 30 × 10^−3^ m 2‐mim solution was added to the collected purple powder in a conical tube. The mixture was sonicated for 10 min and then mixed with 7.5 mL of the 30 × 10^−3^ m Zn(NO_3_)_2_ solution. After being kept for 3 h at room temperature, the precipitate was collected by centrifugation at 600 rpm for 10 min and then washed with methanol three times. A new conical tube was used for every step. Finally, ML‐ZIFs[*n*L] was prepared via the following steps. Initially, 3 mL of the 800 × 10^−3^ m 2‐mim solution (4 mL for the subsequent layer‐stacking processes), freshly prepared in methanol, was added to the ML‐ZIFs[2L, 4L, 6L, or 8L] powder in a conical tube. The mixture was sonicated for 5 min to achieve dispersion, and then 0.75 mL of the 95 × 10^−3^ m Co(NO_3_)_2_ solution was added. After 30 min, the solution was centrifuged at 8500 rpm for 10 min, 12.5 mL of the 30 × 10^−3^ m 2‐mim solution was added, and the mixture was sonicated for 5 min. Then, 12.5 mL of the 30 × 10^−3^ m Zn(NO_3_)_2_ solution was added and the mixture was kept for 3 h. The above process was repeated every time two new layers of ML‐ZIFs[2L] were stacked.

##### Synthesis of H‐ZIF‐8[*n*S]

First, 760 mL of pure EG (stored at −15 °C in the refrigerator) was transferred into a 1 L HDPE Nalgene bottle. Then, 40 mL of d.i. water was added and the solution was sonicated for 10 min. Subsequently, 100 mg of as‐synthesized ML‐ZIFs[*n*L] was mixed with the EG–water solution. The mixture was sonicated for 6 h at 5 °C until a transparent purple solution was obtained. The solution was transferred into a 1 L round‐bottom flask and stirred at room temperature for 6 h, and the resulting mixture was filtered using a membrane filter. The powder on the membrane was collected by sonication in methanol. Following centrifugation at 6000 rpm for 10 min, the powder was washed with methanol three times. Finally, the collected powder was dried in a vacuum oven at 60 °C for 24 h.

##### Characterizations

The powder XRD patterns were obtained using a SmartLab X‐ray diffractometer (Rigaku, Japan) with Cu‐Kα radiation at 1200 W (40 kV, 30 mA). The TEM and SEM images and videos were obtained using a JEM‐ARM200F instrument (JEOL, Japan) operated at 200 kV and a JEM‐7600F instrument (JEOL, Japan) operated at 15 kV, respectively. ICP‐OES data were obtained using an ICP‐MS 7700S instrument (Agilent, USA). The XPS spectra were collected using a Thermo VG Scientific K‐alpha spectrometer (Thermo Scientific, USA) using Al‐Kα radiation at 350 W (3 mA). The Fourier transform infrared spectra were obtained using an FT/IR‐6100FV spectrometer (JASCO, USA) via the attenuated total reflectance technique. The X‐ray absorption spectroscopy (XAS) measurements, including the XANES and EXAFS analyses, were conducted at the multipole‐wiggler 10C beamline at the Pohang Accelerator Laboratory (PAL, Republic of Korea). The NEXAFS measurements were performed at the 10D beamline, where the synchrotron radiation was monochromatized using a Si(111) double crystal monochromator and the incident beam was detuned at the appropriate rate for harmonic rejection. All the NMR experiments were performed on a Bruker Avance 400 MHz spectrometer, equipped with a 5 mm BBFO probe with z‐gradients. The Topspin software was used for spectrometer control, and all the pulse sequences were used without modification.

##### Electrochemical Performance of Li–O_2_ Batteries

The electrochemical performance measurements were conducted using a WBCS3000L32 instrument (Won‐A‐Tech, Republic of Korea) as a galvanostatic cycle tester. The ink used for the working electrode was prepared by mixing H‐ZIF‐8, KB, and PTFE in a weight ratio of 4:5:1 with ethanol. The ink was coated to a thickness of 400 µm on CFP, which was cut into circular pieces with a 10 mm diameter. The electrode was placed in a vacuum oven and dried at 60 °C for 12 h. After weighing, the electrode was rinsed with acetone several times and vacuum dried in a glove box at 60 °C for 12 h. For the Li–O_2_ battery test, a Swagelok‐type cell (Wellcos, Republic of Korea) was assembled with a Li foil anode, a glass fiber filter (Whatman, GF/D) as a separator, 1 m LiClO_4_ in TEGDME as an electrolyte, and the as‐prepared working electrode. Water in TEGDME was removed using molecular sieves (3 Å) for 10 days. The assembled cell was purged with a 25 cm^3^ min^−1^ flow of pure oxygen for 3 h at 1.1 bar. The oxygen purging pressure was maintained during the Li–O_2_ battery test and confirmed at an outlet valve using an MP112 micromanometer (KIMO, France). The EIS measurements were conducted with a PGSTAT302N potentiostat/galvanostat (Metrohm Autolab B.V., Netherlands) using an FRA32MBA module.

## Conflict of Interest

The authors declare no conflict of interest.

## Supporting information

Supporting Information

Supplemental Movie 1
