# Supplementary material for: Autogenous Production and Stabilization of Highly Loaded Sub‐Nanometric Particles within Multishell Hollow Metal–Organic Frameworks and Their Utilization for High Performance in Li–O2 Batteries
Source: Adv Sci (Weinh). 2020 Mar 16;7(9):2000283. doi: 10.1002/advs.202000283 (PMC7201254; doi:10.1002/advs.202000283)
Supplement: Supplementary file 1 — Supporting Information [file ADVS-7-2000283-s001.pdf]

Copyright WILEY-VCH Verlag GmbH & Co. KGaA, 69469 Weinheim, Germany, 2020.

ADVANCED SCIENCE
Open Access

Supporting Information

for *Adv. Sci.,* DOI: 10.1002/advs.202000283

Autogenous Production and Stabilization of Highly Loaded Sub-Nanometric Particles within Multishell Hollow Metal–Organic Frameworks and Their Utilization for High Performance in Li–O_2 Batteries

Won Ho Choi, Byeong Cheul Moon, Dong Gyu Park, Jae Won Choi, Keon-Han Kim, Jae-Sun Shin, Min Gyu Kim, Kyung Min Choi, and Jeung Ku Kang**

Copyright WILEY-VCH Verlag GmbH & Co. KGaA, 69469 Weinheim, Germany, 2020.

Supporting Information

Autogenous production and stabilization of highly loaded subnanometric particles within multishell hollow metal-organic frameworks and their utilization for high performance in Li-O_2 batteries

Won Ho Choi, Byeong Cheul Moon, Dong Gyu Park, Jae Won Choi, Keon-Han Kim, Jae-Sun Shin, Min Gyu Kim, Kyung Min Choi, and Jeung Ku Kang**

W. H. Choi, B. C. Moon, D. G. Park, J. W. Choi, K. -H. Kim, Prof. J. K. Kang
Department of Materials Science and Engineering and NanoCentury KAIST Institute
Korea Advanced Institute of Science and Technology (KAIST)
291 Daehak-ro, Yuseong-gu, Daejeon 34141, Republic of Korea
*Email: jeungku@kaist.ac.kr

Dr. J. -S. Shin
Department of Chemistry
Korea Advanced Institute of Science and Technology (KAIST)
291 Daehak-ro, Yuseong-gu, Daejeon 34141, Republic of Korea

Dr. M. G. Kim
Pohang Accelerator Laboratory (PAL)
Korea Pohang University of Science and Technology
77 Cheongam-ro, Namg-gu, Pohang 37673, Republic of Korea

Prof. K. M. Choi
Department of Chemical and Biological Engineering
Sookmyung Women's University
Cheongpa-ro 47-gil 100, Yongsan-gu, Seoul 04310, Republic of Korea
*Email: kmchoi@sookmyung.ac.kr

Prof. J. K. Kang
Graduate School of Energy, Environment, Water, and Sustainability (EEWS)
Korea Advanced Institute of Science and Technology (KAIST)
291 Daehak-ro, Yuseong-gu, Daejeon 34141, Republic of Korea

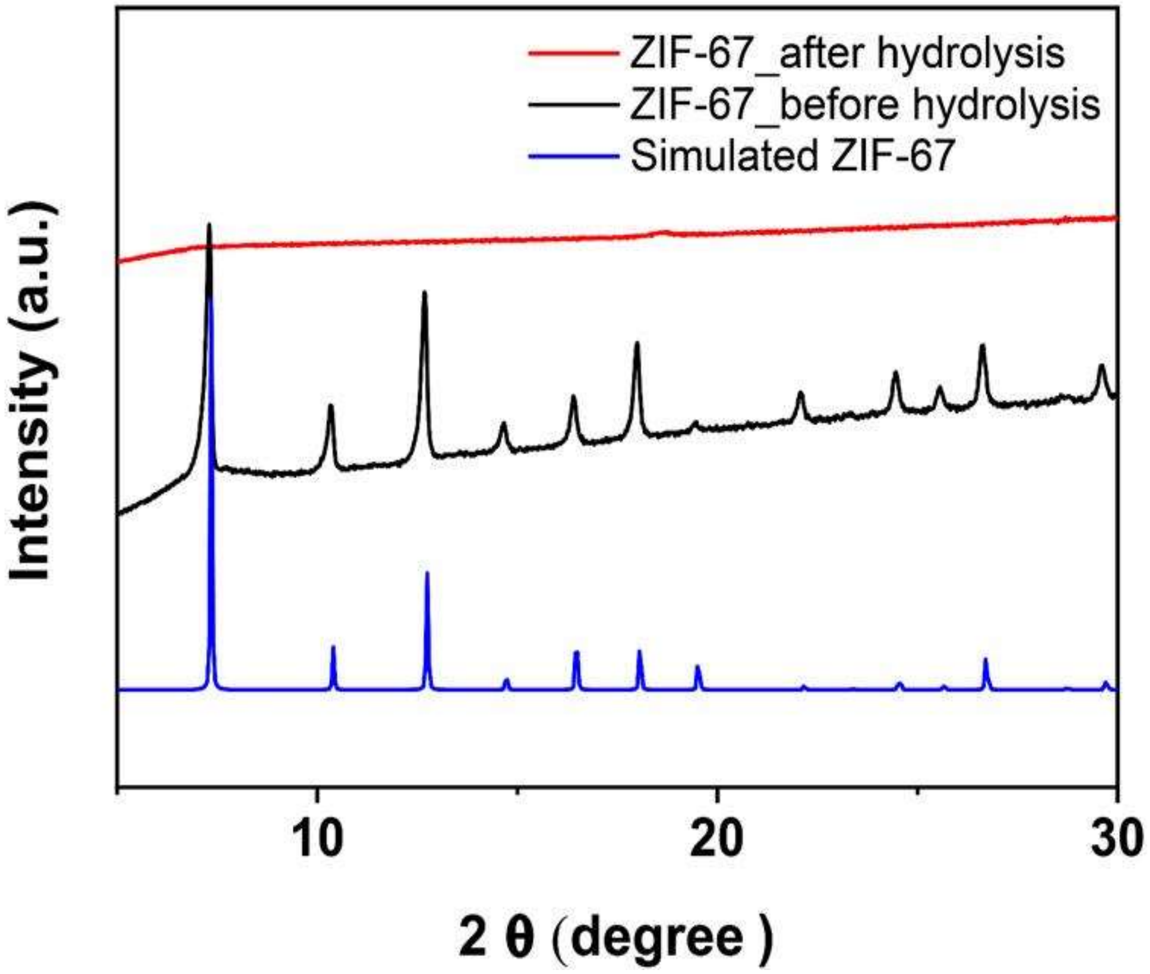

Figure S1. XRD patterns after hydrolysis with ZIF-67. The solution color changed from purple to light pink and dark green sequentially during hydrolysis at 318 K.

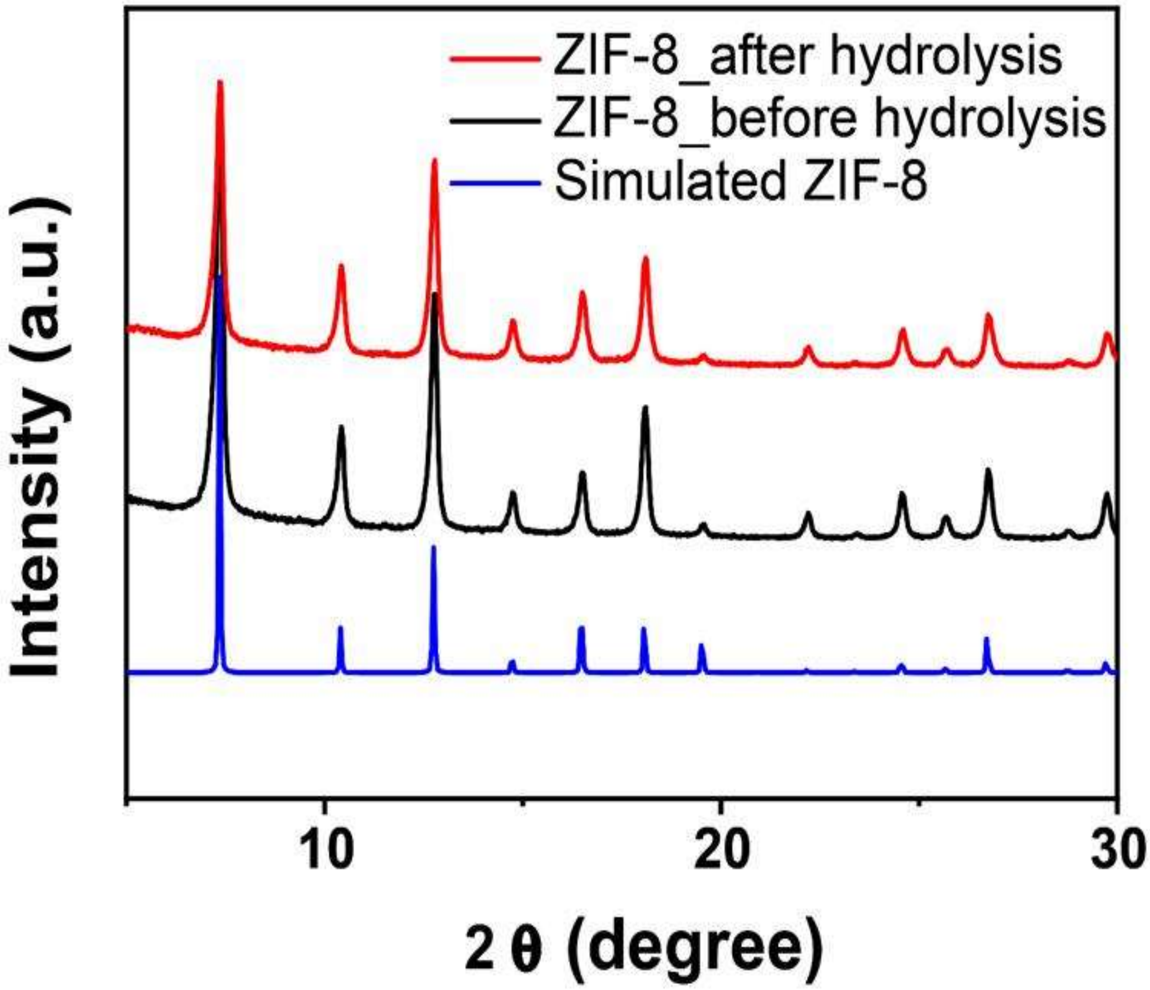

Figure S2. XRD patterns after hydrolysis with ZIF-8. The white solution did not change during hydrolysis at 318 K.

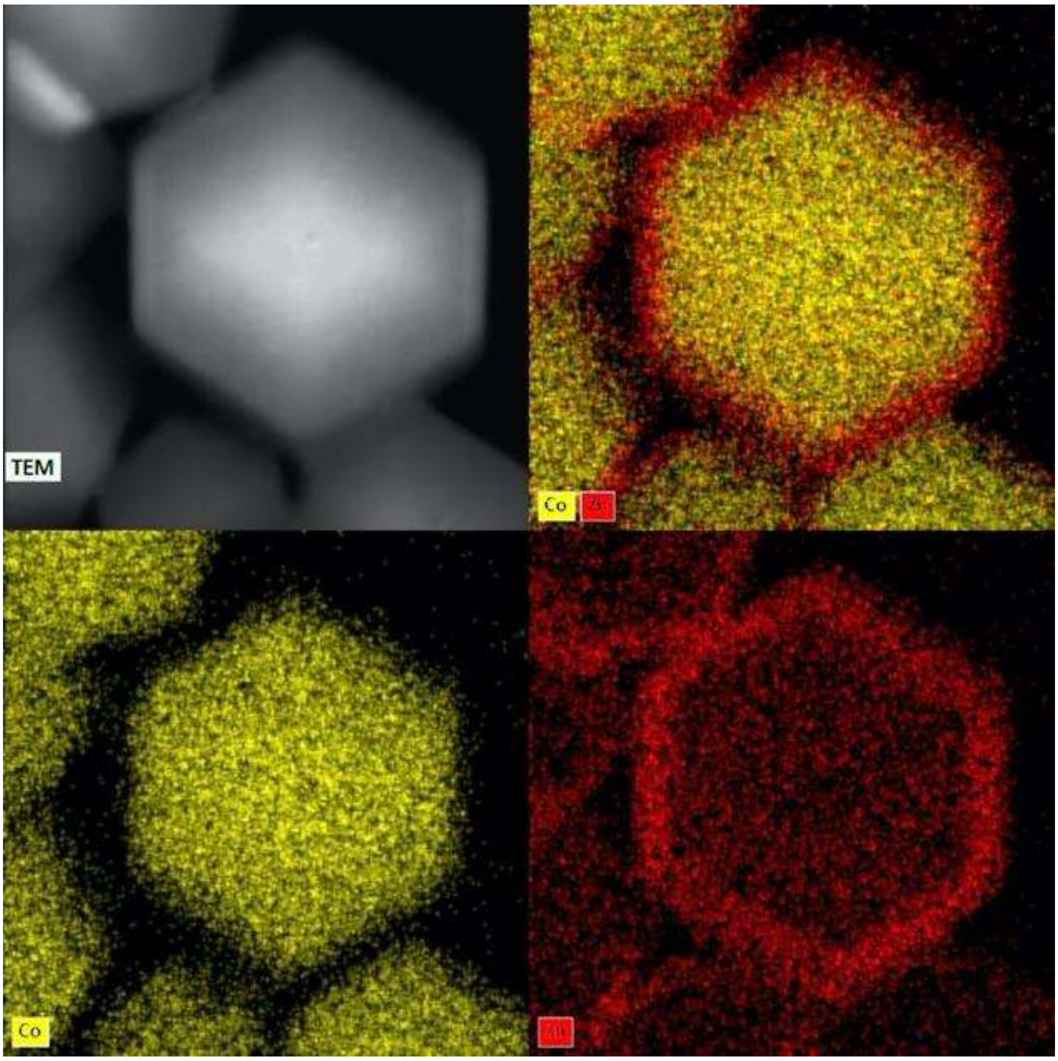

Figure S3. HADDF-STEM and EDS images of ML-ZIFs[2L]. The yellow and red represent cobalt(core) and zinc(shell), respectively.

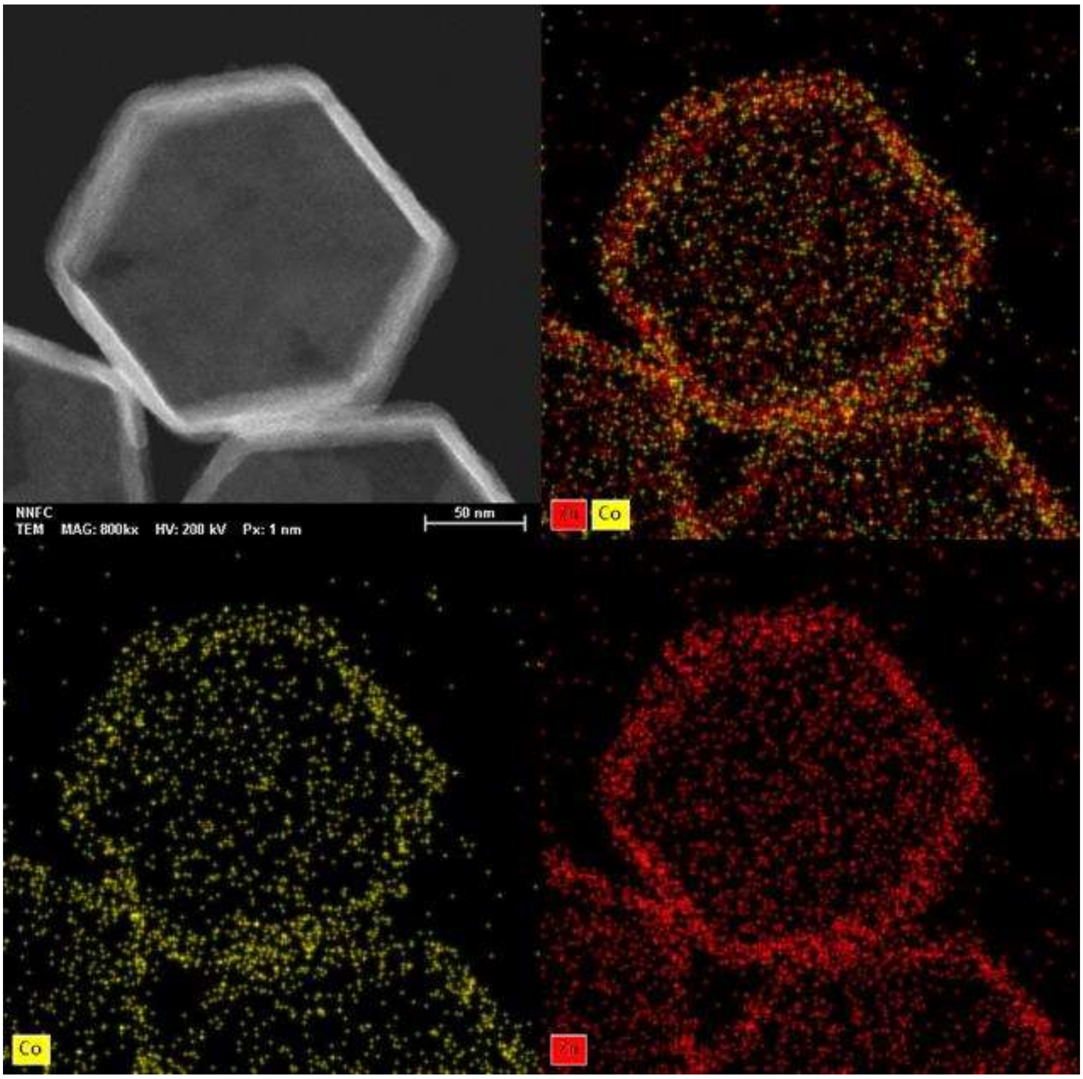

Figure S4. HADDF-STEM and EDS images of H-ZIF-8[1S]. The yellow and red represent cobalt(core) and zinc(shell), respectively.

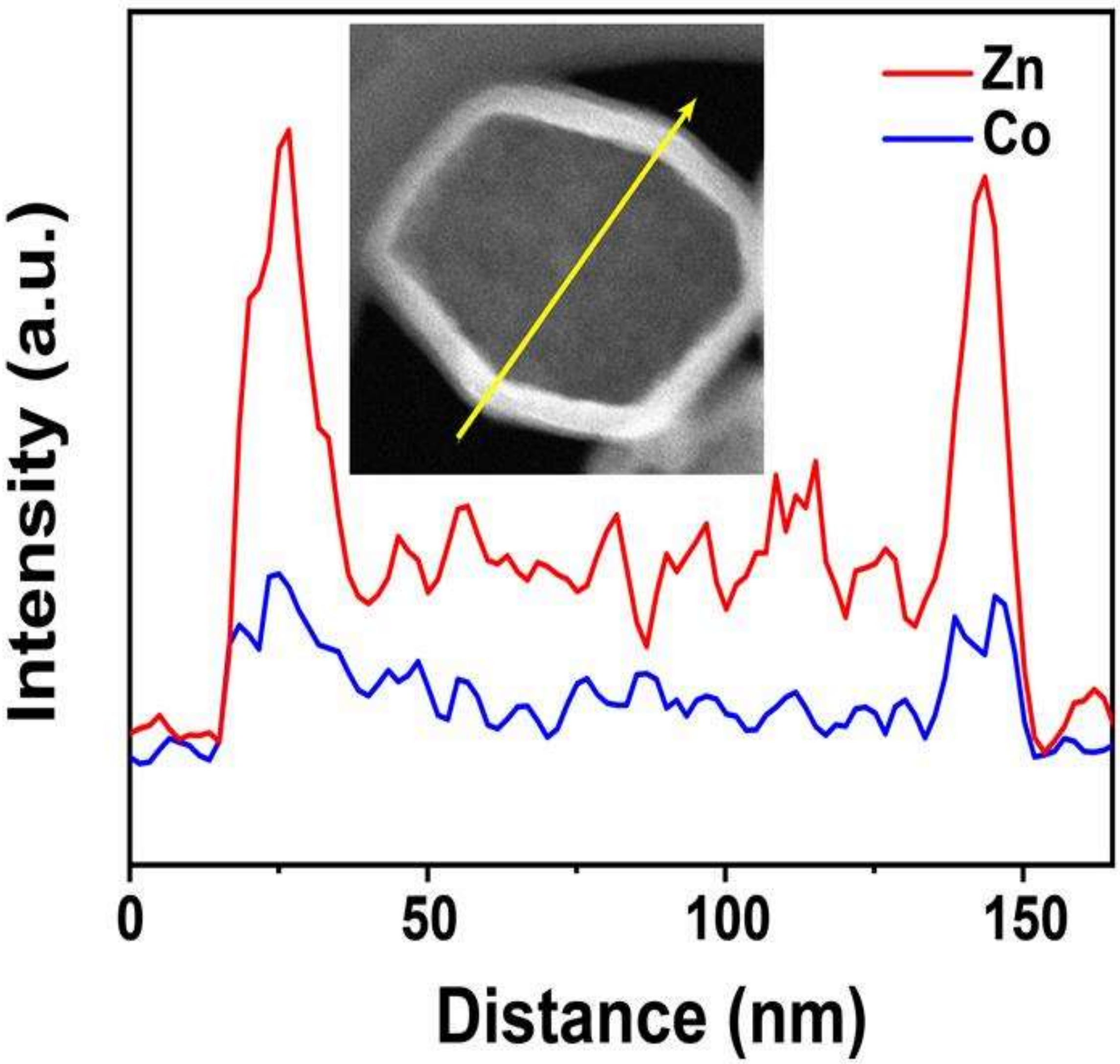

Figure S5. Line profiling graph of the HAADF-STEM image corresponding to H-ZIF-8[1S].

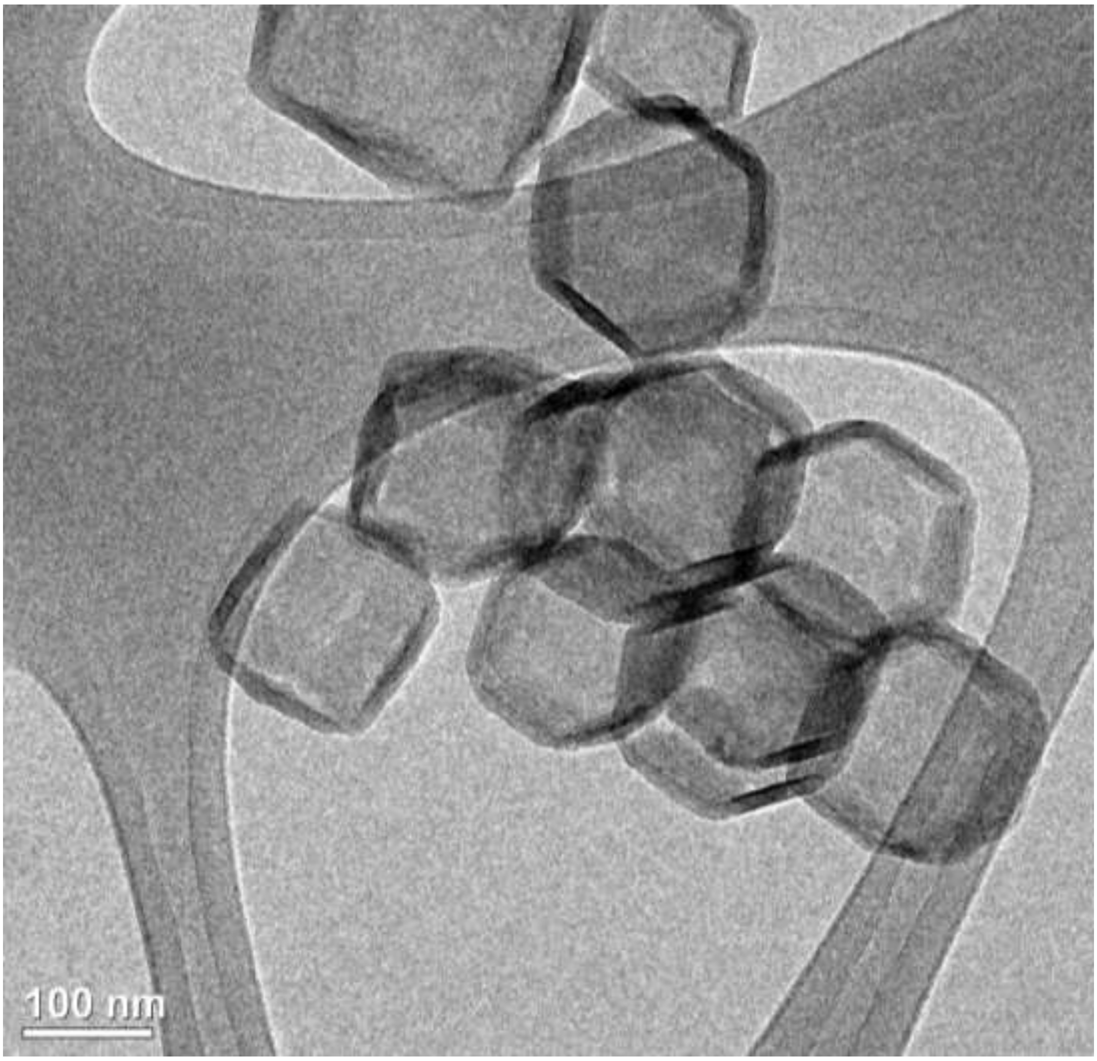

Figure S6. TEM image of H-ZIF-8[1S].

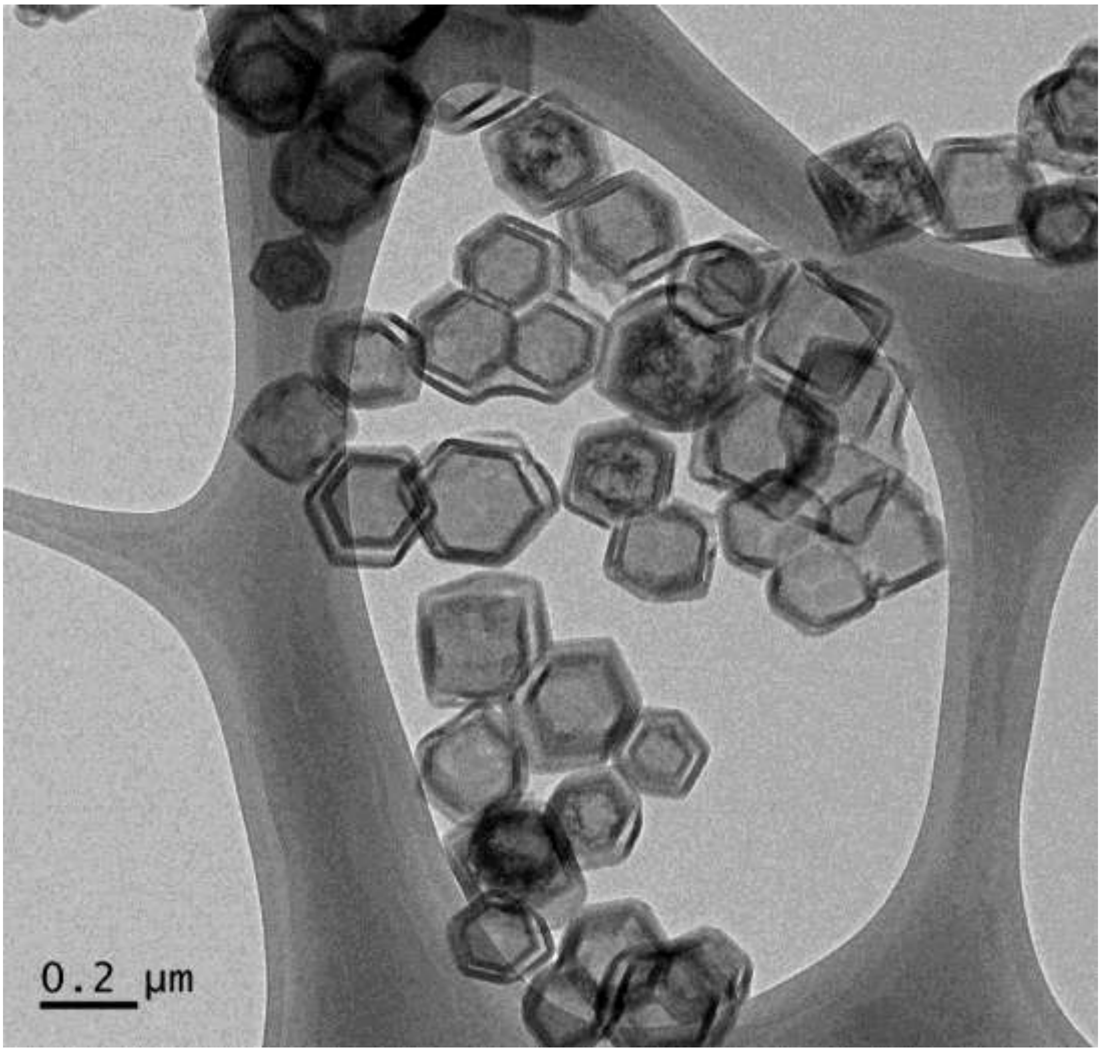

Figure S7. TEM image of H-ZIF-8[2S].

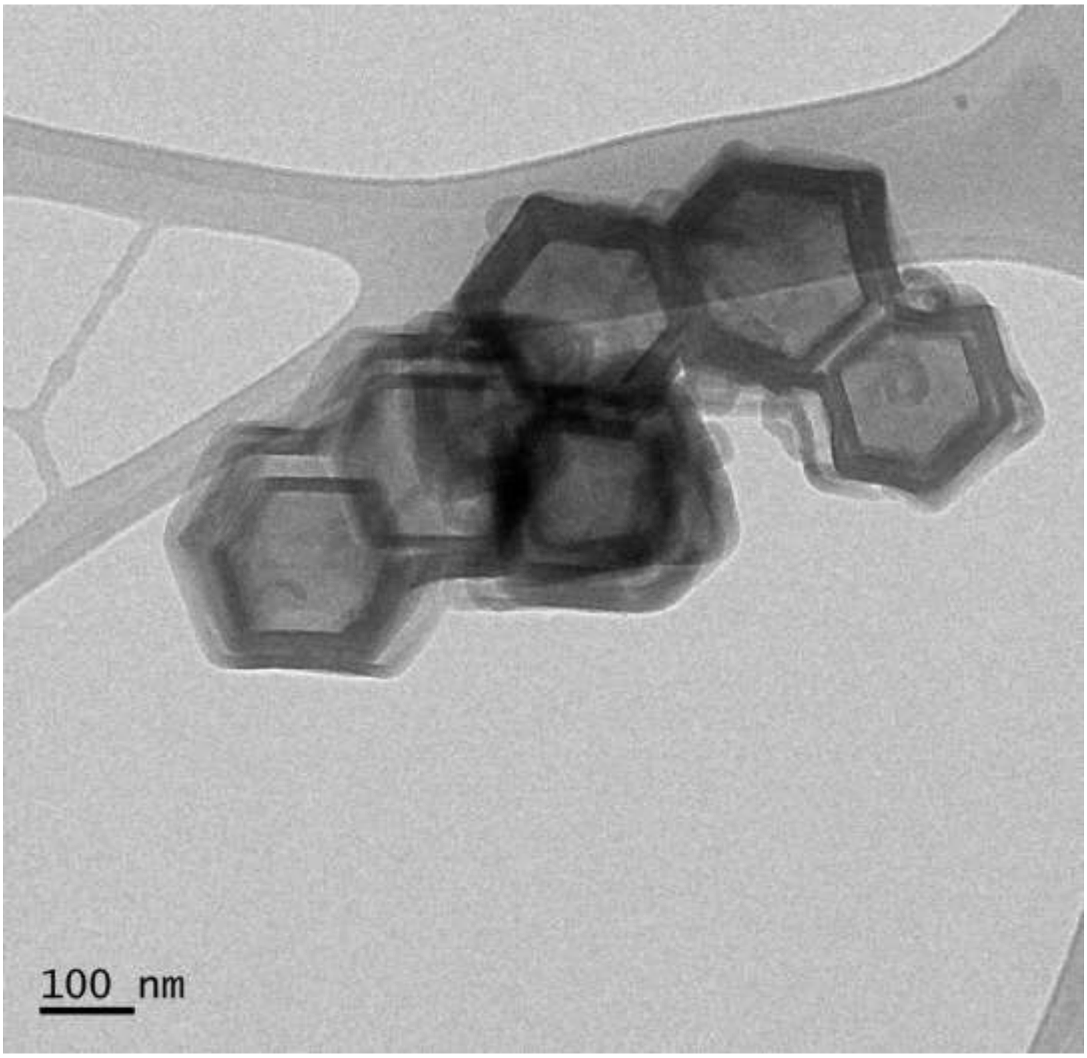

Figure S8. TEM image of H-ZIF-8[3S].

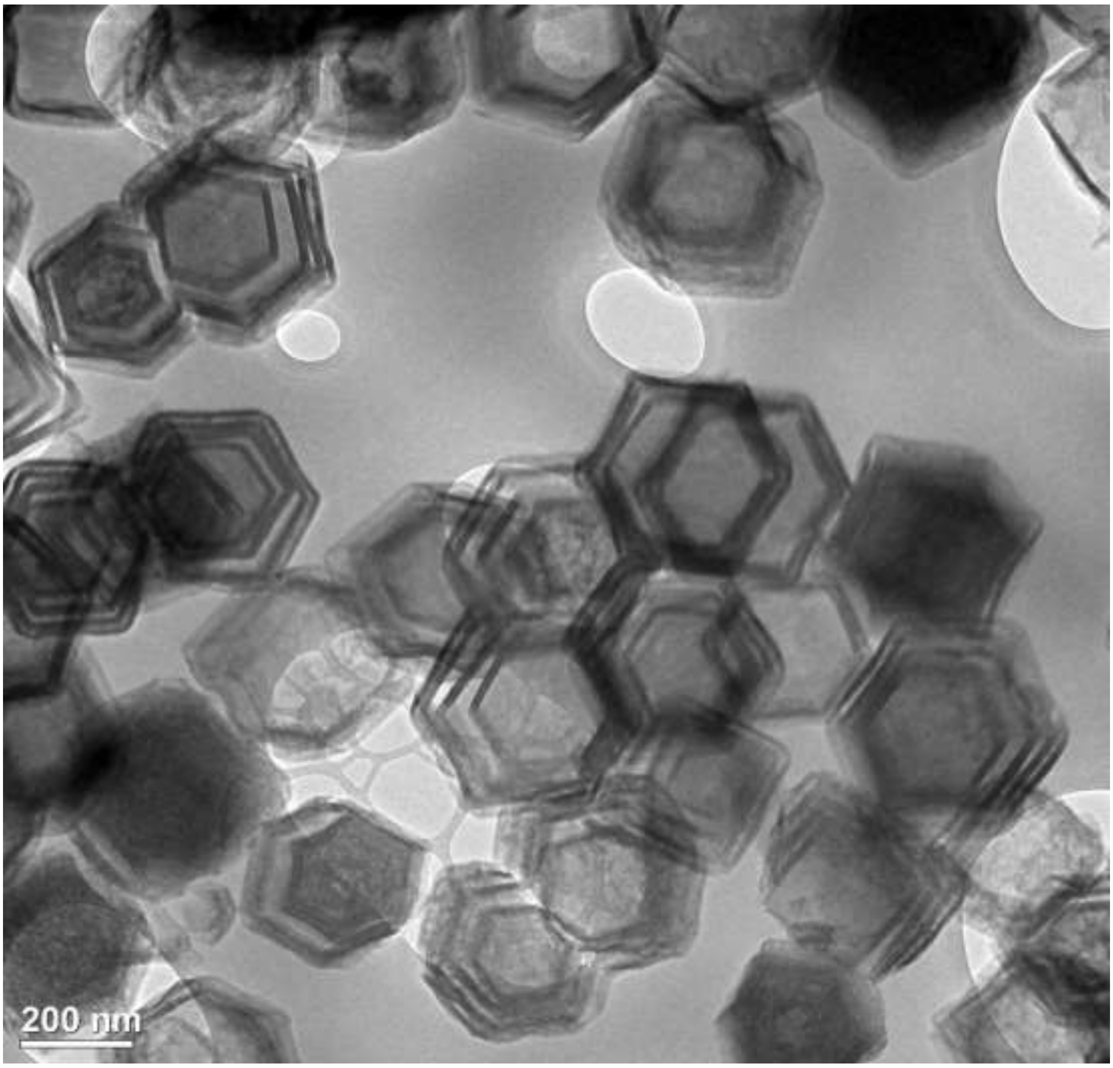

Figure S9. TEM image of H-ZIF-8[4S].

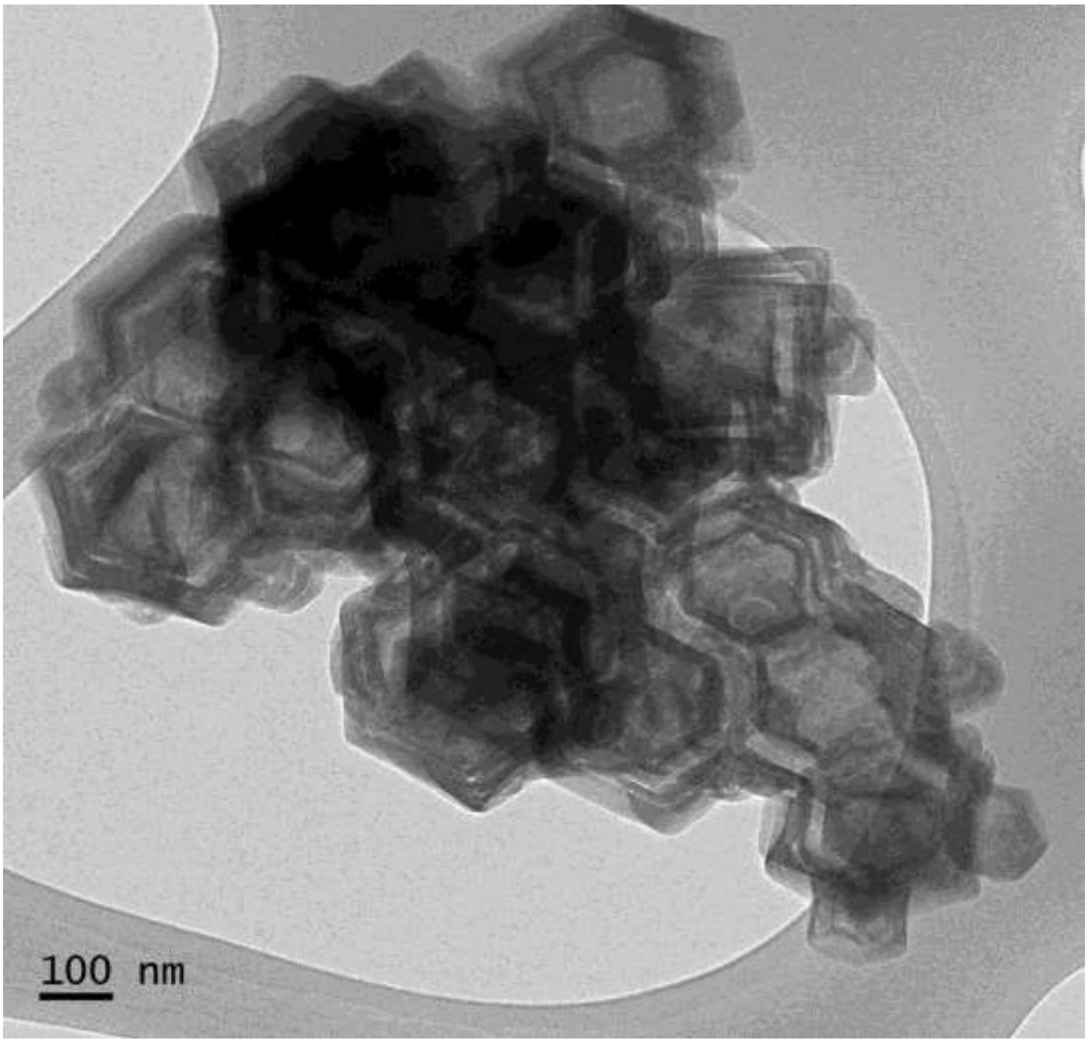

Figure S10. TEM image of H-ZIF-8[5S].

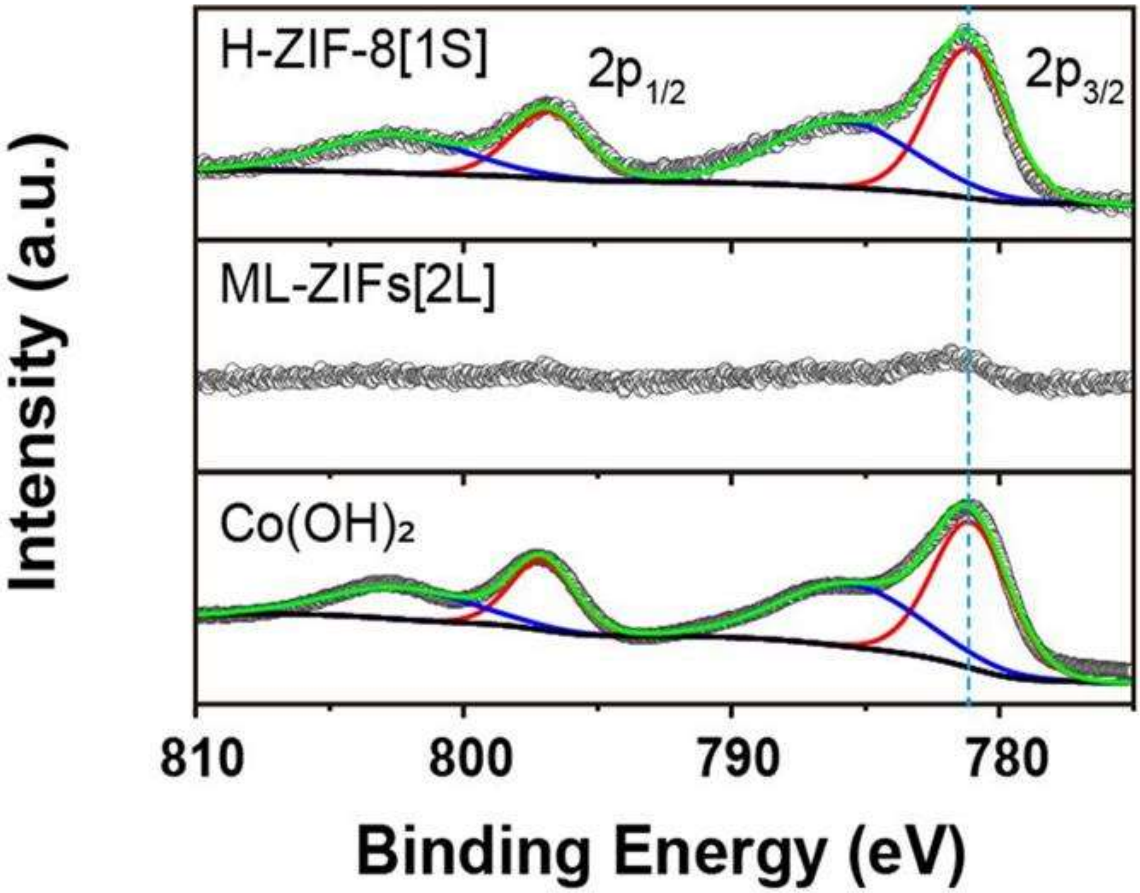

Figure S11. Co 2p XPS spectra of H-ZIF-8[1S], ML-ZIFs[2L] and ZIF-8. A peak of H-ZIF-8[1S] shows a migration and an oxidation state of the Co. All peaks are shifted by 285.4 eV for the sp^2 C=N bonding.

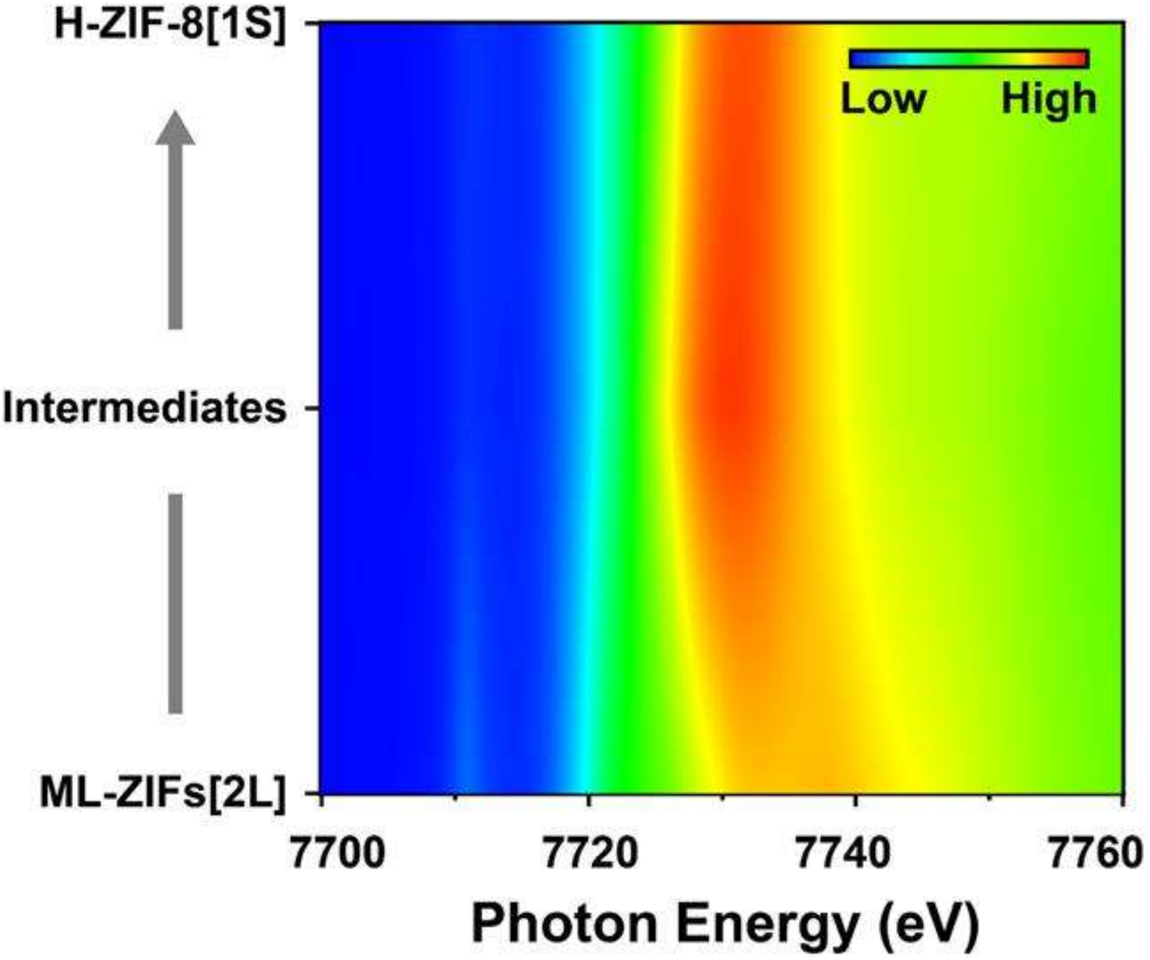

Figure S12. Two-dimensional contour map derived from XANES spectra.

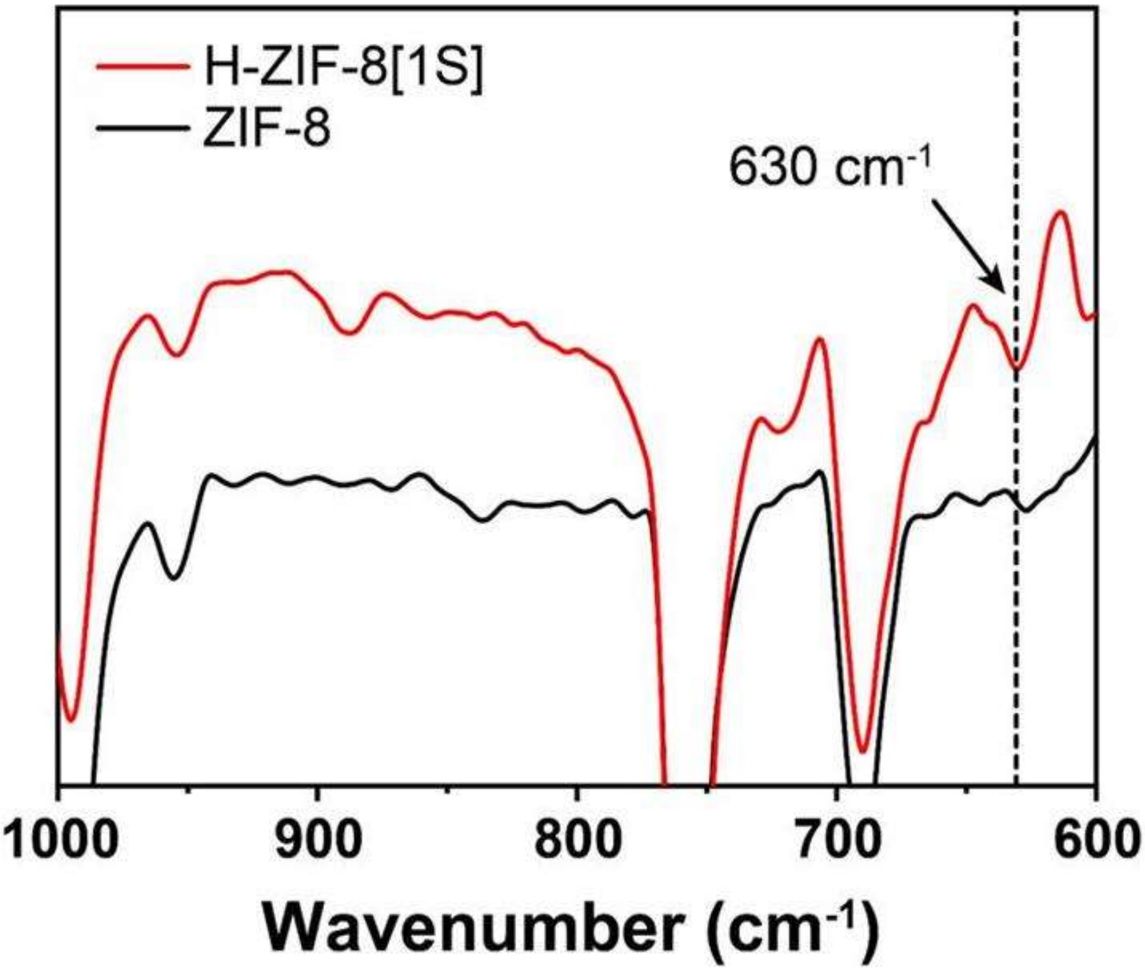

Figure S13. IR spectra of H-ZIF-8[1S] and ZIF-8. A peak at 630 cm^{-1} means the Co-O bending.

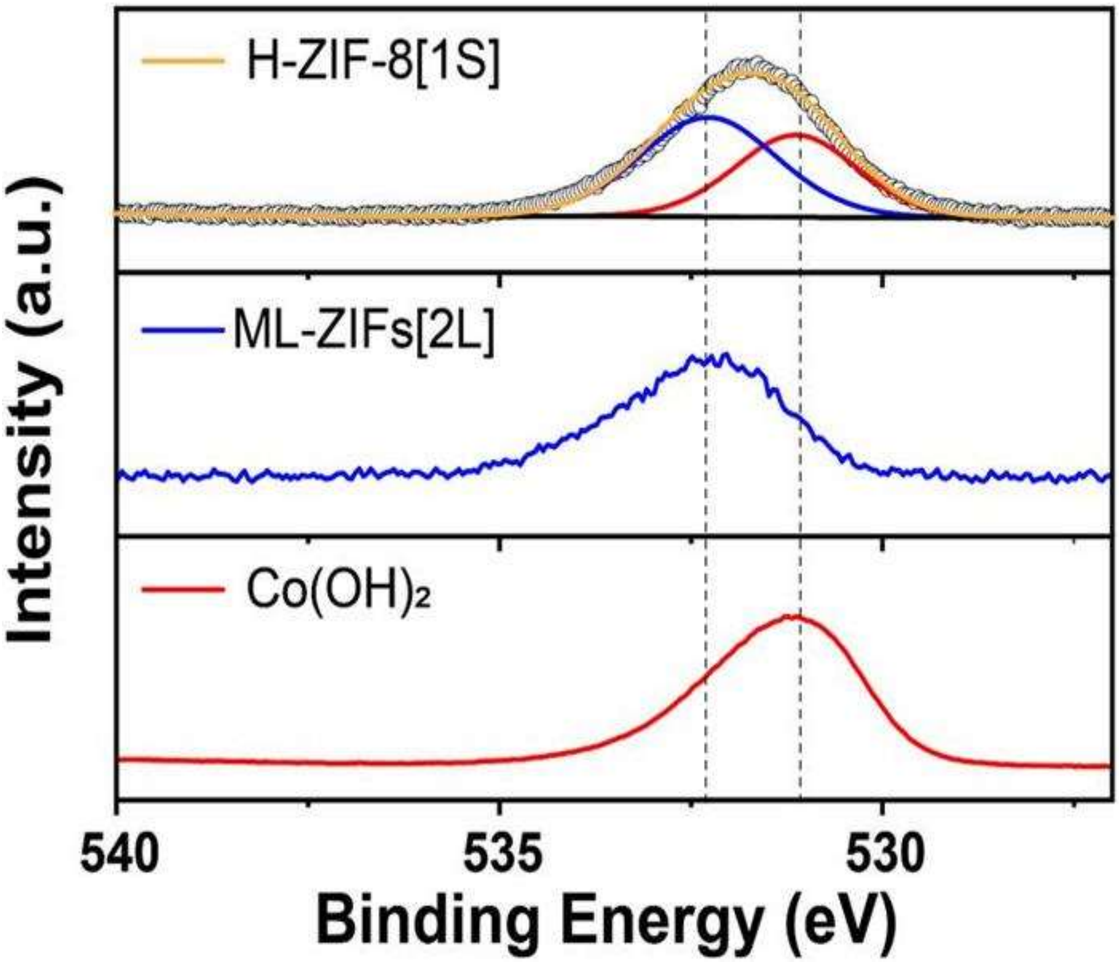

Figure S14. O 1s XPS spectra of H-ZIF-8[1S], ML-ZIFs[2L] and Co(OH)$_2$. A peak corresponding to ML-ZIFs[2L] at 532.2 eV means the adsorbed H_2O. Another peak corresponding to Co(OH)$_2$ at 531 eV means the presence of OH.

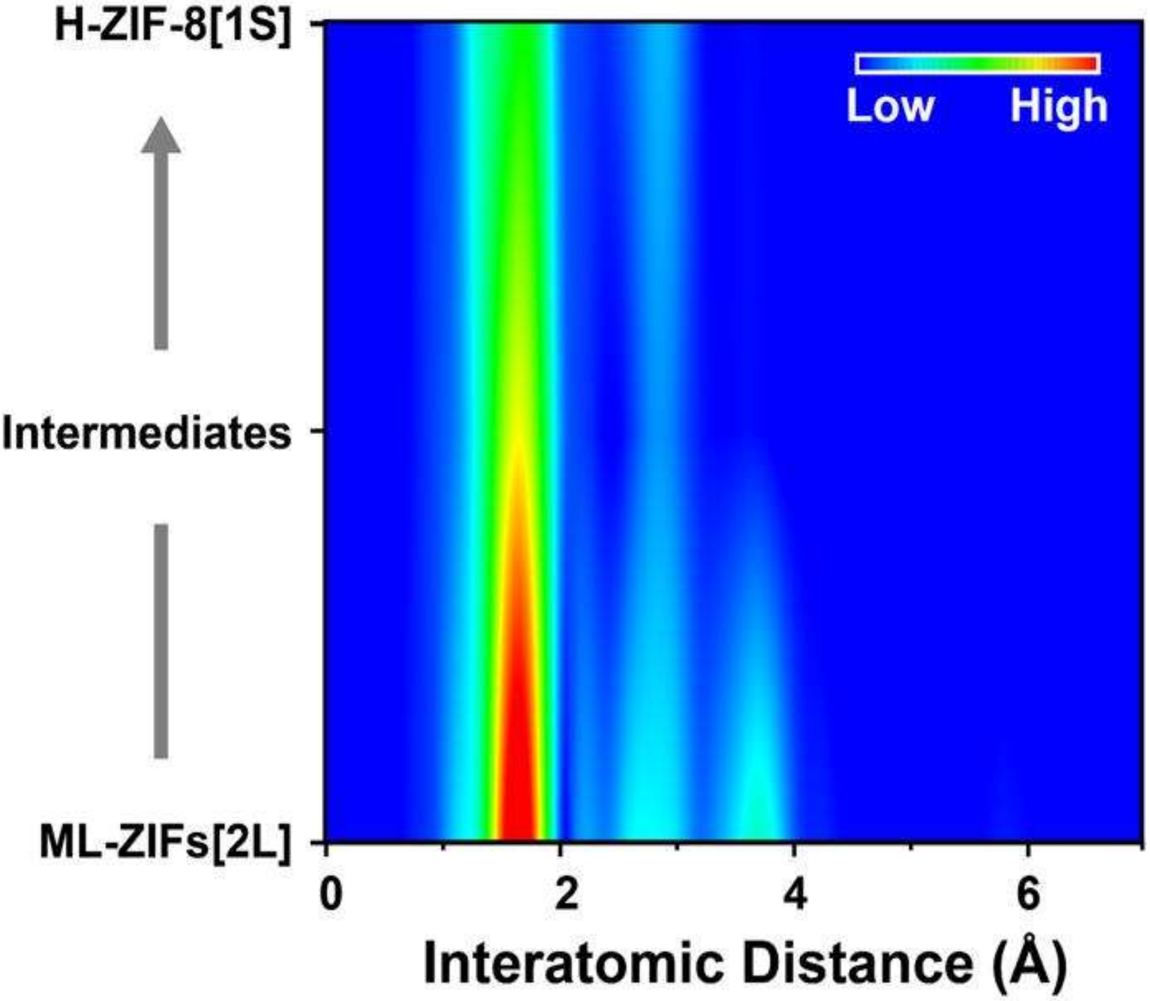

Figure S15. Two-dimensional contour map derived from EXAFS spectra

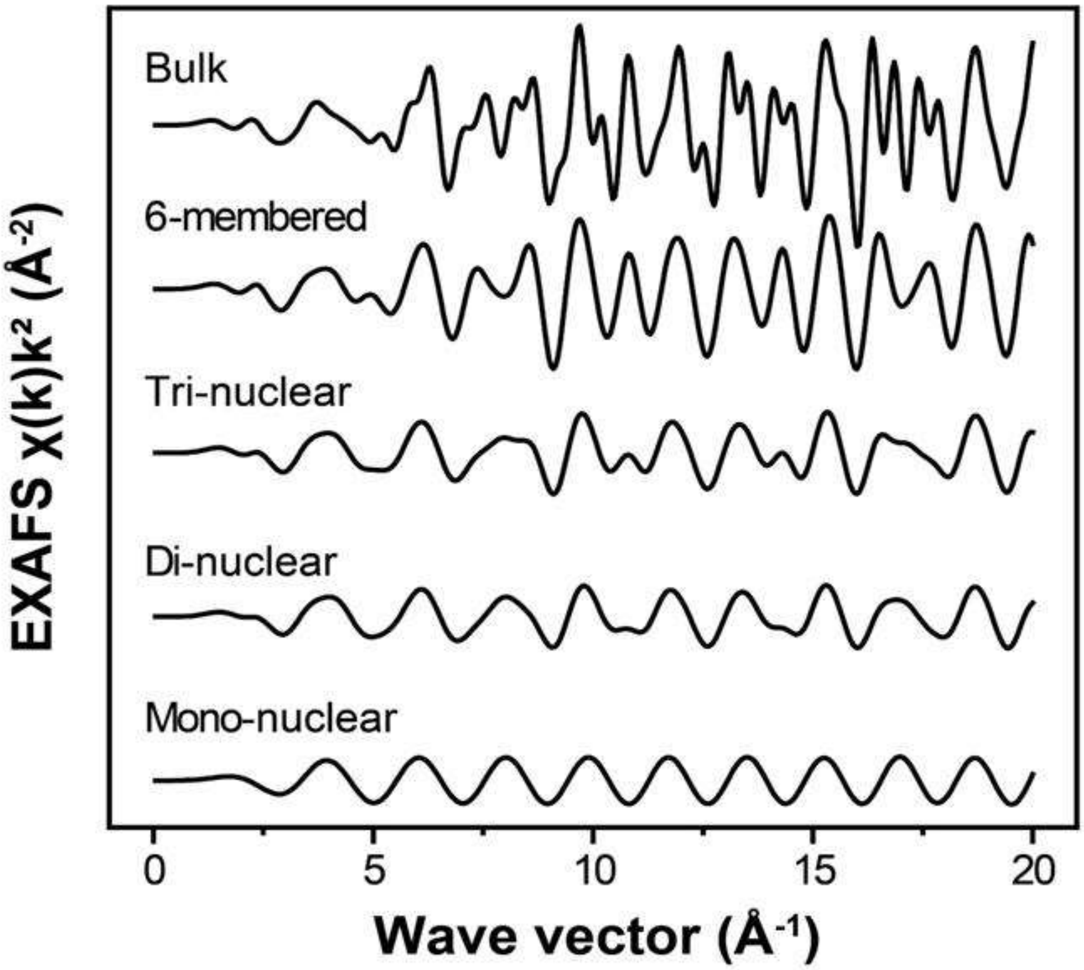

Figure S16. Co K-edge EXAFS $\chi(k)k^2$ simulation signals with $Co(OH)_2$ size.

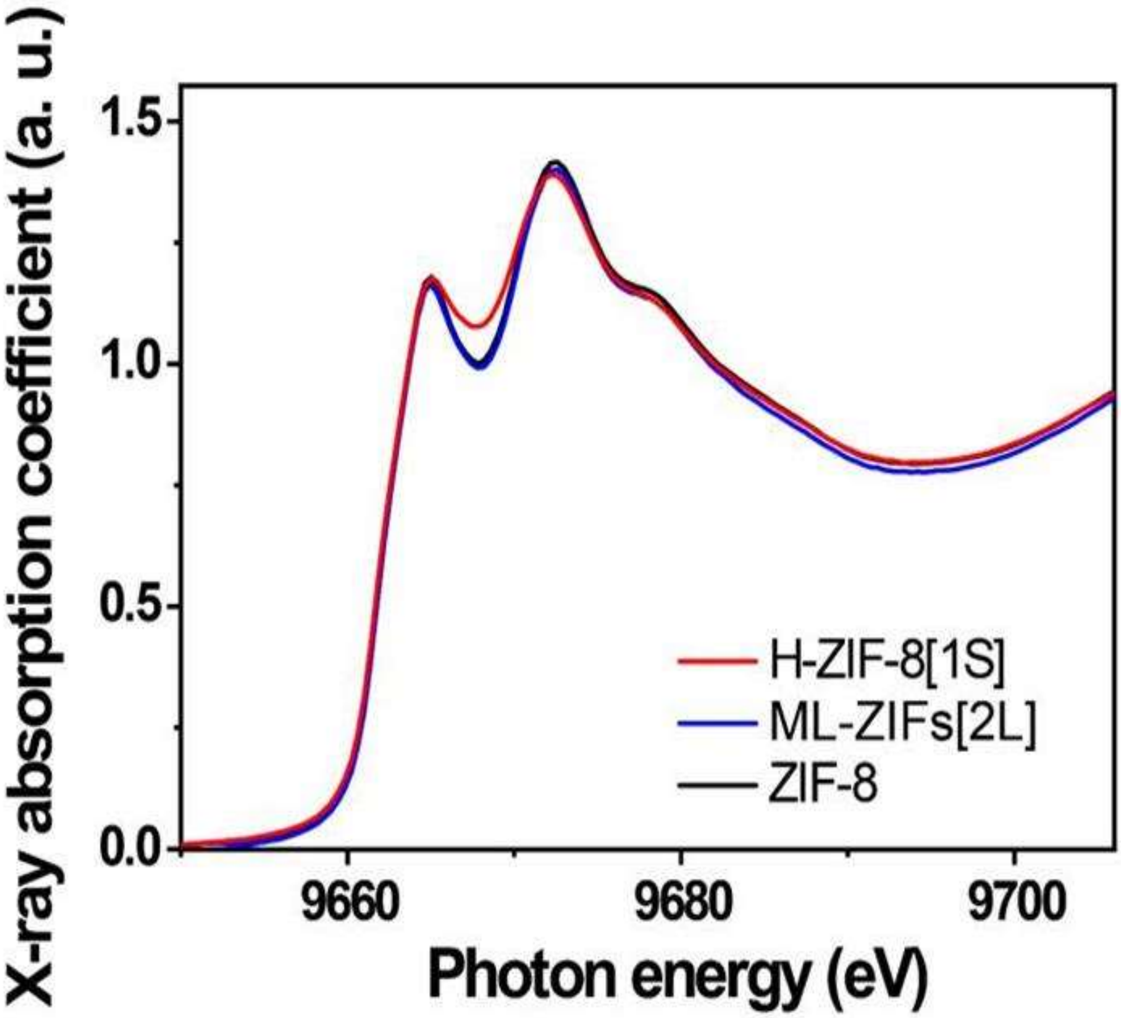

Figure S17. Zn K-edge XANES spectra of ZIF-8, ML-ZIFs, and H-ZIF-8.

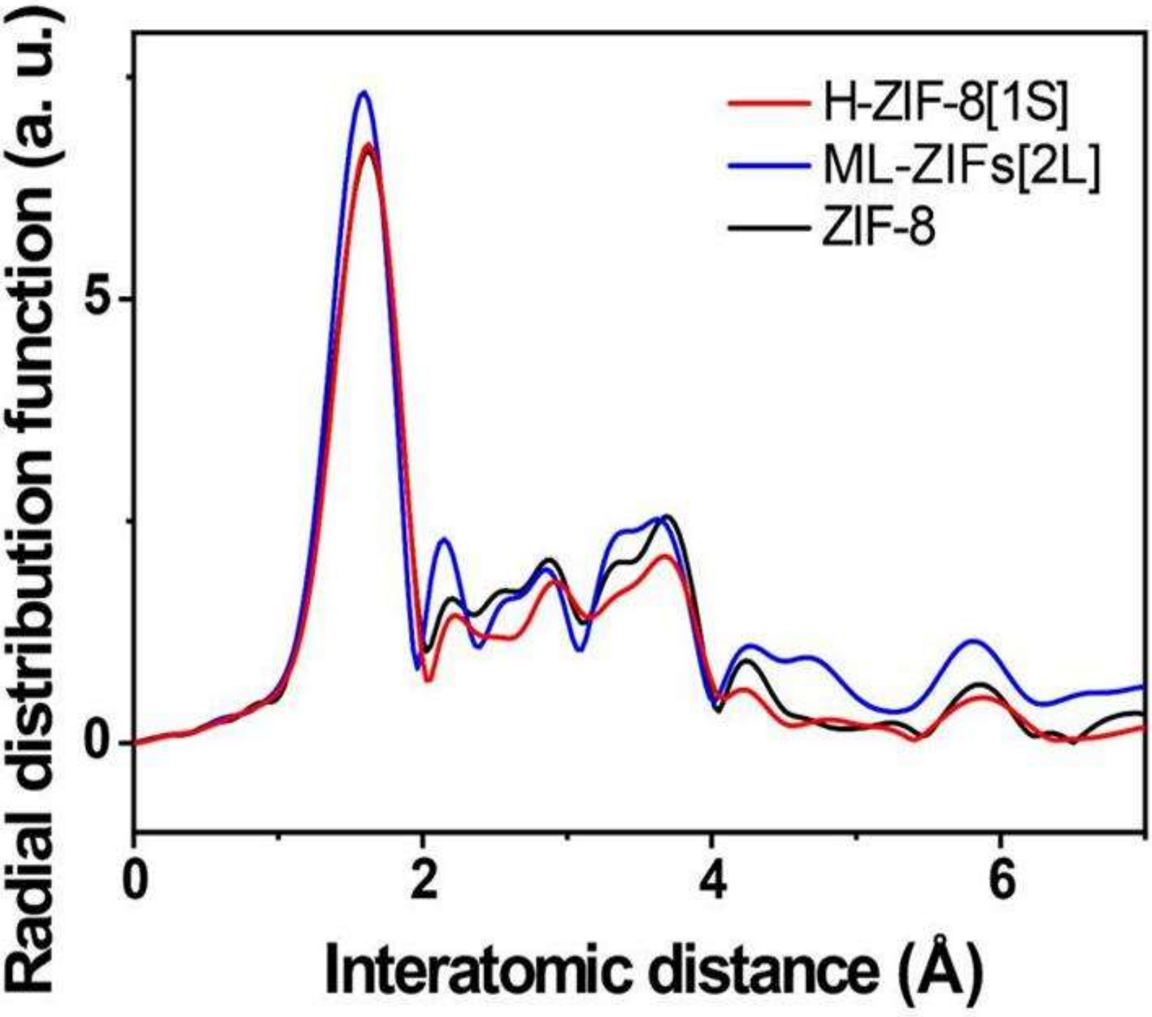

Figure S18. **Fourier transform of k^3-weighted Zn EXAFS spectra of ZIF-8, ML-ZIFs[2L], and H-ZIF-8[1S].**

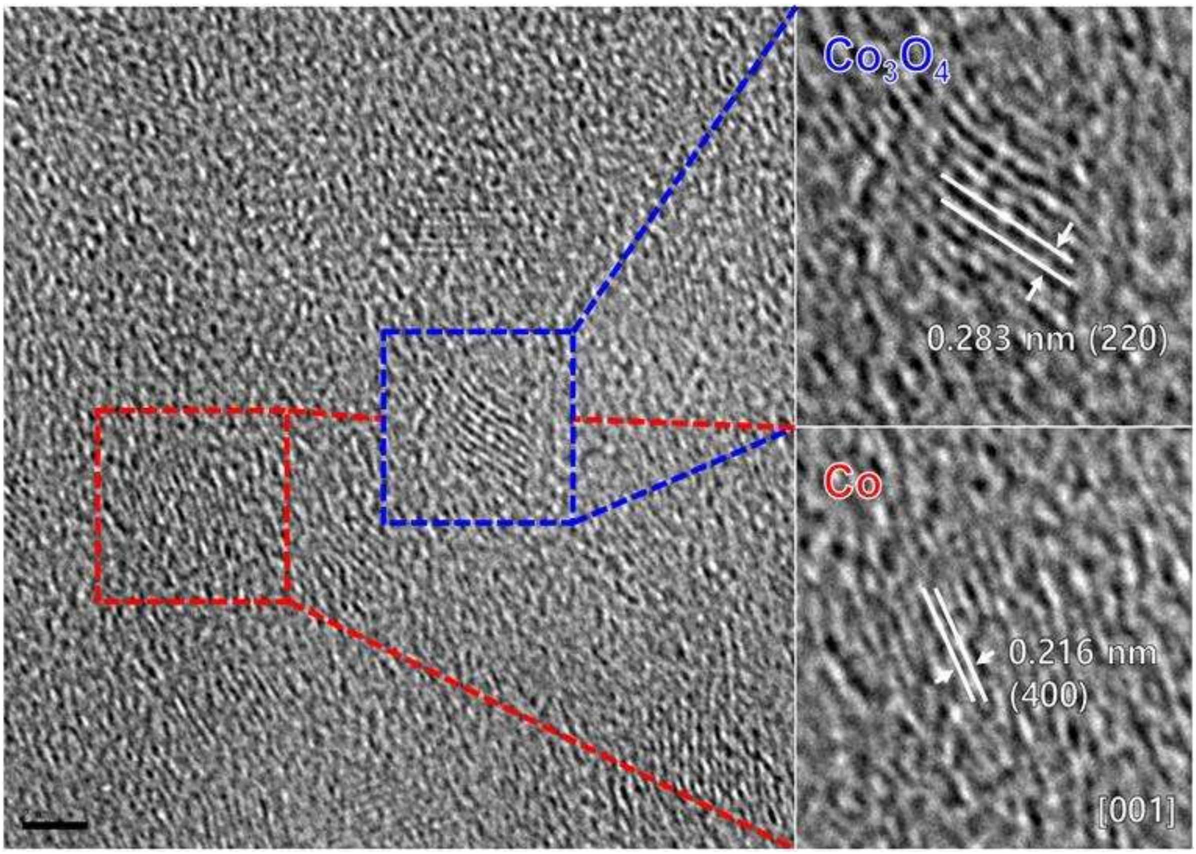

Figure S19. TEM image of H-ZIF-8[1S] showing the "disordered-to-crystalline" phenomenon upon exposure to a highly enhanced electron beam for 2 minutes. This corroborates the existence of SNPs inside the damaged multishell hollow MOFs where the scale bar is 1 nm.

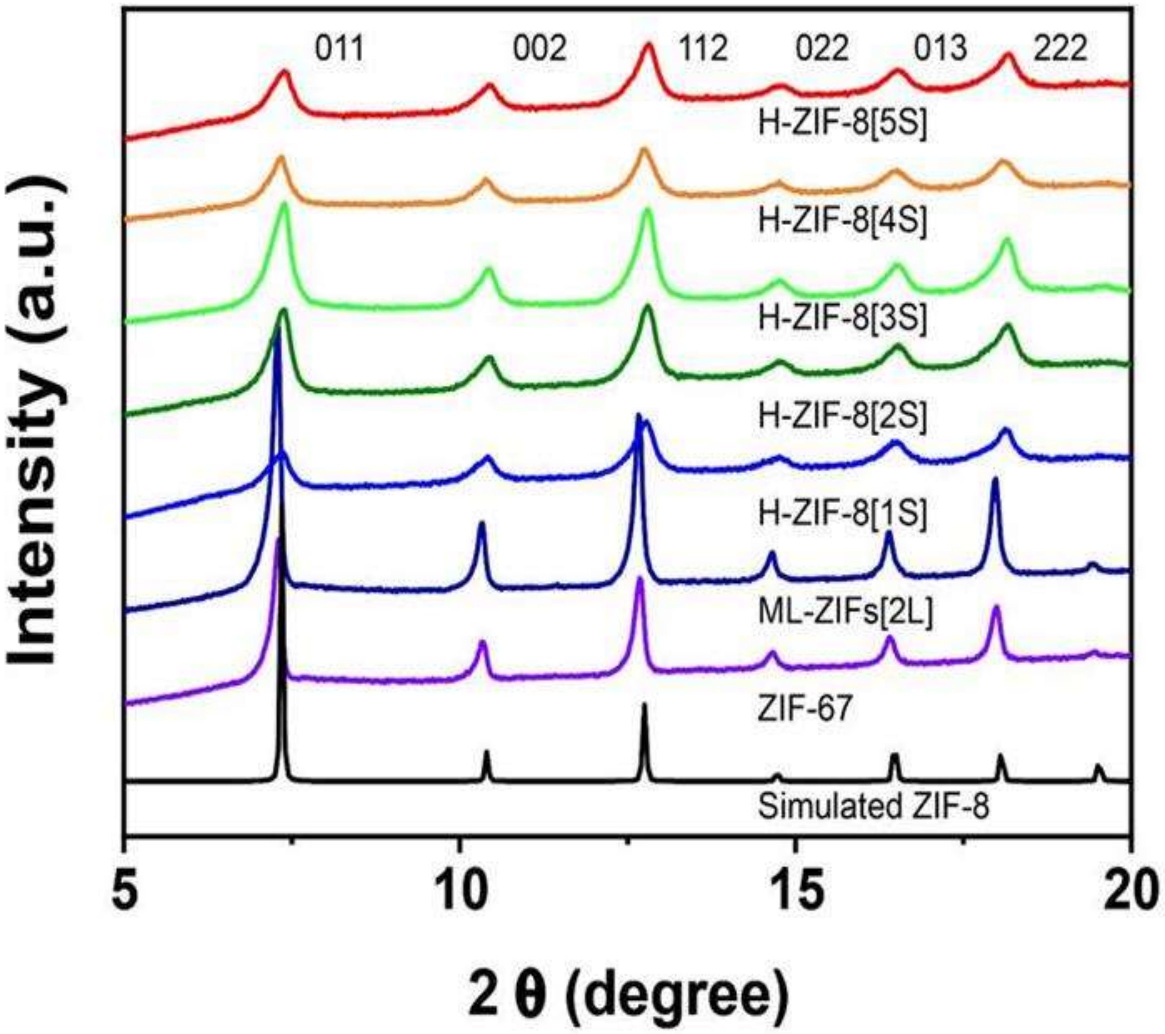

Figure S20. XRD patterns of H-ZIF-8[nS], ML-ZIFs[2L], ZIF-67 and simulated ZIF-8. The facet corresponding to each peak is displayed at the top. H-ZIF-8[5S]:red, H-ZIF-8[4S]:orange, H-ZIF-8[3S]:green, H-ZIF-8[2S]:dark green, H-ZIF-8[1S]: blue, ML-ZIFs[2L]: dark blue, ZIF-67:violet and simulated ZIF-8:black.

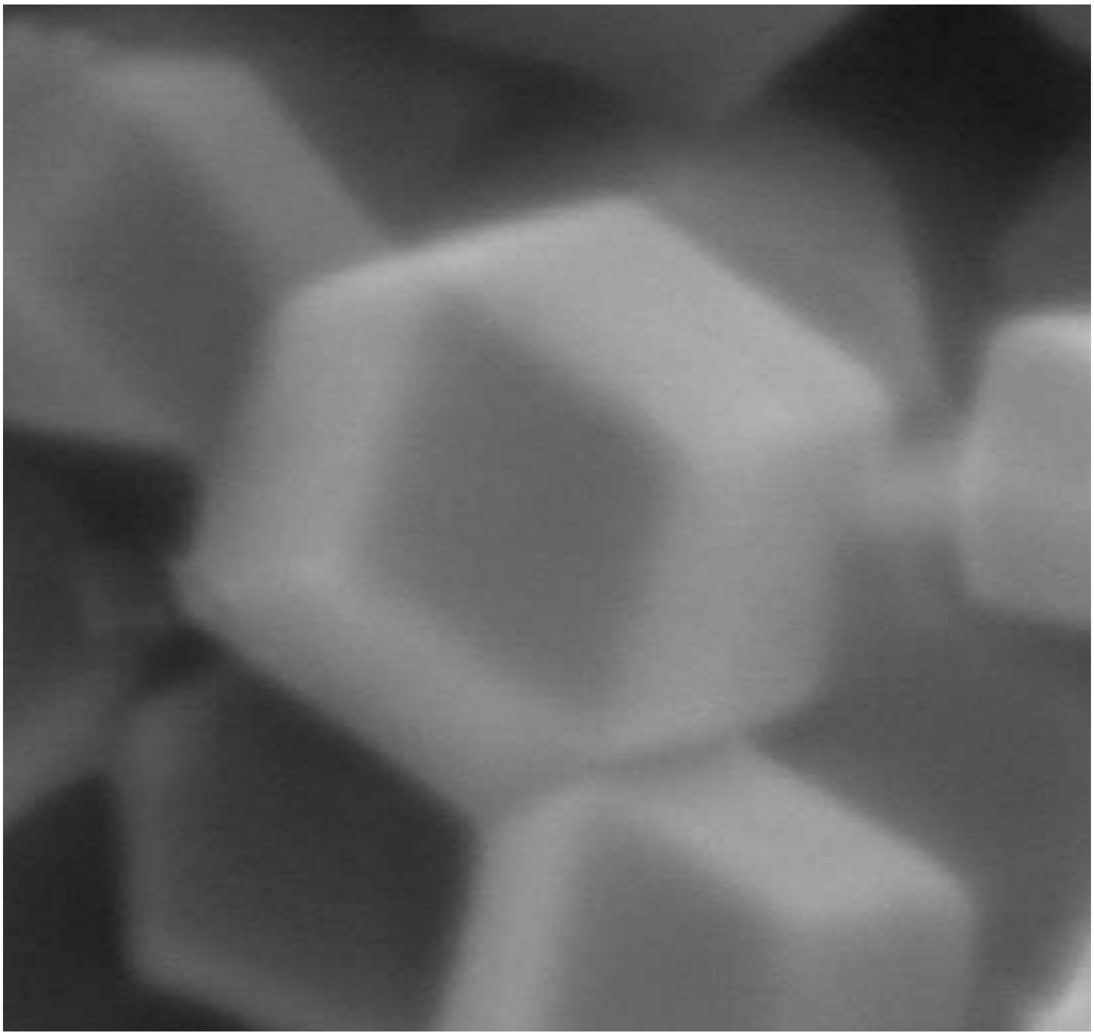

Figure S21. SEM image of a pristine ZIF-8. The rhombic dodecahedral morphology is shown with exposing the 12 (011) faces.

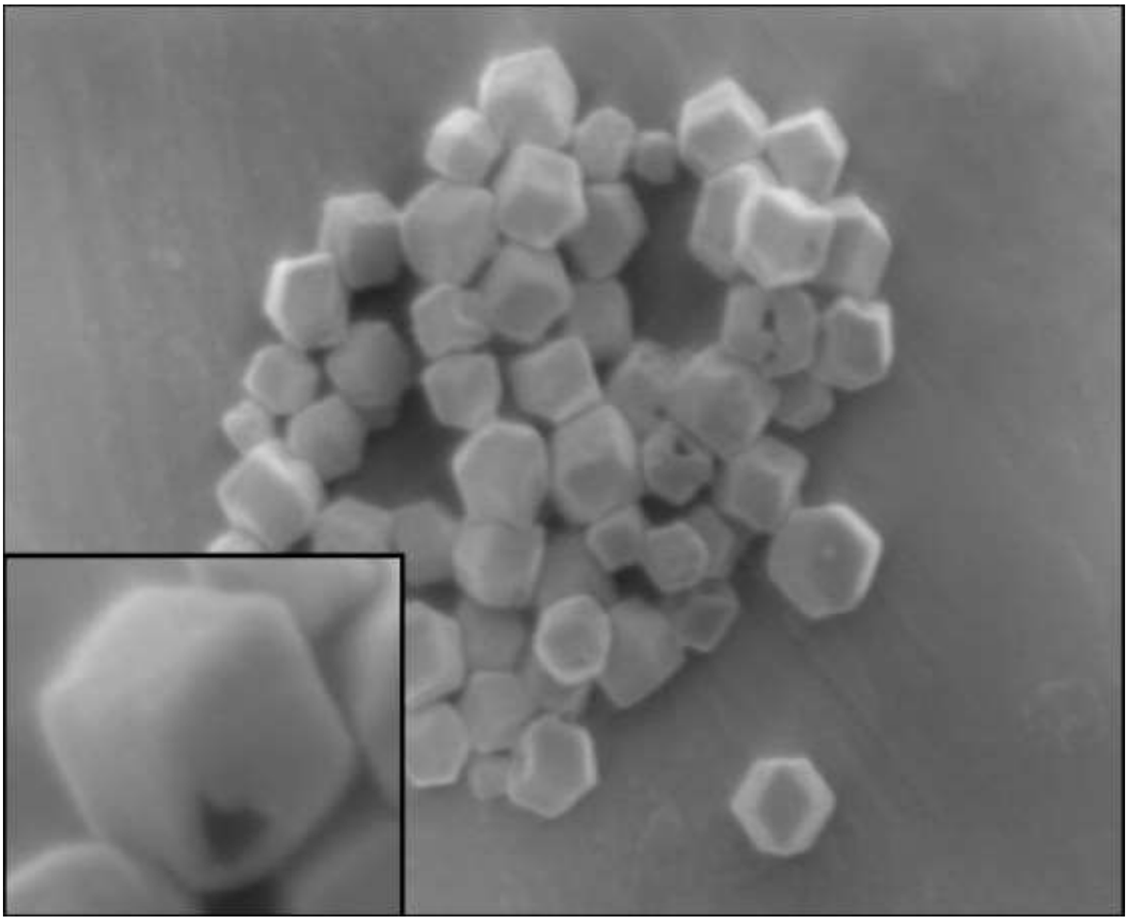

Figure S22. SEM images of H-ZIF-8[1S]. A dark area proves an empty space caused by a hollow structure.

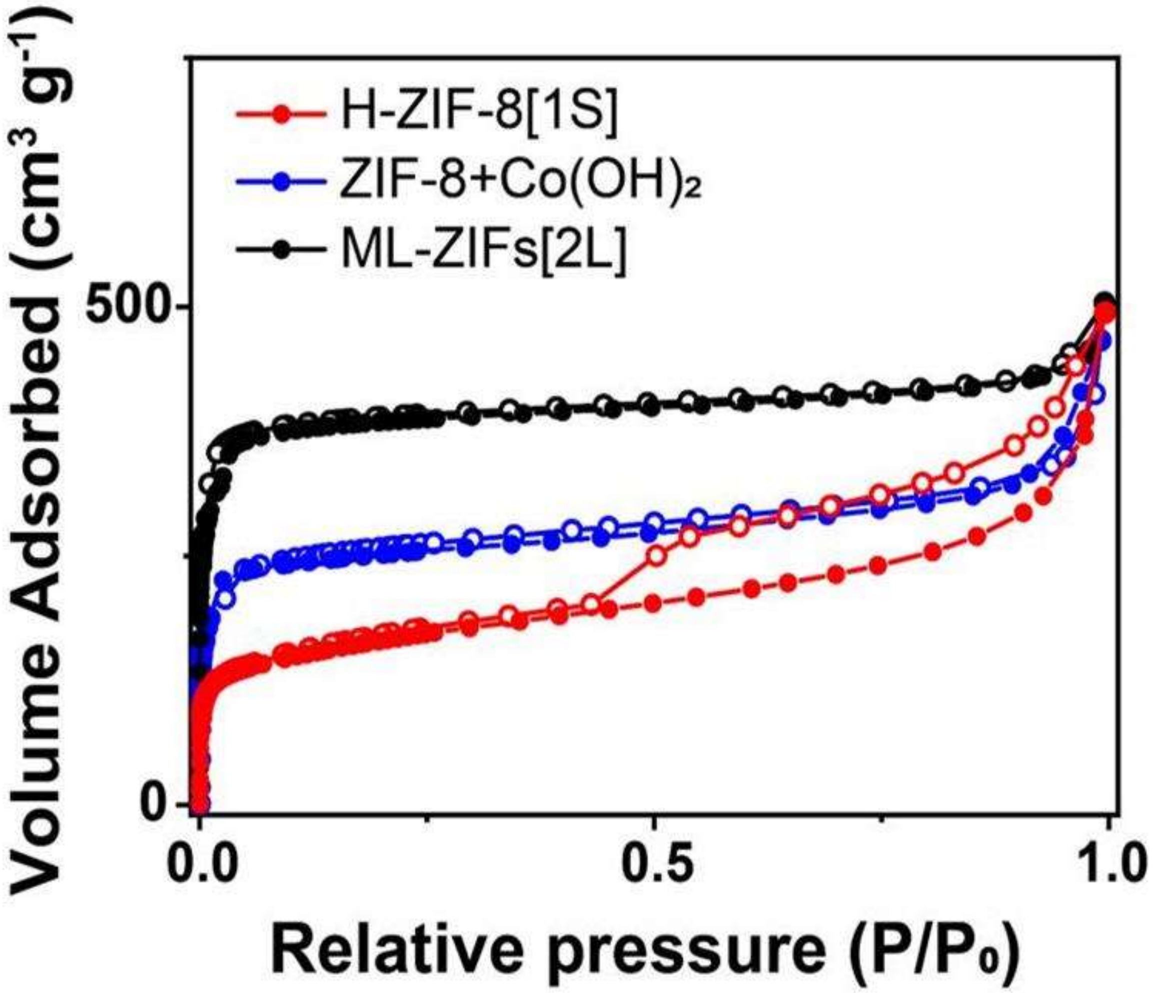

Figure S23. N_2 **adsorption isotherm of H-ZIF-8[1S],** $ZIF\text{-}8+Co(OH)_2$ **and ML-ZIFs[2L].**

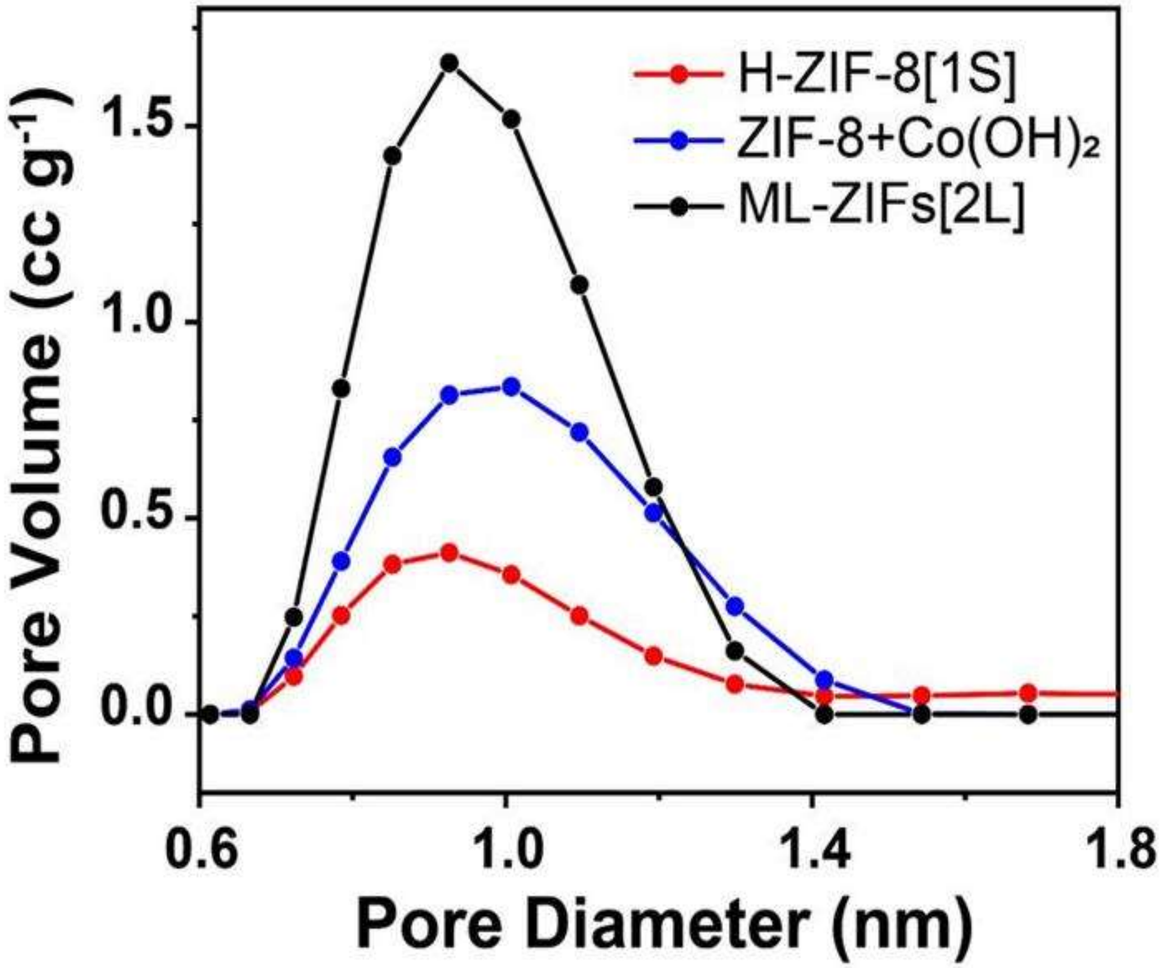

Figure S24. Pore size distribution calculated by the DFT method.

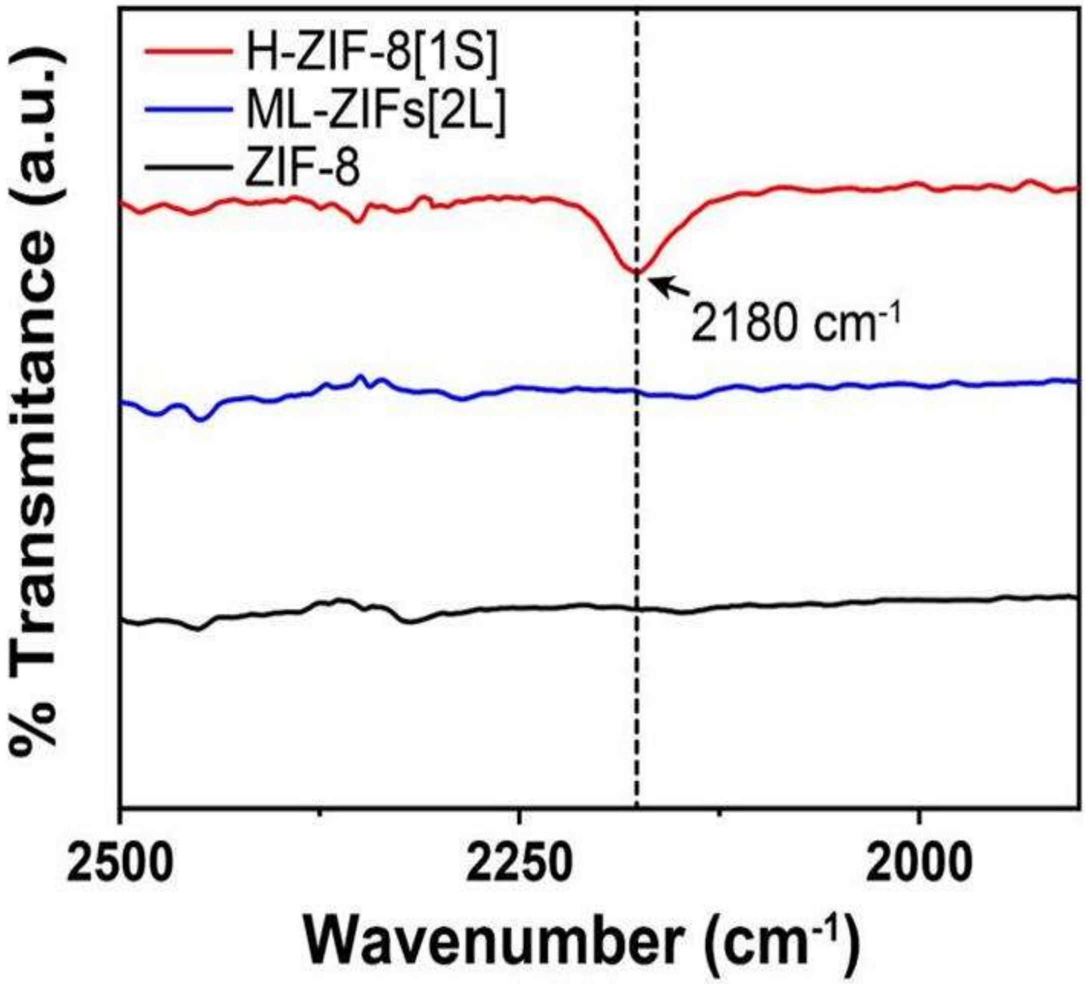

Figure S25. IR spectra of H-ZIF-8[1S], ML-ZIFs[2L] and ZIF-8. A peak at 2180 cm^{-1} supports that the more oxidized Co is binding with CO.

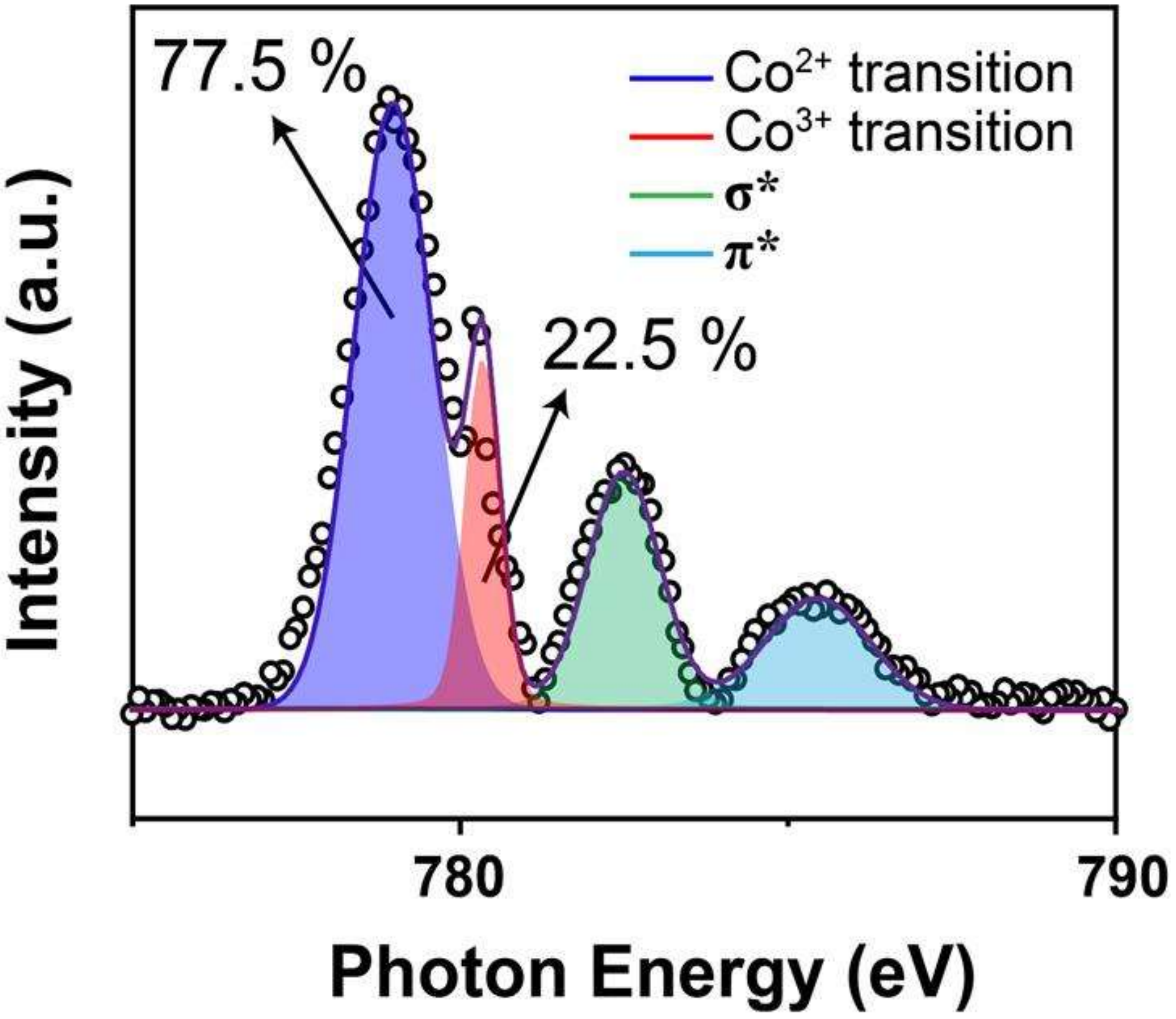

Figure S26. Co L_3-edge NEXAFS spectrum. The Co^{2+} and Co^{3+} transitions occupy 85.9 % and 14.1 %, respectively.

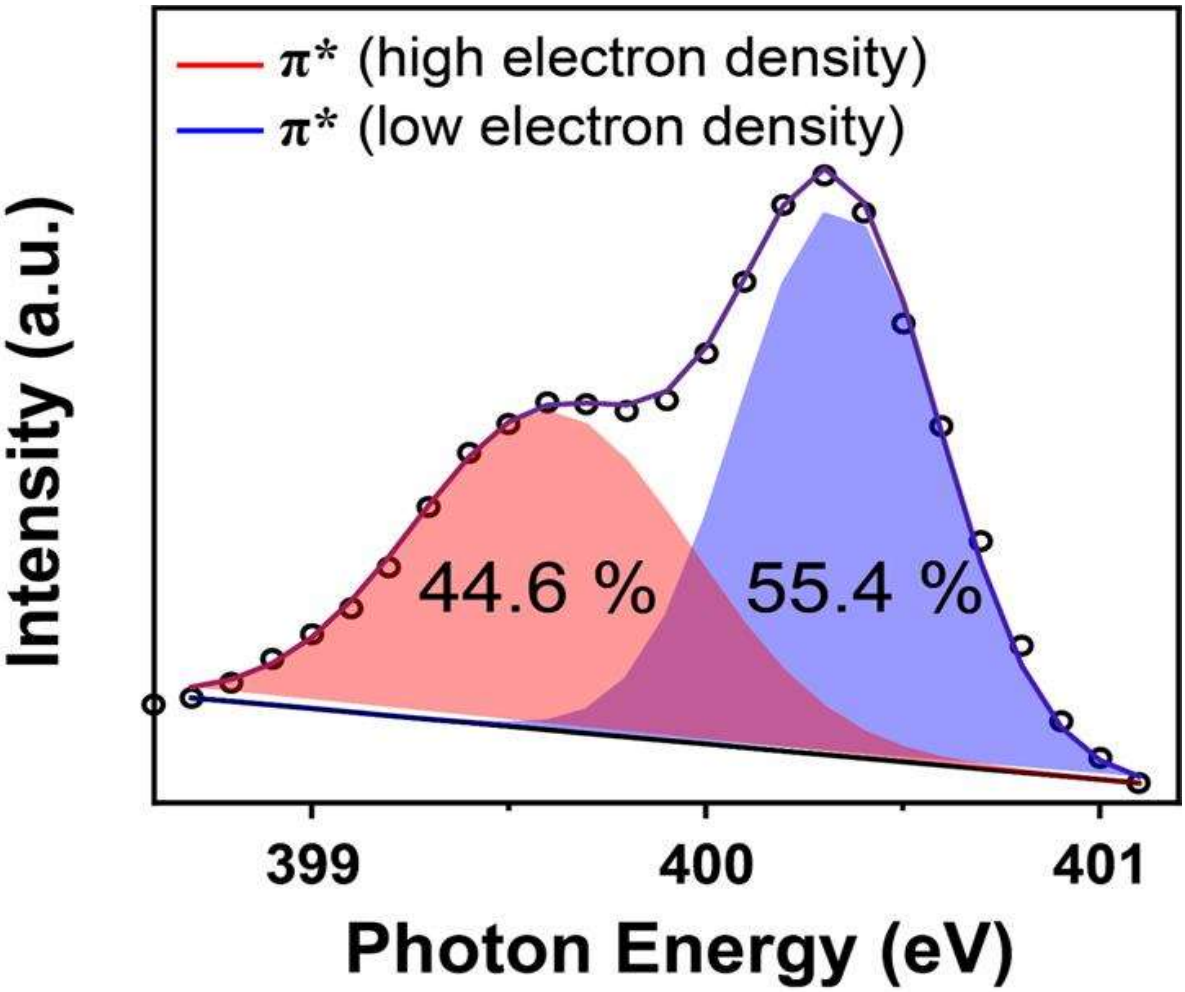

Figure S27. N K-edge NEXAFS spectrum. The π* transition having high electron density occupies 44.6 % and that of low electron density occupies the rest.

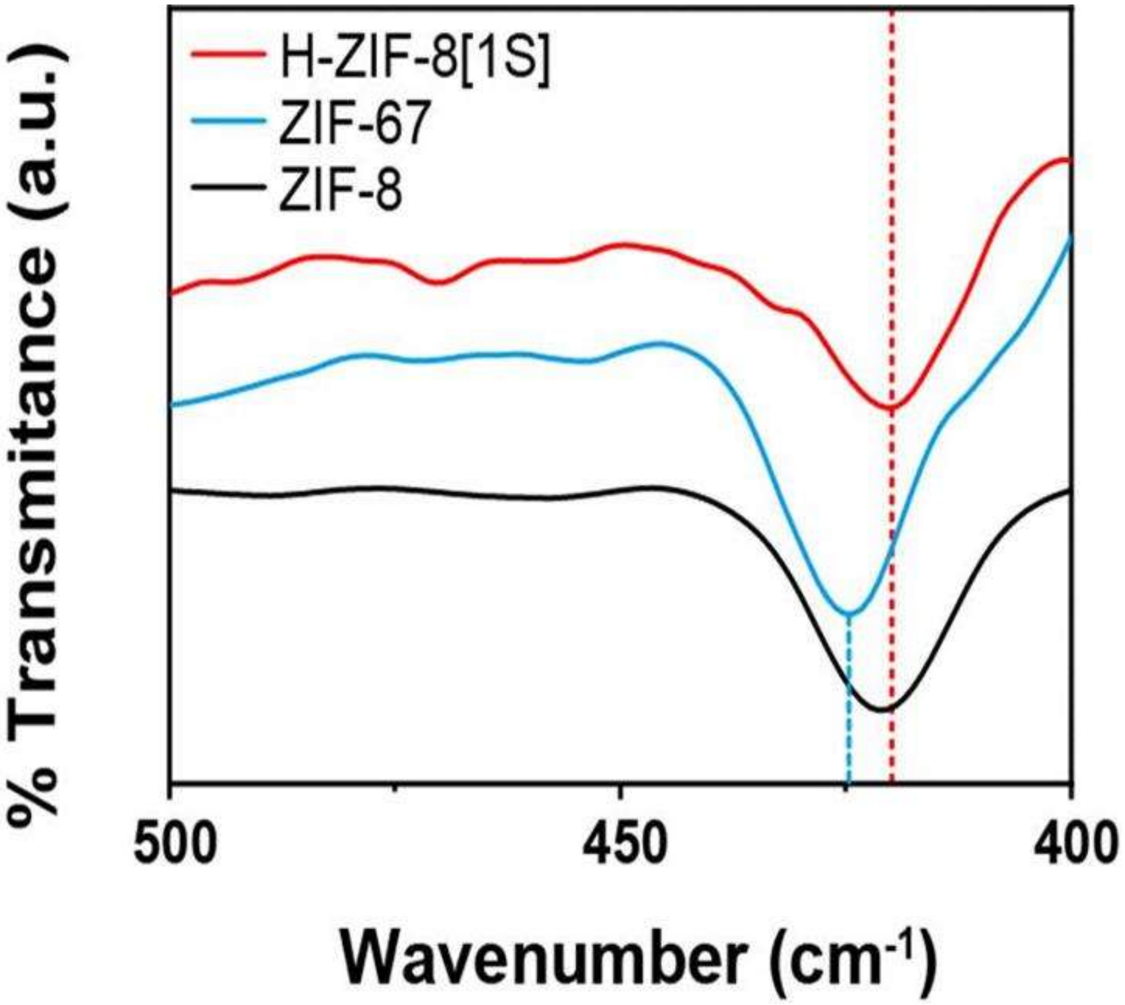

Figure S28. IR spectra in the finger print region. The red and blue lines represent Zn-N and Co-N bonds, respectively.

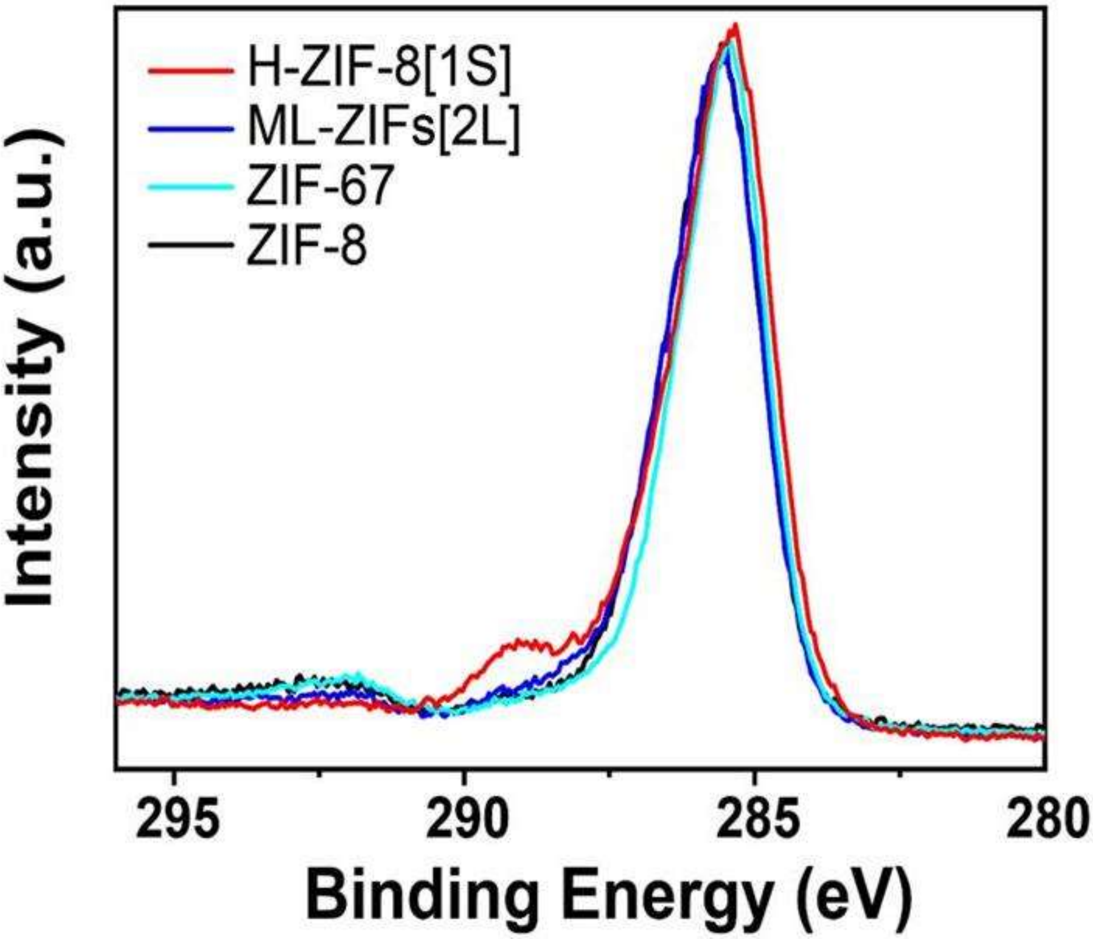

Figure S29. **C 1s XPS spectra of H-ZIF-8[1S], ML-ZIFs[2L] and ZIF-8.** A small peak of H-ZIF-8[1S] is related to CO with Co^{3+} at 289 eV. All peaks are shifted by 285.4 eV for the sp^2 C=N bonding.

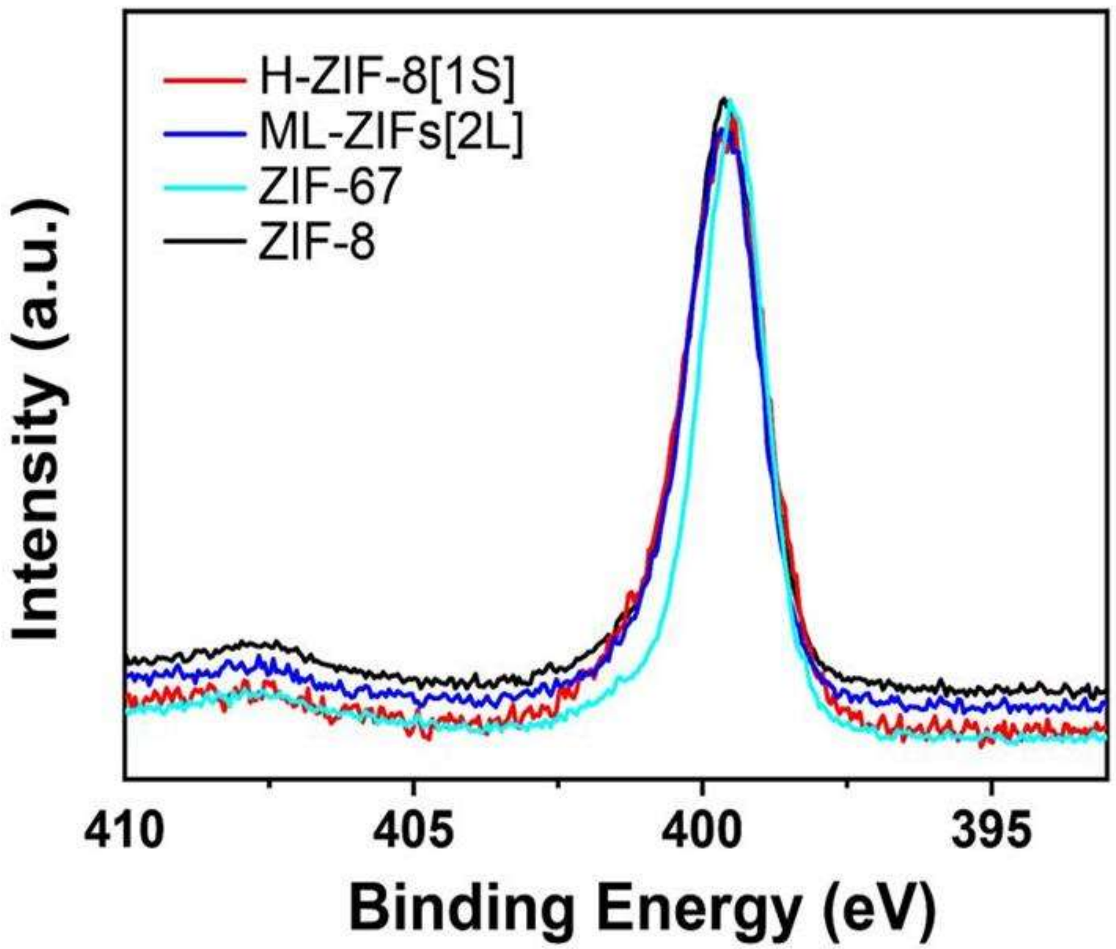

Figure S30. N 1s XPS spectra of H-ZIF-8[1S], ML-ZIFs[2L], ZIF-67 and ZIF-8. A single peak at 399.4 eV is related to the pyridinic nitrogen of 2-mim. All the peaks are shifted by 285.4 eV for the sp^2 C=N bonding.

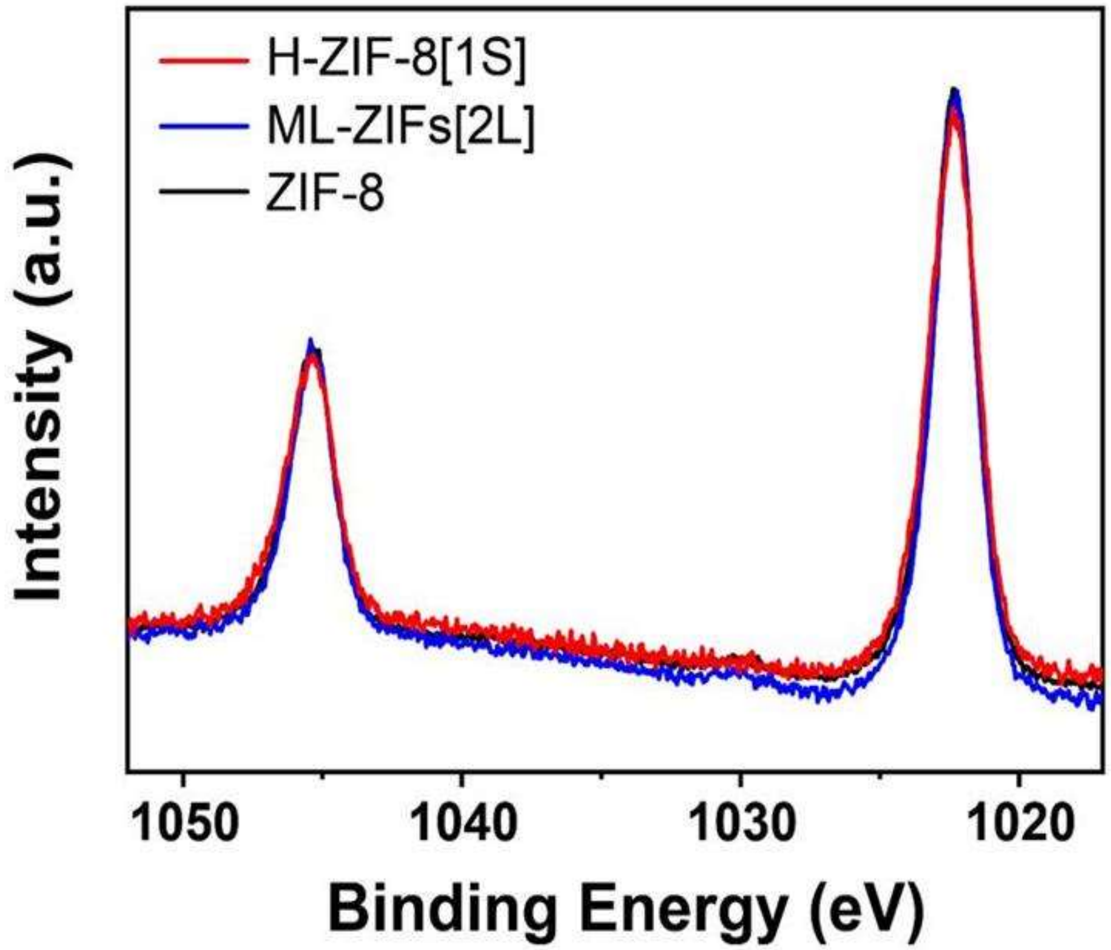

Figure S31. Zn 2p XPS spectra of H-ZIF-8[1S], ML-ZIFs[2L], ZIF-67 and ZIF-8. A $2p_{2/3}$ peak at 1223 eV and a $2p_{1/2}$ peak at 1246 are related to Zn-N bonding. All the peaks are shifted by 285.4 eV for the sp^2 C=N bonding.

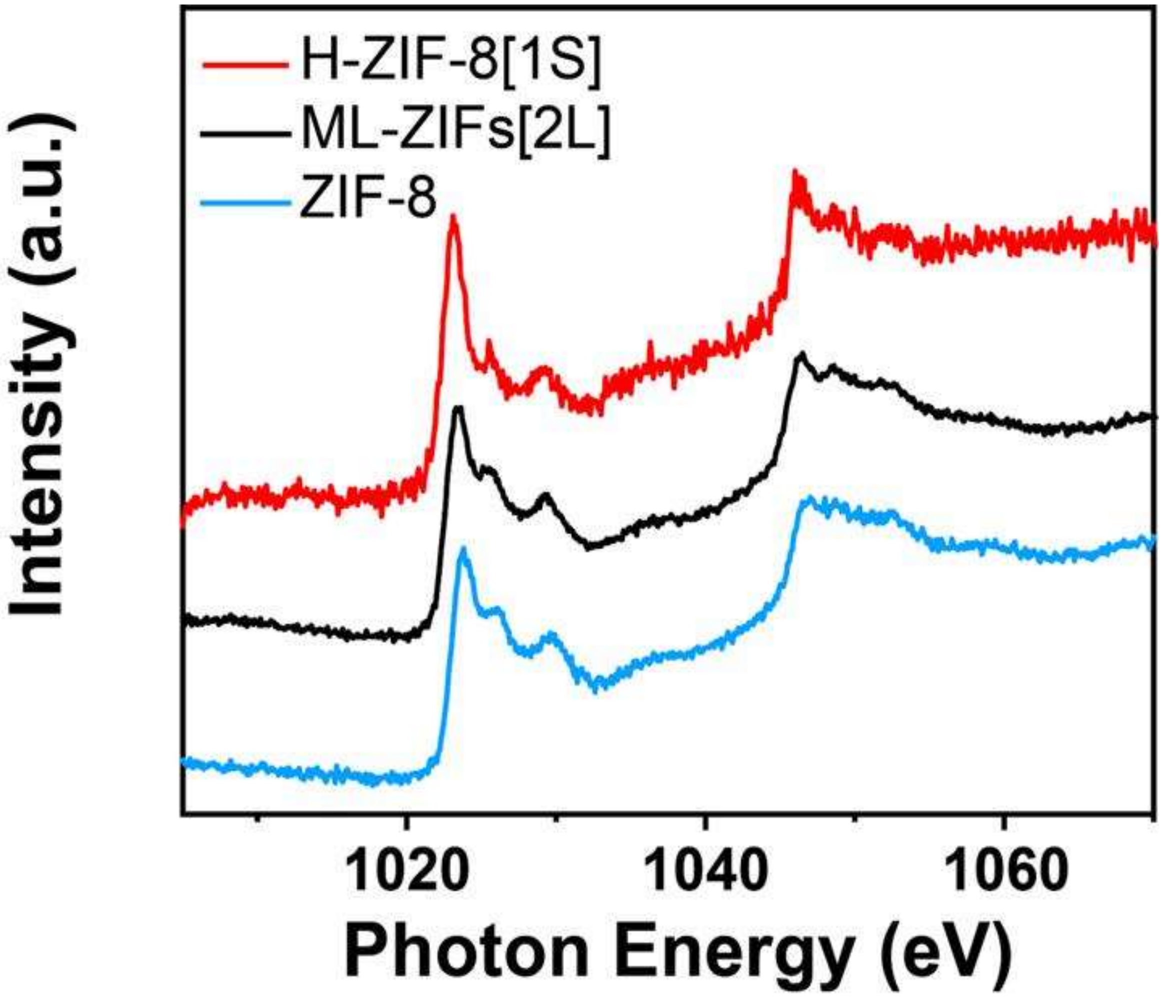

Figure S32. Zn L-edge NEXAFS spectra.

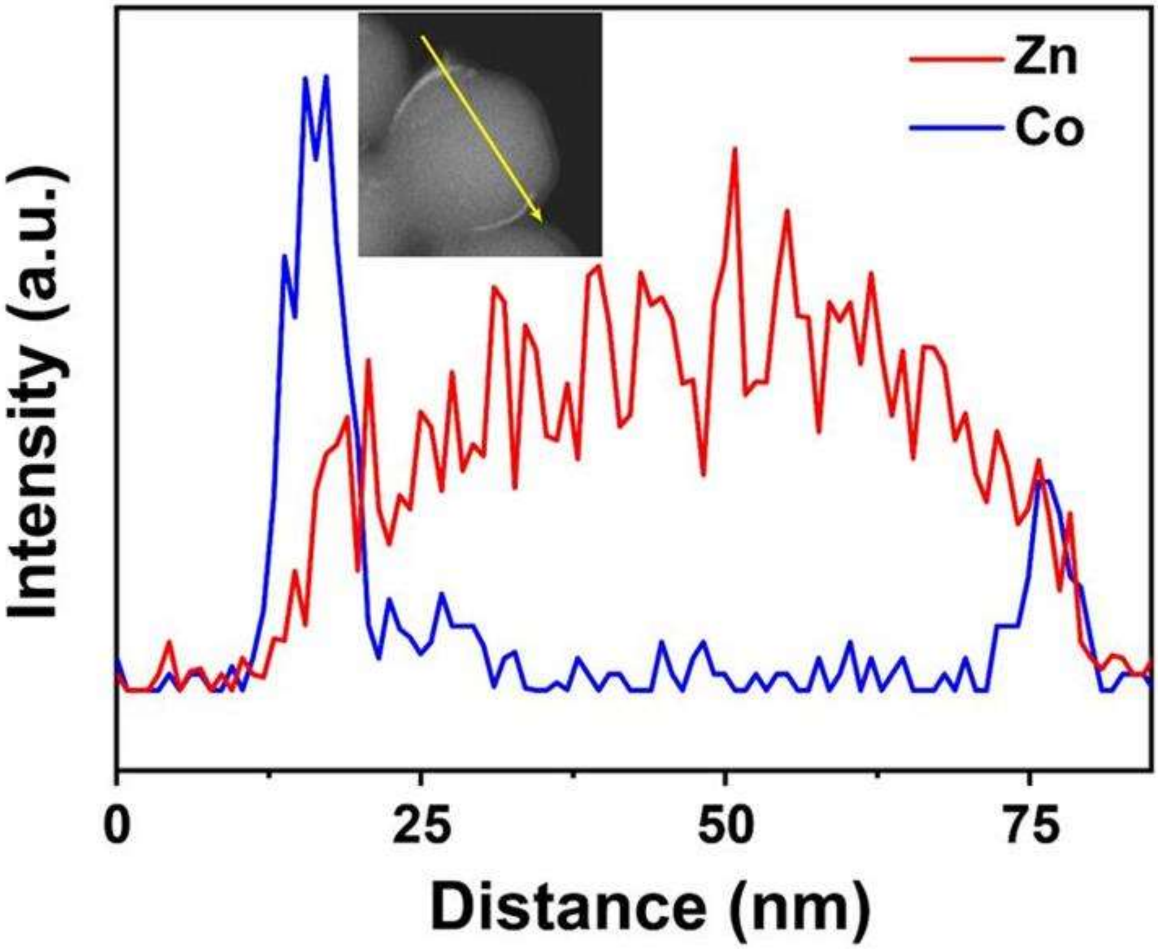

Figure S33. Line profiling graph of the HAADF-STEM image corresponding to ZIF-8 with Co^{2+} in methanol.

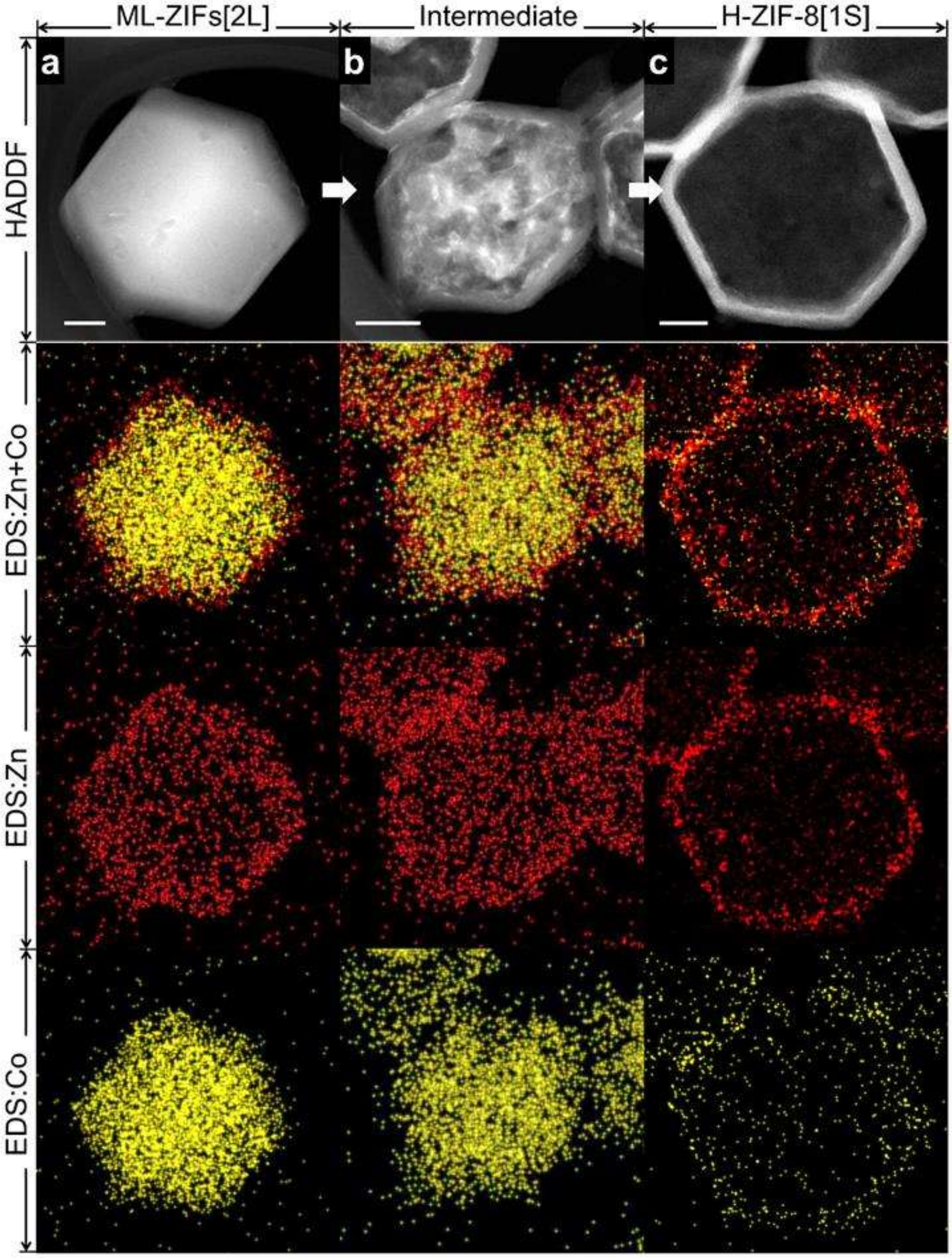

Figure S34. HADDF-STEM image and corresponding EDS images showing the process of making H-ZIF-8[1S].

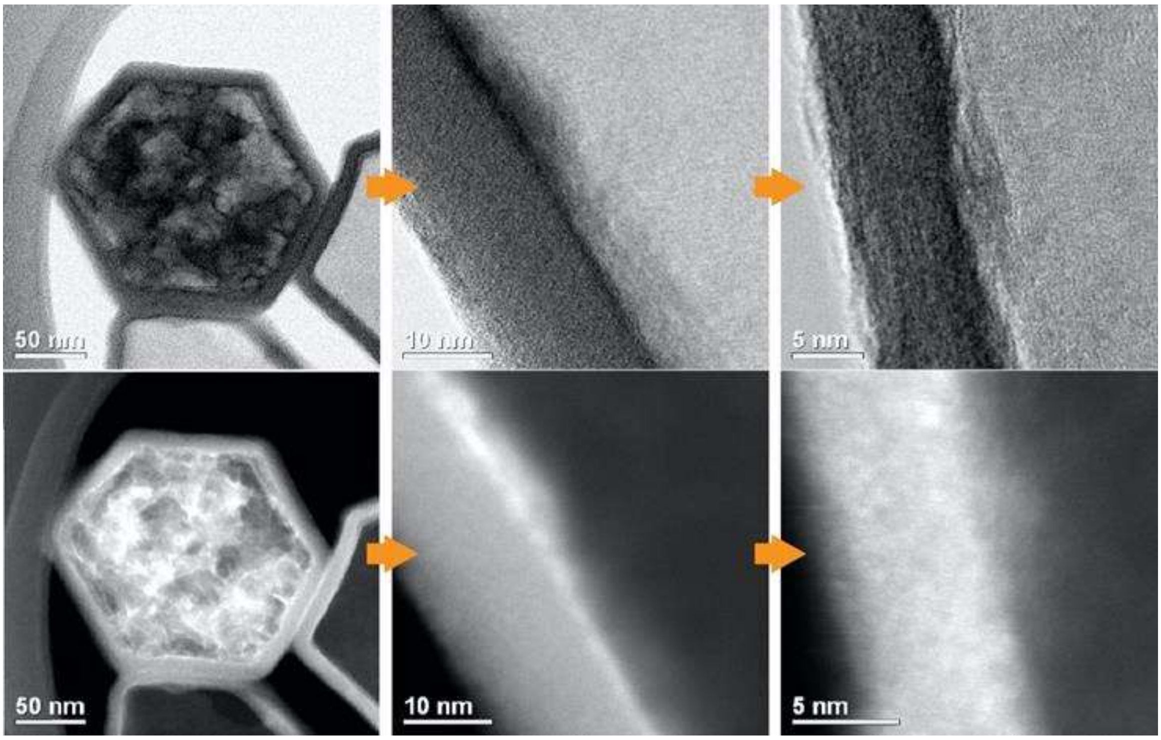

Figure S35. TEM images at intermediate stage and final stage of ML-ZIFs[2L] and H-ZIF-8[1S].

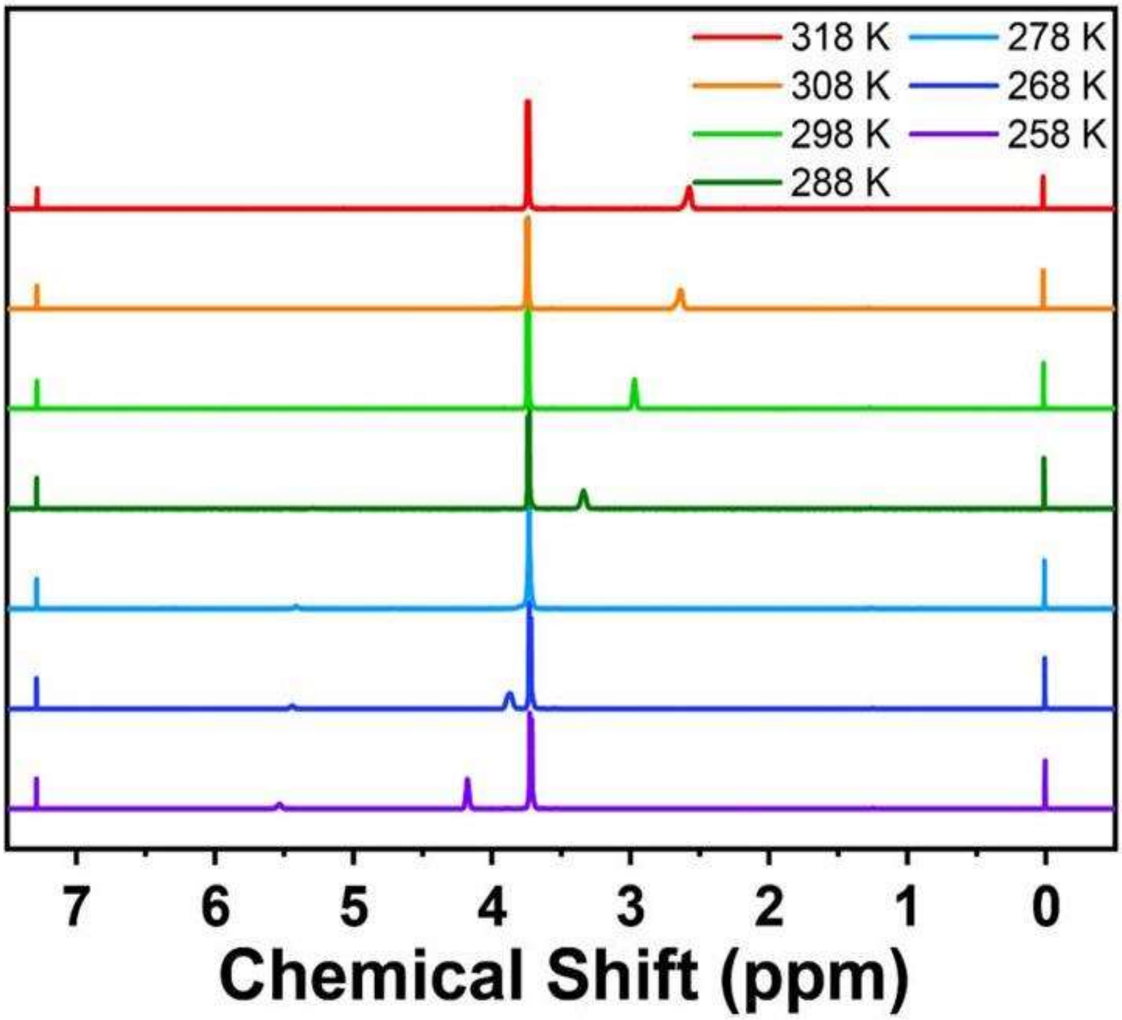

Figure S36. ^{1}H-NMR spectra of pure EG according to temperature change. All the spectra were obtained with the inverse-gated decoupling and zg30. A relaxation delay time is 5 s and the acquisition time is 8.2 s. The deuterated chloroform (7.26 ppm) was used as the solvent with tetramethylsilane (0 ppm).

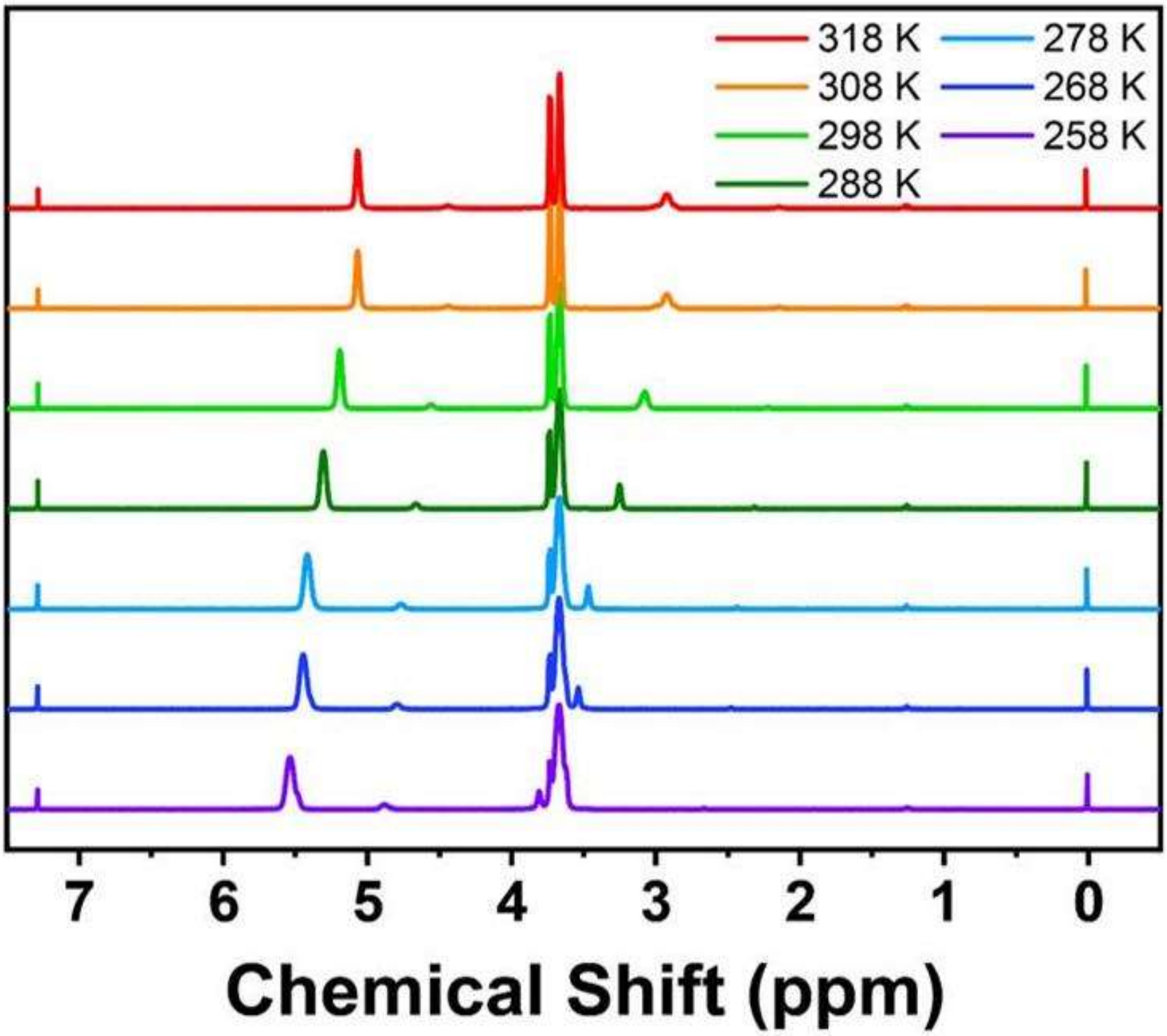

Figure S37. ^{1}H-NMR spectra of EG-water according to temperature change. All the spectra were obtained with inverse-gated decoupling and zg30. A relaxation delay time is 5 s and the acquisition time is 8.2 s. The deuterated chloroform (7.26 ppm) was used as the solvent with tetramethylsilane (0 ppm).

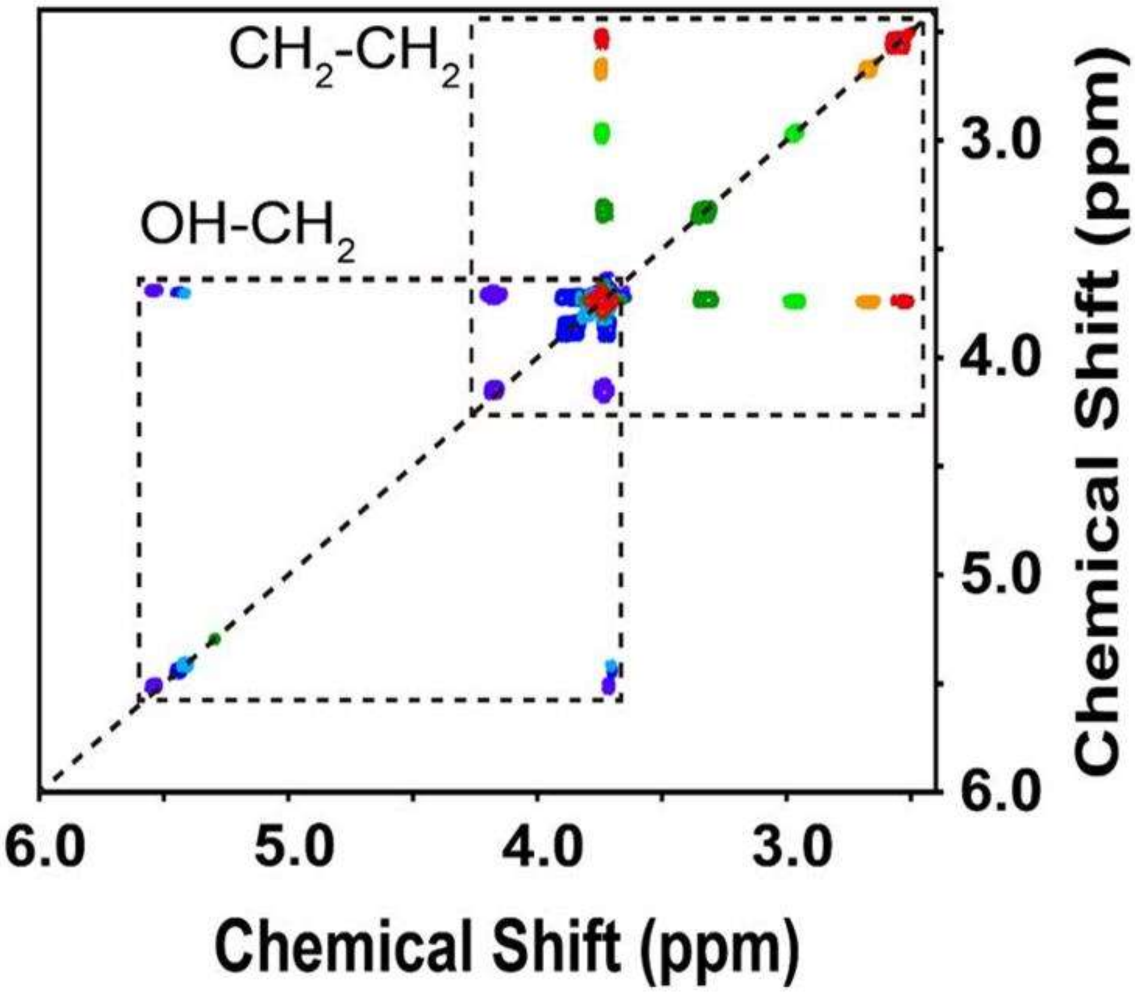

Figure S38. COSY spectra of a pure EG according to temperature change. The x, y axes of COSY spectra are matched with the ^{1}H NMR chemical shift and the cross peaks are seen on both sides of the diagonal due to spin-spin coupling between protons. All the spectra were obtained with the inverse-gated decoupling. All 2D COSY experiments used the cosygpppqf and data point of 1024(F2)/128(F1). After acquisition, the data were zero filled and fourier transformed, and the baselines were corrected in f2 and f1. Temperature: red (318K), orange(308K), light green (298K), dark green (288K), sky-blue (278K), blue (268K) and violet (258K).

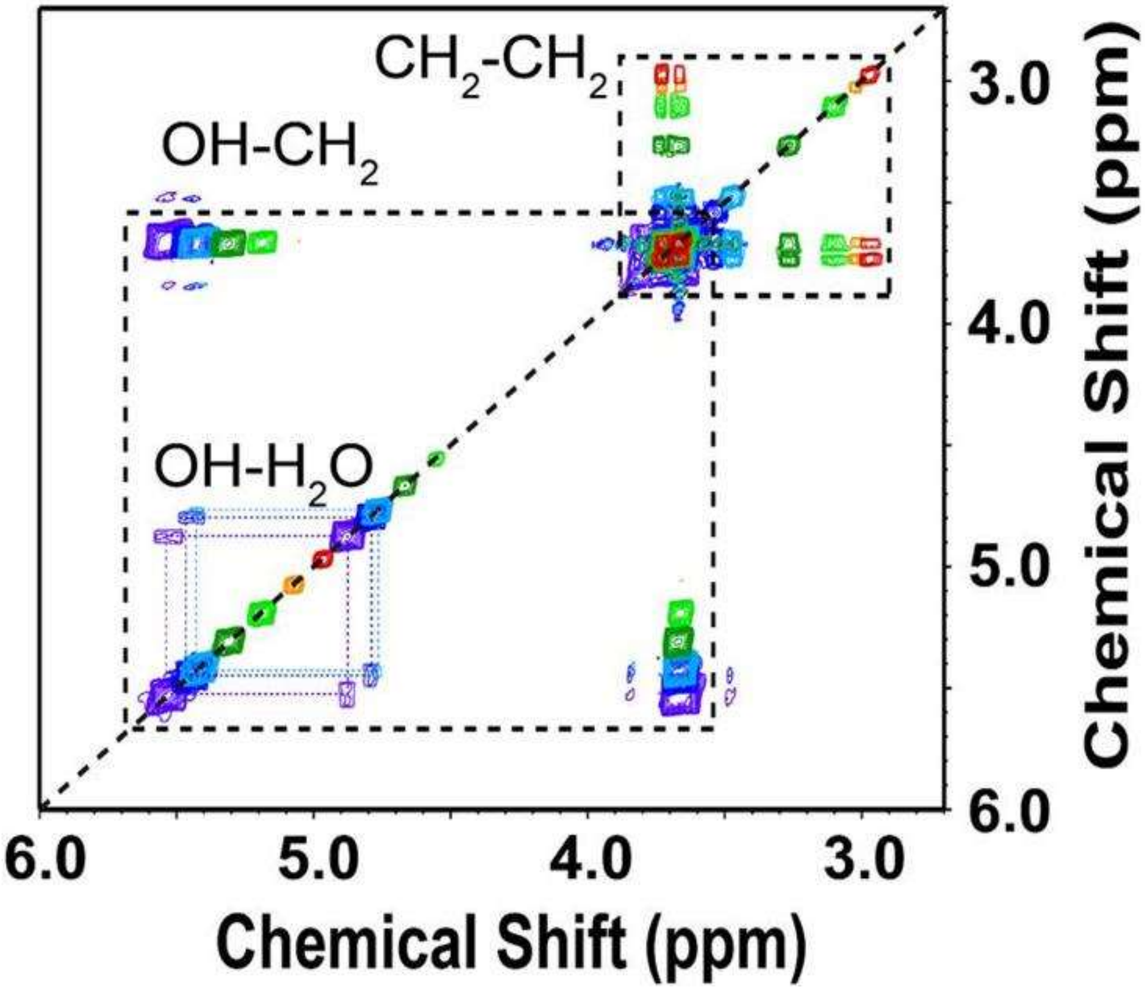

Figure S39. COSY spectra of EG-water according to temperature change. The x, y axes of COSY spectra are matched with the ^{1}H NMR chemical shift and the cross peaks are seen on both sides of the diagonal due to spin-spin coupling between protons. All the spectra were obtained with the inverse-gated decoupling. All 2D COSY experiments used the cosygpppqf and data point of 1024(F2)/128(F1). After acquisition, the data was zero filled and fourier transformed, and the baselines were corrected in f2 and f1. Temperature: red (318K), orange (308K), light green (298K), dark green (288K), sky-blue (278K), blue (268K) and violet (258K).

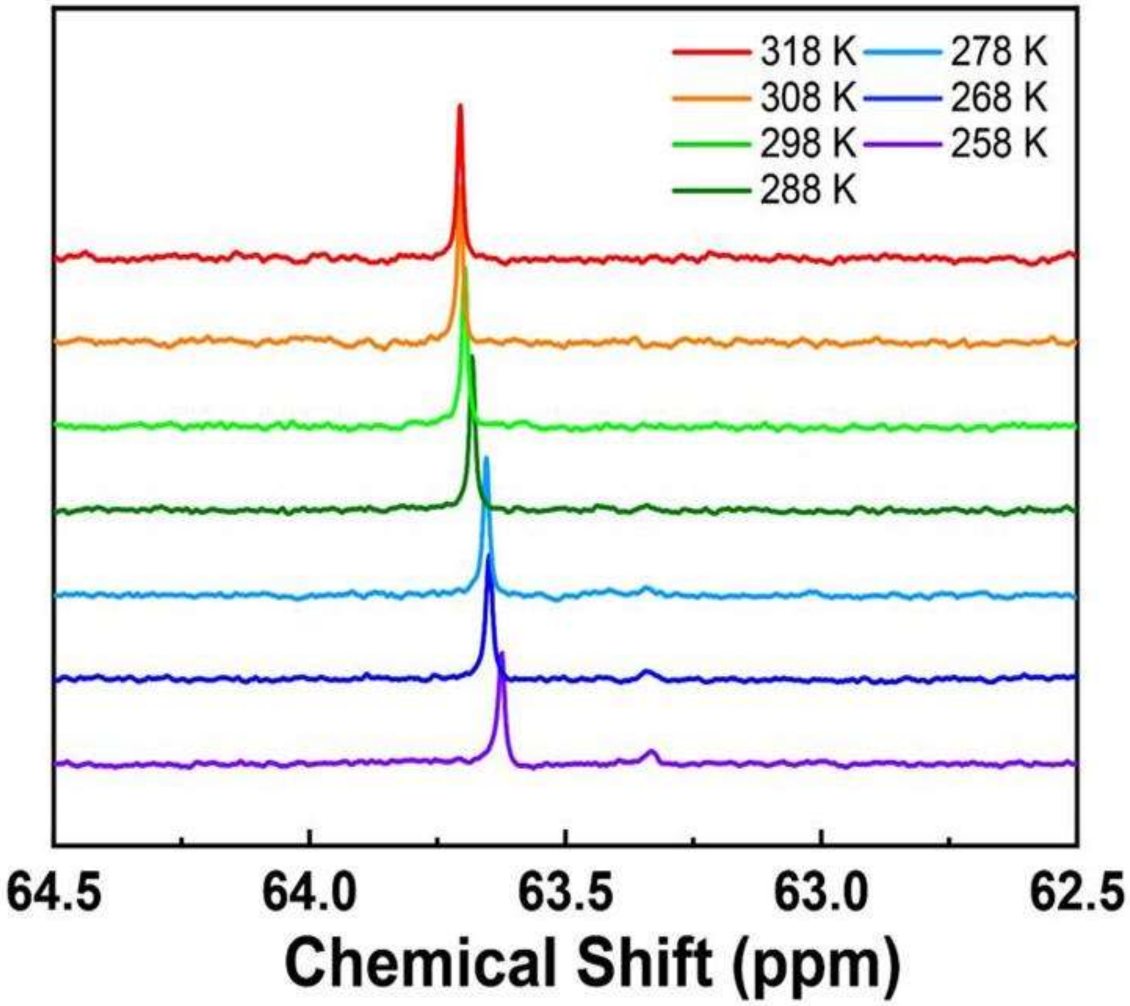

Figure S40. ^{13}C-NMR spectra of a pure EG according to temperature change. Only one sharp peak appears at 63.6-63.7 ppm. All the spectra were obtained with the inverse-gated decoupling and zgig30. A relaxation delay time is 30 s and the acquisition time is 2.7 s. The deuterated chloroform (7.26 ppm) was used as the solvent with tetramethylsilane (0 ppm).

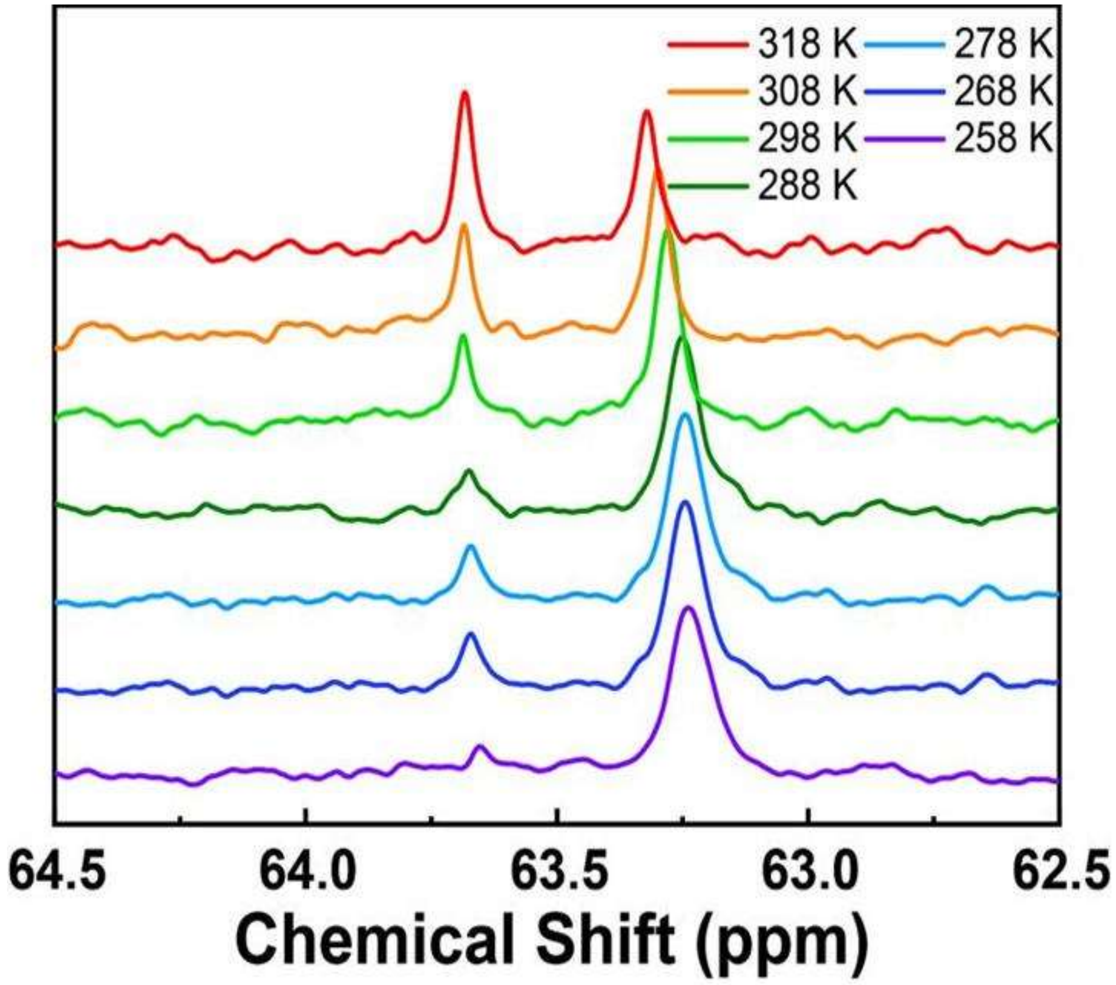

Figure S41. ^{13}C-NMR spectra of EG-water according to temperature change. A broad peak appears at 63.2-63.3 ppm. All the spectra were obtained with the inverse-gated decoupling and zgig30. A relaxation delay time is 30 s and the acquisition time is 2.7 s. The deuterated chloroform (7.26 ppm) was used as the solvent with tetramethylsilane (0 ppm). At the EG freezing point (258 K), only a broad peak is observed resulting from the conformational transformation of EG, as explained by the Karplus equation, which describes the correlation between the ^{3}J-coupling constants and dihedral torsion angle

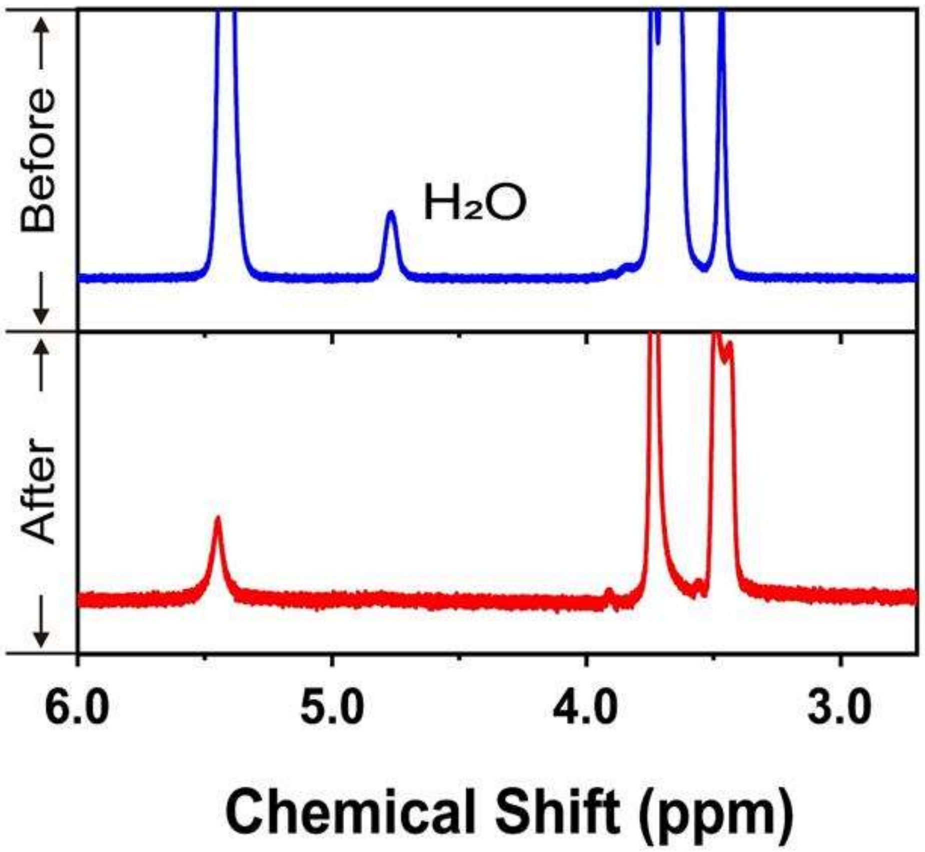

Figure S42. **^{1}H-NMR spectra after water molecules transfer at 273 K..**

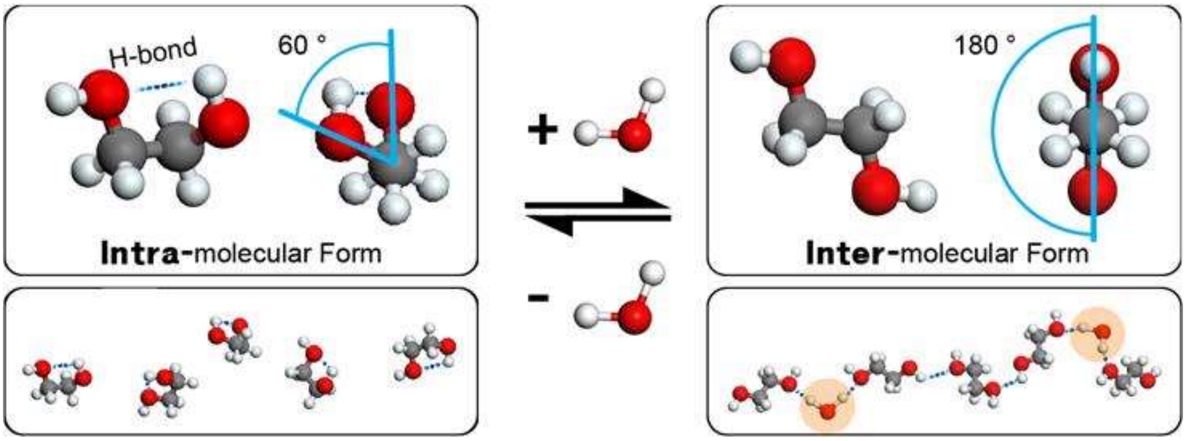

Figure S43. Conformations of EG depends on the presence of water molecule.

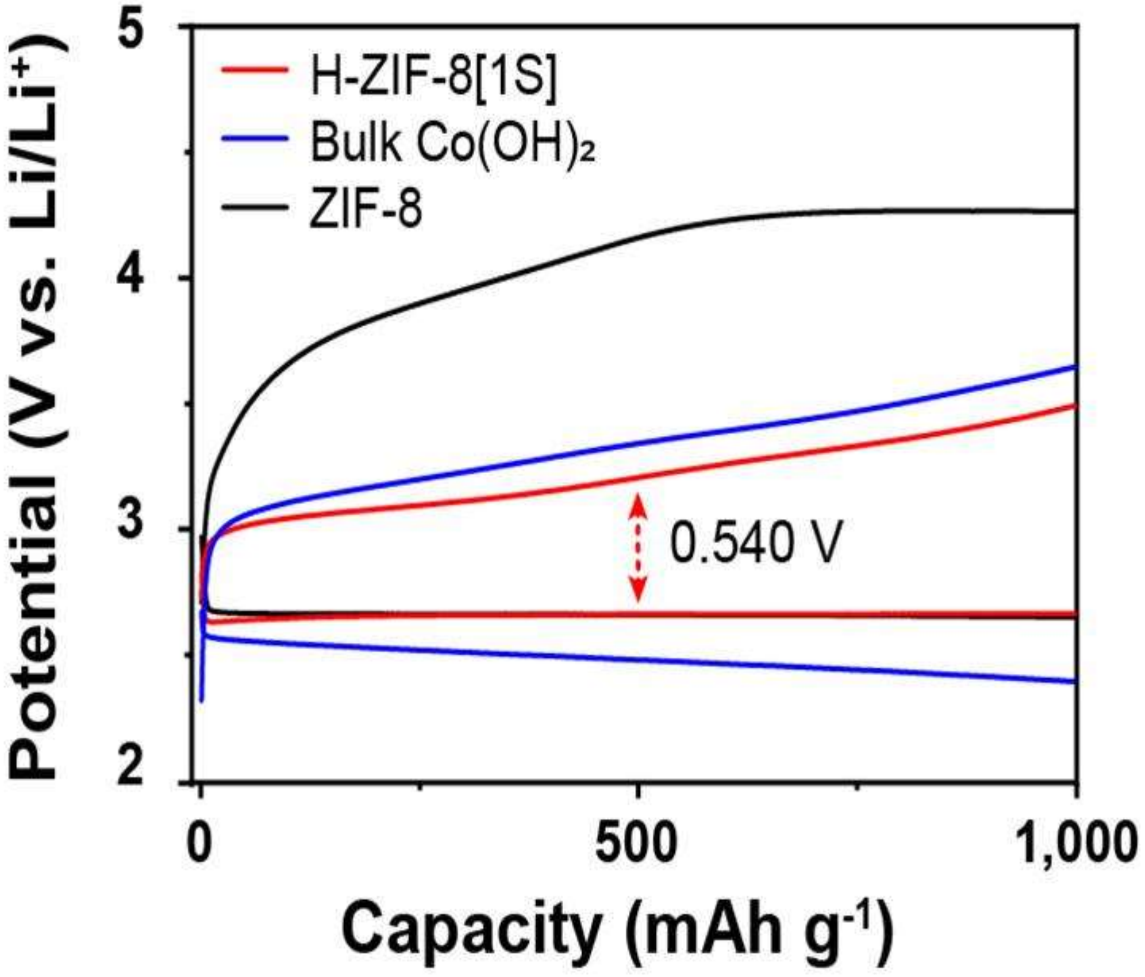

Figure S44. Gravimetric discharge and charge curves at a current density of 50 mA g^{-1} with a cut-off capacity of 1,000 mAh g^{-1}.

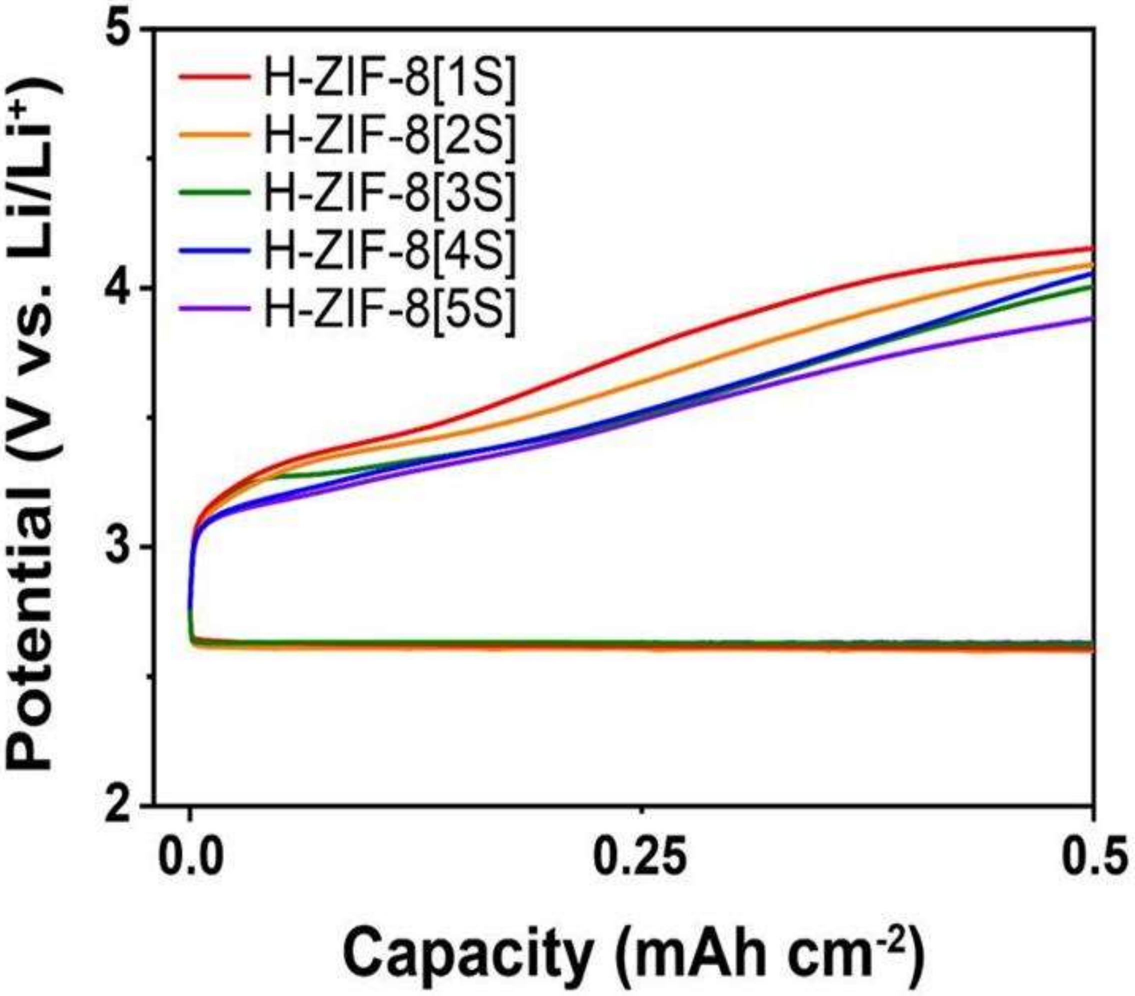

Figure S45. Discharge and charge curves of H-ZIF-8[nS] at a constant current of 0.1 mA cm^{-2} with the cut-off capacity of 0.5 mAh cm^{-2}.

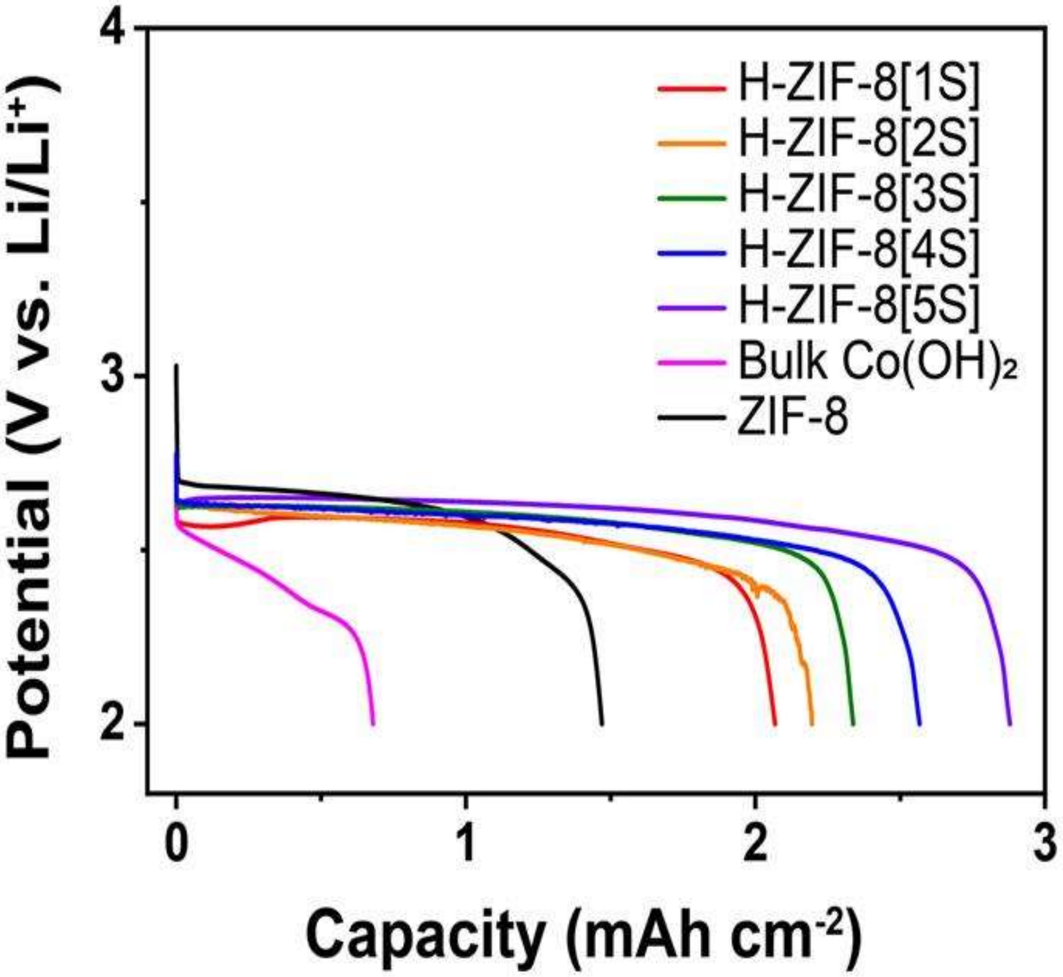

Figure S46. Full geometric discharge curves at a constant current density of 0.1 mA cm^{-2}.

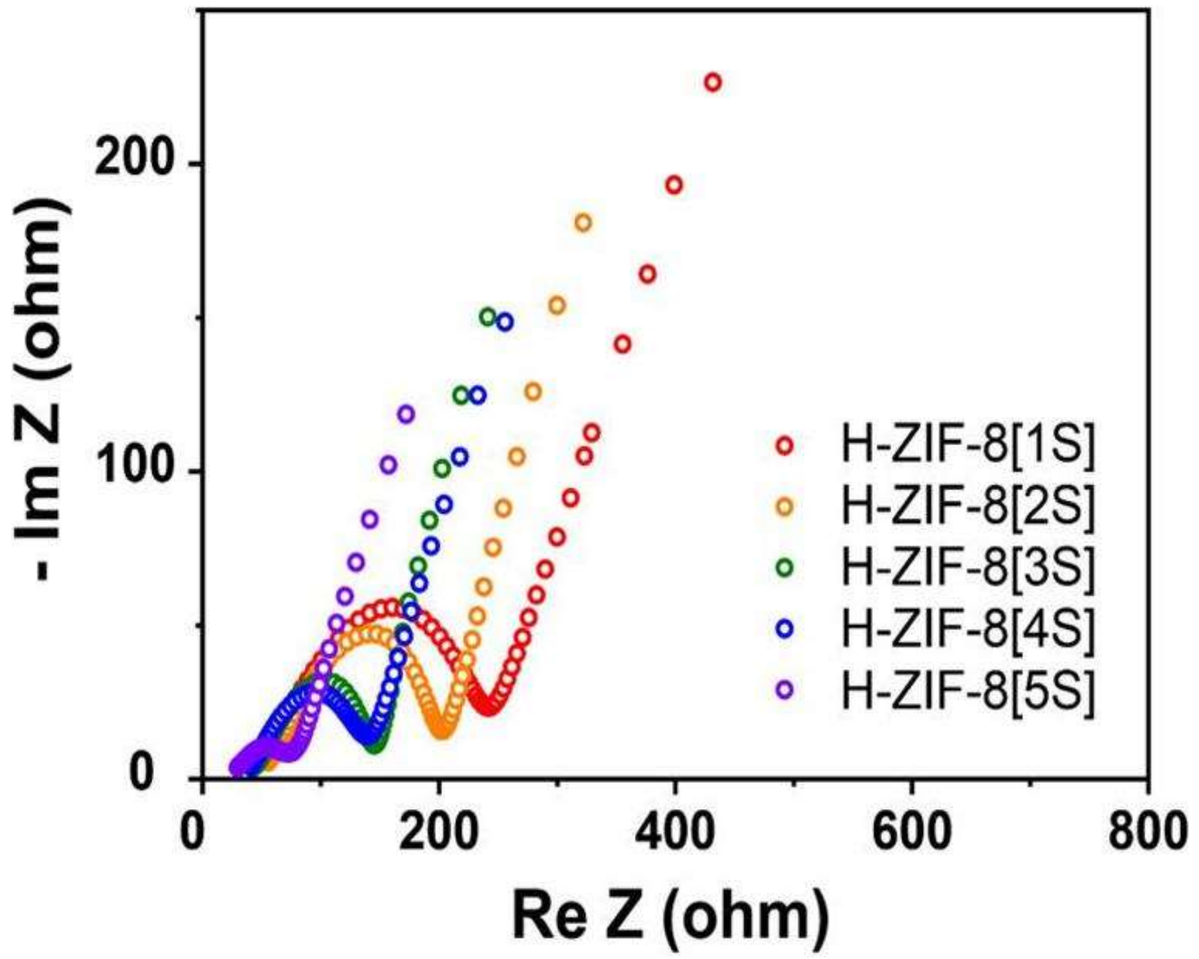

Figure S47. Nyquist plots of H-ZIF-8[nS]. All the measurements were conducted after exposed O_2 for 6 hours. The amplitude was 10 mV in the frequency range from 0.1 to 10^5 Hz.

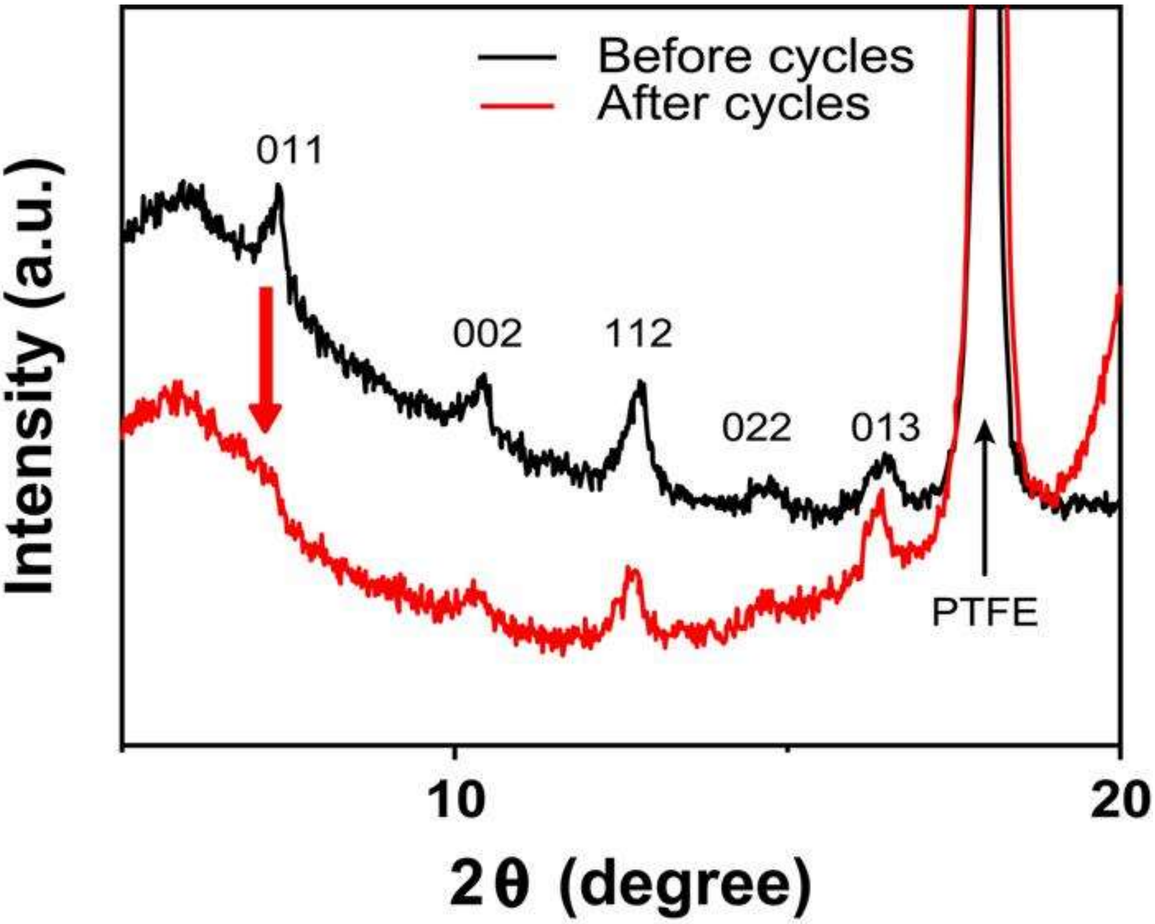

Figure S48. XRD patterns of H-ZIF-8[1S] before and after electrochemical reactions.

Element	At. No.	Mass (%)	Atom (%)	Abs. error (%)
Cobalt	27	67.39	69.63	6.31
Zinc	30	32.61	30.37	3.21

Table S1. Weight % and atomic % corresponding to the EDX mapping image of ML-ZIFs[2L].

Element	At. No.	Mass (%)	Atom (%)	Abs. error (%)
Cobalt	27	31.89	34.19	3.75
Zinc	30	68.11	65.81	7.24

Table S2. Weight % and atomic % corresponding to the EDX mapping image of H-ZIF-8[1S].

**Multilayer/Multishell MOFs**	**Cobalt (mg/kg)**	**Zinc (mg/kg)**
**ML-ZIFs[2L]**	73562	180880
**H-ZIF-8[1S]**	103574	92200

Table S3. Weight % estimated from the ICP-OES analysis for ML-ZIFs[2L] and H-ZIF-8[1S].

	Co-O	Co-Co
Bulk	1	1.79
6-membered	1	0.85
Tri-nuclear	1	0.60
Dinuclear	1	0.30
Mono-nuclear	1	0
H-ZIF-8[1S]	1	0.40

Table S4. Intensity ratio derived from Co K-edge EXAFS simulation curves with $Co(OH)_2$ size obtained by back Fourier transformation of the radial structural functions.

Samples	Temperature (K)	Intra-molecular form		INTER-molecular form	
		Diffusion coefficient (10^{-9} m^2 s^{-1})	Standard deviation	Diffusion coefficient (10^{-9} m^2 s^{-1})	Standard deviation
Pure EG	278	1.04	0.0468	x	X
	298	1.29	0.0182	x	X
	318	9.83	0.00750	x	X
EG-water	278	1.58	0.0212	522	0.0212
	298	1.57	0.0159	14.5	0.0159
	318	8.34	0.0239	32.1	0.0239

Table S5. Diffusion coefficient values of pure EG and EG-water estimated from ^{13}C DOSY. All the ^{13}C DOSY experiments used stebpgpin1s and a series of 24 spectra were collected with the relaxation delay time is 60 s, gradient duration 1200 µs , diffusion time 0.1 s, linear gradient ramp from 2 to 95 % of the maximum gradient strength. After acquisition, the DOSY data was zero filled and fourier transformed, and the baseline was corrected in f2. The diffusion coefficients were calculated with the T1/T2 relaxation module using monoexponential fitting on Bruker Topspin v3.5.

	Rate(weight)	Rate(Areal)	Capacity	Cycles
H-ZIF-8[1S]	1.396	0.645	0.770	0.766
H-ZIF-8[2S]	X	0.655	0.831	0.813
H-ZIF-8[3S]	X	0.779	0.851	0.864
H-ZIF-8[4S]	X	0.823	0.869	1.063
H-ZIF-8[5S]	X	0.879	0.884	1.200
$Co(OH)_2$	1.301	x	0.901	1.064
ZIF-8	1.311	x	0.671	0.628

Table S6. Mass loadings of cathodes (mg cm^{-2}).

Samples	R_s (Ω)	R_{ct} (Ω)
H-ZIF-8[1S]	54.057	197.21
H-ZIF-8[2S]	55.886	153.47
H-ZIF-8[3S]	46.421	102.56
H-ZIF-8[4S]	41.297	102.36
H-ZIF-8[5S]	25.516	56.731
$Co(OH)_2$	60.134	140.64
ZIF-8	59.824	196.8

Table S7. R_s and R_{ct} estimated from EIS experiments.
